# Beyond cancer genes: colorectal cancer as robust intrinsic states formed by molecular interactions

**DOI:** 10.1098/rsob.170169

**Published:** 2017-11-08

**Authors:** Ruoshi Yuan, Suzhan Zhang, Jiekai Yu, Yanqin Huang, Demin Lu, Runtan Cheng, Sui Huang, Ping Ao, Shu Zheng, Leroy Hood, Xiaomei Zhu

**Affiliations:** 1 Key Laboratory of Systems Biomedicine, Ministry of Education, Shanghai Center for Systems Biomedicine, Shanghai Jiao Tong University, Shanghai 200240, People's Republic of China; 2 Key Laboratory of Cancer Prevention and Intervention, Chinese Ministry of Education, Key Laboratory of Molecular Biology in Medical Sciences, Hangzhou, Zhejiang Province 310009, People's Republic of China; 3 Second Affiliated Hospital, School of Medicine, Zhejiang University, Hangzhou 310009, People's Republic of China; 4 Institute for Systems Biology, 401 Terry Ave. N., Seattle, WA 98109-5234, USA; 5 Shanghai Center of Quantitative Life Sciences, Shanghai University, Shanghai 200444, People's Republic of China

**Keywords:** colorectal cancer, systems biology, robust dynamical states, endogenous molecular–cellular network, stochastic nonlinear dynamics

## Abstract

Colorectal cancer (CRC) has complex pathological features that defy the linear-additive reasoning prevailing in current biomedicine studies. In pursuing a mechanistic understanding behind such complexity, we constructed a core molecular–cellular interaction network underlying CRC and investigated its nonlinear dynamical properties. The hypothesis and modelling method has been developed previously and tested in various cancer studies. The network dynamics reveal a landscape of several attractive basins corresponding to both normal intestinal phenotype and robust tumour subtypes, identified by their different molecular signatures. Comparison between the modelling results and gene expression profiles from patients collected at the second affiliated hospital of Zhejiang University is presented as validation. The numerical ‘driving’ experiment suggests that CRC pathogenesis may depend on pathways involved in gastrointestinal track development and molecules associated with mesenchymal lineage differentiation, such as Stat5, BMP, retinoic acid signalling pathways, Runx and Hox transcription families. We show that the multi-faceted response to immune stimulation and therapies, as well as different carcinogenesis and metastasis routes, can be straightforwardly understood and analysed under such a framework.

## 1. Introduction

Colorectal cancer (CRC) is a leading cause of cancer deaths in the USA [1] and around the world [2,3]. The current research on combined molecular-targeting agents [4], the need for better risk models to incorporate genetic, lifestyle and environmental effects [5], and the development for early detection method [6] all require a better understanding at the molecular level. Ideally, a causal and quantitative model may be used as a ‘dry-experiment’ platform to recapitulate carcinogenesis and metastasis routes, and to test efficacy of drug combinations. Towards such a goal, we investigated CRC under the framework that cancer is a robust state(s) evolutionarily formed from the underpinning endogenous molecular–cellular interaction network [7,8]. The method has been developed and tested in recent years [9–12]. The model construction and analysis are presented in this work. We show that the model indeed captures the essential features of CRC complexity. The nonlinearity of the interaction network dynamics is directly responsible for the formation of the robust CRC subtypes and their differential responses to intervention.

CRC is a clinically heterogeneous disease. About 40% of patients with CRC develop liver metastasis at the time of presentation, with approximately 20% presenting as synchronous metastasis and the remaining 20% as metachronous metastasis [13–15]. Classification schemes are researched to associate clinical outcome with both genetic and phenotypic biomarkers, such as KRAS/BRAF mutations [16], overall mutation frequency [17], mesenchymal character [18], methylation [19] and gene expression profiles [20]. For example, patients who differ in microsatellite instability, which accounts for 7–20% of all patients, appear to have a different prognosis and benefit from chemotherapy [18]. However, these tumour classification schemes have intrinsic difficulties in themselves, such as the choice and comparison across and within different patient sets used in association studies [21]. In addition, the classification schemes do not directly translate molecular heterogeneity of CRC into the disease progression and response to therapies mechanistically. A better understanding for CRC heterogeneity is still needed. Nevertheless, a remarkable finding of a possible robust classification system, by analysing six independent classification systems and their convergence into four consensus molecular subtypes [22], suggests that CRC may have intrinsic robust subtypes regardless of approaching angles. This observation of robust molecular subtypes is consistent with our hypothesis that cancer corresponds to intrinsic robust states formed by an endogenous molecular–cellular interaction network [7].

The hypothesis of an endogenous molecular–cellular interaction network for cancer has been tested in hepatocellular [9], prostate [10] and gastric cancers [11], and acute promyelocytic leukaemia [12]. The major assumption is that the collective effects of interactions among molecular/cellular agents lead to robust states including normal physiological states and cancer subtypes. Carcinogenesis may be understood as a transition from a normal state to cancer state(s). The effect of mutations is naturally incorporated [23]. The idea of collective emergence in biology goes back to Waddington and Delbruck, followed by Monod and Jacob, Hinshelwood and Kauffman [24–29]. High-dimensional attractor states in gene expression patterns and their functional robustness have meanwhile been experimentally demonstrated [30,31].

Genetic and epigenetic studies have identified dozens of genes and molecular pathways, which could possibly have causal relation to CRC. Large-scale molecular profiling, on the other hand, has identified thousands of molecules differentially expressed in CRC and its progression [32,33]. As constructing a network that includes all the genes known to be differentially expressed in CRC and their regulatory molecules is not practical and may not be necessary to provide a picture for the mechanism, we focused on the ones which may be casual to CRC pathology. In addition to well-known molecules and pathways in carcinogenesis, such as p53, Myc, Wnt and NF-κB [34–36], we included molecules important in embryonic development, notably of the gastrointestinal (GI) track and mesenchymal lineage. The feedbacks from intra- and extracellular signalling as well as other molecular interactions are collected from molecular biology and biochemical low-throughput experiments designed to be valid under normal physiological conditions.

The network dynamics simulation reveals the robust states, their transition paths and the associated drives by the orchestrated activities of key regulatory molecules. The calculated robust heterogeneity is validated by the reported phenotypes in the literature and microarray experiments. The implication of such a roadmap in carcinogenesis and metastasis potential is considered in the Discussion section. The intrinsic landscape of CRC may also shed new light on the debate about the stimulating/inhibitory effect of immune response to cancer. Future clinical and biological studies are needed to fully validate these predictions and explore for therapeutic purposes.

## 2. Results

### 2.1. Molecular–cellular network model for colorectal cancer

**Figure 1.**
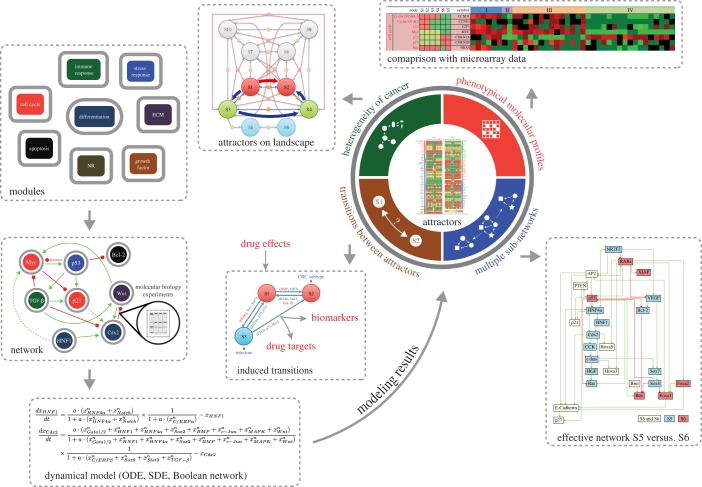
Schematics of endogenous molecular–cellular network construction and modelling. We started with a minimal core network representing regulation of basic cellular functions, such as cell cycle, apoptosis and stress response, similar to previous cancer models [9–12]. Molecules and molecular pathways specific for GI track development and functions, such as transcription factors Cdx2, HNF1, glucocorticoids signalling pathways, were added to the minimal core network. The molecular interactions were collected from the literature, with priority given to those verified by molecular biology experiments. Feedback loops related to inflammation and hematopoiesis were also included. Dynamical system equations (described in electronic supplementary material) were used to compute the attractor states generated by the defined network structure, as well as saddle points for spontaneous transitions between attractors. Random parameter tests were performed to demonstrate robustness of the obtained results. Comparison of gene activity profiles predicted by the attractors with microarray data validated the modelling. Specifically, CRC subtypes as well as normal intestinal phenotype corresponded to the attractors of network dynamics.

The molecular–cellular network we constructed is aimed to be a consolidated and simplified core network for studying CRC development (figure 1). Some of the nodes in the network represent pathways, such as Notch, SHH and Wnt signalling pathways. Cascading effects within these pathways are included in the same manner. Similarly, EGFR, VEGF represent pathway activities, not the expression level of these proteins. Growth factor feedback loops are assumed to be self-contained within the tumour tissue. For example, although VEGF receptors are primarily expressed on endothelial cells, tumour cells might also express them [37]. Production of cytokines is not assumed to be from tumour cells but from the tumour tissue, and emphasis in defining interactions is on receptors/ligands and crosstalk between signalling pathways. Importantly, the model involves cell–cell interactions because of the feedback from cytokines and extracellular matrix. To keep the model simple, only representative molecules of given functional modules are included. For example, for cell cycle regulation, only Cyclin E and Cyclin D are included. The nodes and edges of the CRC network that represent molecules, pathways and their interactions are listed in electronic supplementary material, table S1. Cell cycle, apoptosis, growth factor signalling, differentiation, immune response, stress response, angiogenesis and metastasis are incorporated in the model. The interactions among molecules are obtained from the literature, with preference given to interactions with solid biochemical basis. The constructed network is presented in electronic supplementary material, table S1, with detailed references in electronic supplementary material, table S2.

The network construction is hypothesis-driven in several respects. First, a number of important molecules and pathways identified in previous genetic and molecular biological studies (such as APC and KRAS), with a causal relationship to CRC development, are included as nodes in the network. Second, we assumed that there are intrinsic molecular feedback loops of these genes or molecules. We looked into developmental programmes for these feedback loops. There are studies showing that overactive pathways can lead to pathological conditions. For example, the Wnt pathway, which is active in the developmental process, induced a programme of intestinal genes in the developing lung [38]. Third, we hypothesized that feedback loops mediated by immune response also might have a developmental link in haematopoiesis. Development of leucocyte is orchestrated by the comprehensive regulation of inflammation-related molecular programmes. In addition, physiological inflammation in response to external stimulation dissolves after withdrawal of stimuli, while the developmental programme presumably requires self-sustained feedbacks to quarantine robustness. More details can be found in the electronic supplementary material.

### 2.2. From network model to phenotypes: robust states

**Figure 2.**
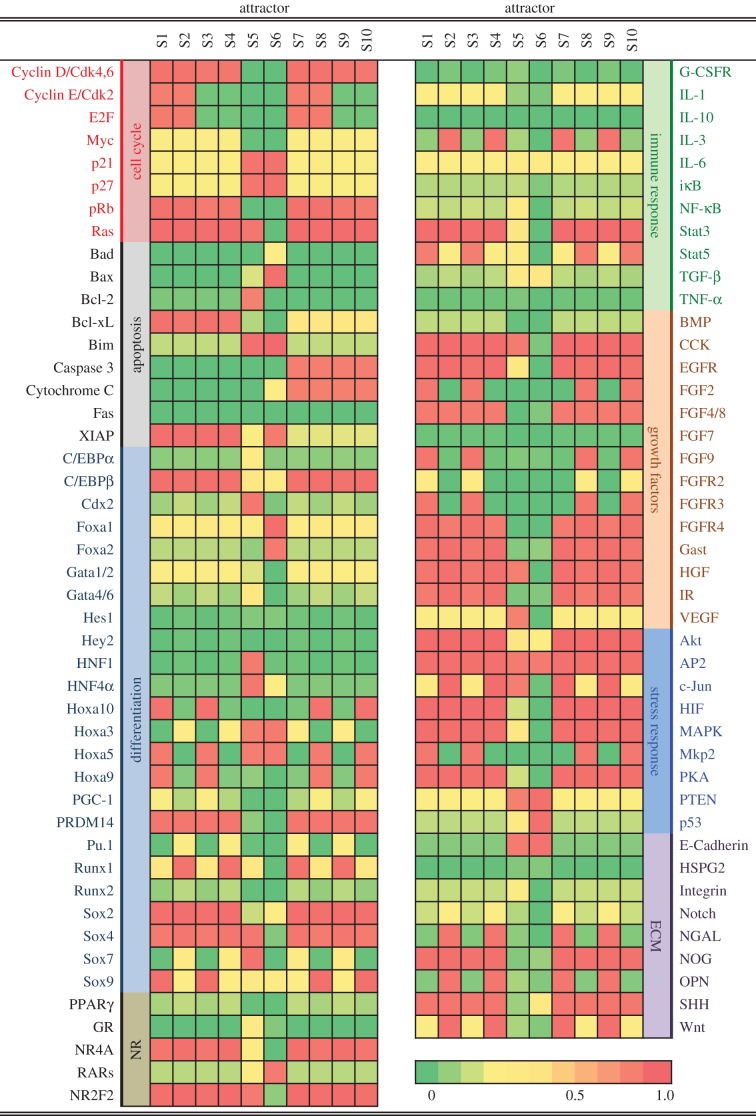
The molecular profiles of the attractors in the dynamical system model of the CRC network (see also electronic supplementary material, table S1). The corresponding equations are listed in electronic supplementary material. Note that attractors S1 and S2, which correspond to proliferating, differ mainly in Stat5 activities. This difference may influence metastasis. Attractors S3 and S4 correspond to non-proliferating states but are otherwise similar to S1 and S2. S5 represents the normal intestine phenotype. S6 resembles a differentiated phenotype with a secretory signature. S7–S10 map into apoptotic states.

In a molecular network, due to the interactions in the network that include feedbacks only for a limited number of combinations, these interactions are ‘balanced’, that is, do not exert any driving force to change the network state. Mathematically, these ‘balanced states’ correspond to fixed-point solutions of a nonlinear dynamical system [39]. If a fixed point is also stable under perturbation, it is an attractor of the system. These attractors are more likely to occur and have a longer residence time in real biological systems with the presence of noise. Saddle points or other fixed points that are partially (i.e. in a subset of state space directions) stable can serve as passes for stochastic transitions between attractors. The attractors are interpreted as cell phenotypes because of their stability, which affords homeostatic robustness to the specific molecular profile that determines phenotype [9,10,40]. We obtained 10 attractors from the network constructed (figure 2), representing the predicted phenotypes. These attractors are robust over a large range of parameters. Among them, attractors S7–S10 display molecular profiles that resemble apoptotic states. Attractors S1 and S2 are proliferation phenotypes, epitomized by upregulation of E2F, Cyclin D/E. By contrast, attractors S5 and S6 represent differentiated phenotypes. Attractors S3 and S4 appear to encode cell cycle arrested state, but otherwise resemble the proliferation attractors S1 and S2.

**Figure 3.**
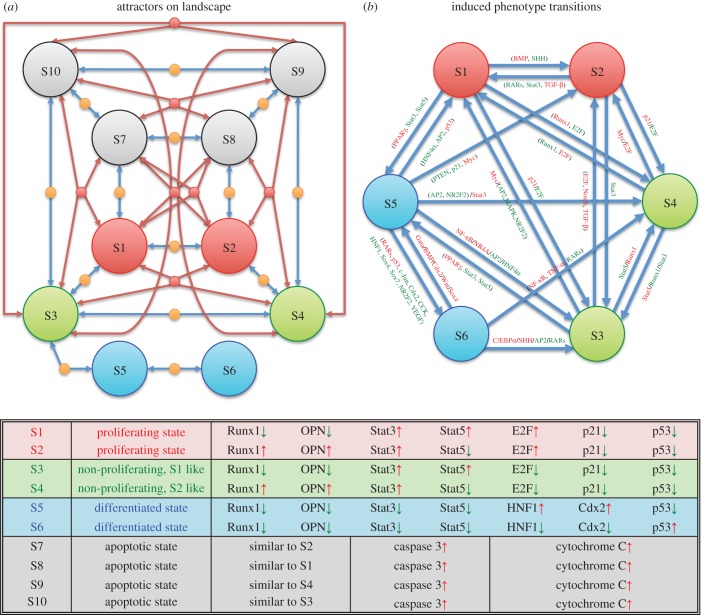
(*a*) Spontaneous transitions between the attractors characterized by saddle/unstable fixed points. In addition to attractors, the dynamical model also contains fixed points of different types, including saddles and other unstable fixed points. These points usually play the role of passes for spontaneous transitions between the attractors. S1–S10 represent the attractors, as shown in figure 2. The saddle/unstable fixed points are denoted by small dots. The flows of cell states from saddle/unstable fixed points to the attractors are represented by arrowed lines. (*b*) Predicted switching between these attractors triggered by induction (perturbation of gene activity). Multiple paths for transitions between any two attractors, representing (tumour) cell type conversions (only selected are shown). Inducers in the same brackets must be operated simultaneously to induce a switch. The slash ‘/’ represents different paths triggered by different inducers. Red/green represents upregulation/downregulation. For clarity, the corresponding phenotypes of these attractors are also listed. Attractor S5 is normal intestine-like, while all the other attractors might contribute to CRC. Since attractors S3 and S4 are similar to S1 and S3, there are essentially three attractors contributing to CRC subtypes: S1, S2 and S6.

Among the two growth states with predicted active cell cycle activity (attractors S1 and S2 in figure 2), one seems to represent growth factor-stimulated proliferation, characterized by active FGF, HGF, IGF, EGF signalling, consistent with epithelia-to-mesenchymal transition and endoderm organ formation (attractor S1 in figure 2) [41], while the other appears more typical of CRC: active Wnt pathway [42], high expression of Runx1 and osteopontin [43]. These two growth states might both contribute to cancer progression because they interconvert into each other relatively easily. The growth-factor-stimulated state (attractor S1) is inducible from attractor S2 by reduction in Stat3 pathway (figure 3). By contrast, the typical CRC-like state (attractor S2) is not inducible from attractor S1 by inflammation alone. Instead, attractor S2 is induced by both inflammatory and immune suppression from attractor S1 by increasing TNF-α and TGF-β. There are different ways to interpret the possible phenotypes represented by attractors S1 and S2, but it is apparent that they have characteristics of both an aberrant developmental process and inflammation.

The differentiated states (attractor S5) represent an intestine-like phenotype, characterized by activated Cdx2 and HNF1. Both genes are indispensable for intestinal function [44]. Attractor S5 also has higher GATA4/6 level, consistent with an intestinal epithelial cell phenotype [44]. Attractor S6 differs from S5 by being Foxa1,2/SHH/Sox-positive. It might represent stomach or intestinal secretory cell lineage [11,45–47]. For these attractors, the active network nodes (with a high expression level) can be identified, as shown in electronic supplementary material, figure S1. This is a way to visualize the effective subnetwork specific for each attractor. The active nodes for the normal intestine-like state (attractor S5) are compared with those for S1, S2 and S6 separately in electronic supplementary material, figure S1. The network topology indeed suggests that attractors have their own separate positive feedback loops. Also, attractors have mutual suppressing relations, as shown in the graph, although not in the simple form as seem in direct node-to-node switches.

### 2.3. Comparison with observations I: common features of colorectal cancer

CRC is a disease that exhibits inter-individual variation both in pathology and in its response to treatment. The model predicts multiple fixed points. This multi-stability opens the possibility that cells in a tumour occupy distinct attractors, and possibly interconvert between them—giving rise to mixtures of cell phenotypes—which would explain the widely observed non-genetic cellular heterogeneity of tumour tissues [48,49]. However, obviously transcriptome analyses of tumours [50] still can identify subtypes of CRC, perhaps because some attractor dominates. Thus, we first identified common features in CRC profiles derived from modelling and compared them with clinical measurements.

**Figure 4.**
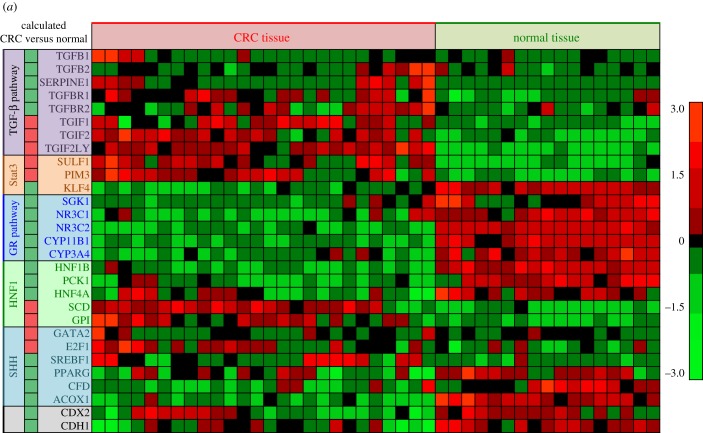

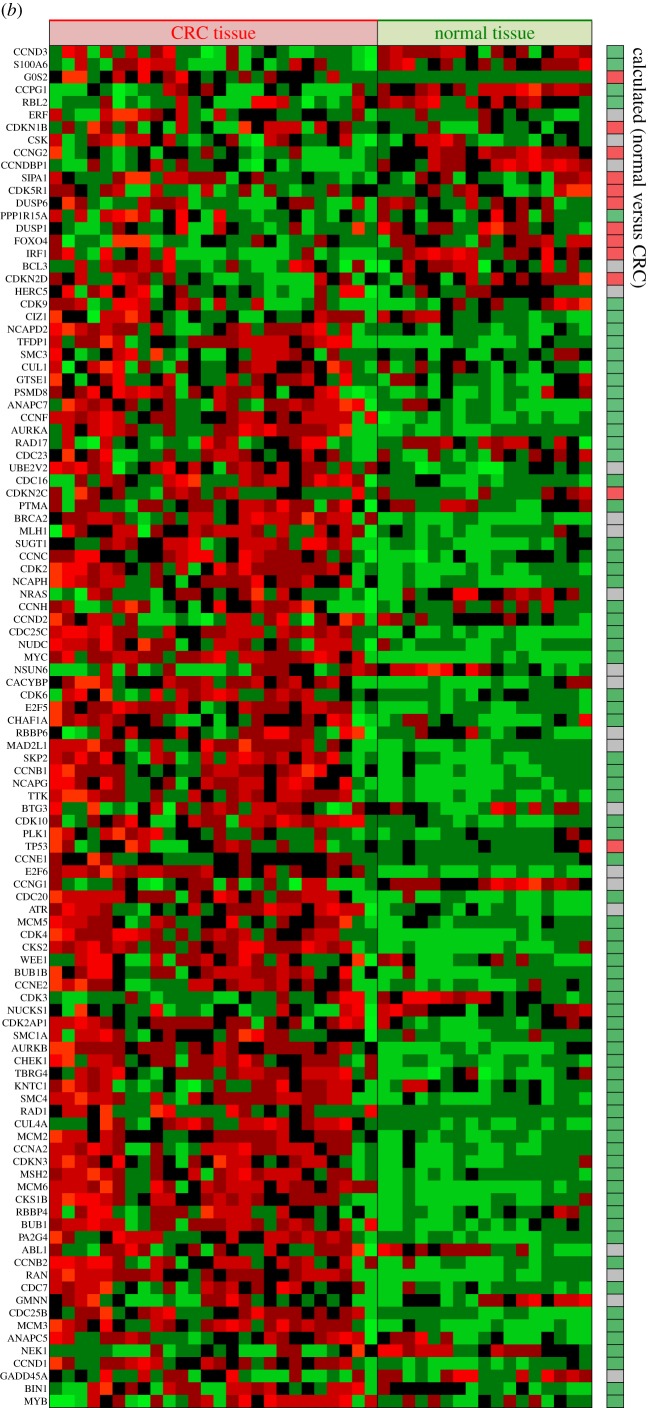

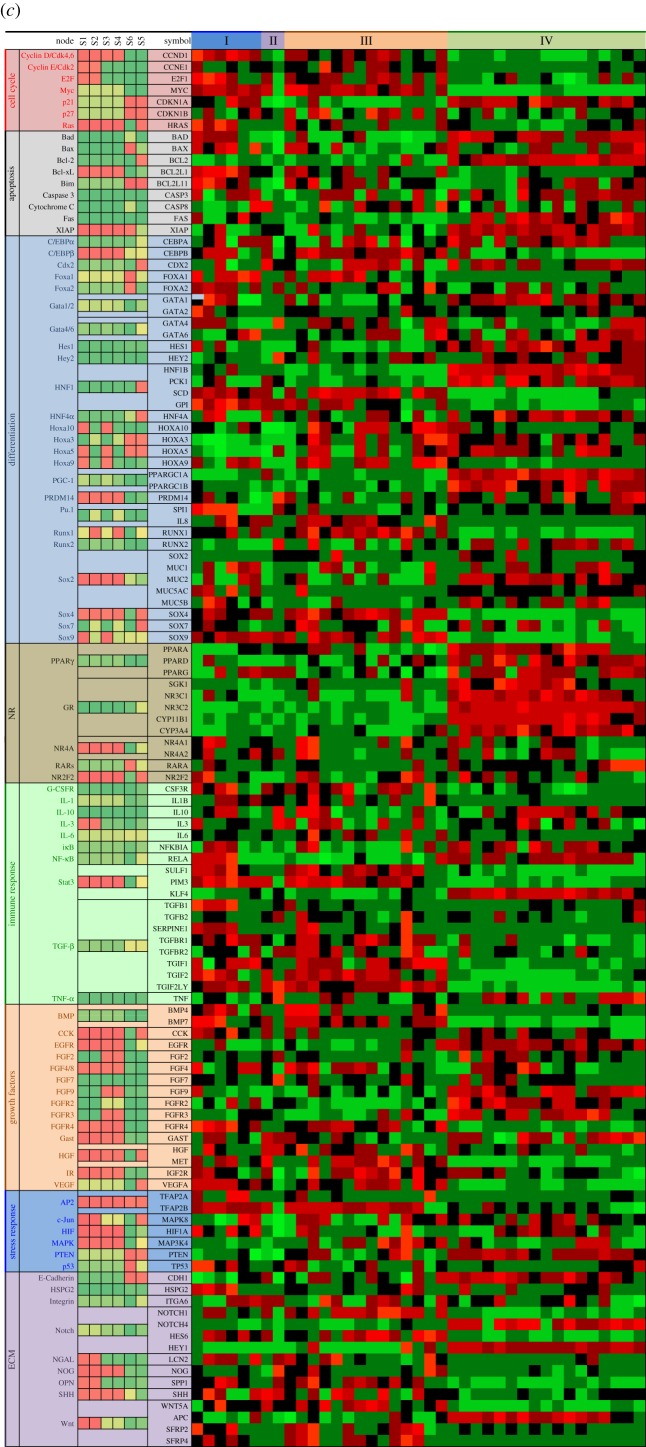
Comparison between computed results and microarray data. The microarray profiles were obtained from the second affiliated hospital of Zhejiang University for a total of 17 normal tissues and 26 CRC tissues of different patients. Every column is the profile of a computed attractor, the profile of relative gene activity between CRC and normal, as indicated, or a microarray profile from CRC tissue. Comparison of the predicted attractors profiles with observed microarray data show common features as listed in (*a*) and (*b*). The cell cycle module is shown in (*b*). The list of genes for (*b*) were obtained from Theilgaard-Mönch *et al.* [104]. (*c*) A broad comparison of the model prediction and observation. Normal tissues are in group IV. Group I are patients showing attractor S6 signature, group II patients showing some normal like attractor S5 signature, and group III patients showing a signature of attractor S1 and S2, but not S6. References used for annotations are [105–110]. A full list of data and references is available in electronic supplementary material, file S1. (Parts (*b*) and (*c*) shown on following pages.)

Attractor S5 (figure 2) represents normal intestinal phenotype. If the intestinal cells were trapped in other attractors, they would be pathological, although not necessarily CRC. We assume that cancer tissue is a heterogeneous combination of attractors S1–S4 and S6, then compare with real tumours. Figure 2 shows that HNF1, HNF4, Cdx2, TGF-β pathway, E-cadherin and glucocorticoid pathway characterize normal intestine. They are unambiguously downregulated in every possible abnormal state in the computed attractors S1–S4 and S6. The microarray profile from patients obtained from the second affiliated hospital of Zhejiang University showed downregulation of HNF1B expression in CRC patients. As expected [51], HNF1B transcript downregulation accompanied a decrease in gluconeogenesis (manifest in PCK1, phosphoenolpyruvate carboxykinase), upregulated glycolysis (GPI, glucose-6-phosphate isomerase) and lipogenesis (SCD, stearoyl-CoA desaturase). Microarray results for Cdx2 and HNF4A were not conclusive. TGF-β signalling impairment is consistent with upregulation of negative regulators TGIFs [52,53]. Glucocorticoid receptor NR3C1 and mineralocorticoid receptor NR3C2 were both downregulated in CRC patients. Glucocorticoids target genes CYP3A4 [54], SGK1 [55] and CYP11B1 (steroid 11-β-hydroxylase), which are required for synthesis of glucocorticoids [56], were downregulated in CRC patients. The model predicts upregulation of Stat3 in attractors S1–S4. Expression of several Stat3 target genes, SULF1, PIM3 and KLF4 [57] was altered accordingly, as shown in figure 4*a*. In addition to transcripts, the kinase activities of the Akt and MAPK pathways were shown to be active in CRC stem cells [58], consistent with both attractors S1 and S2. These experimental results show that network modelling results are consistent with observation. One application for these common features (shown in figure 4*a*,*b*) predicted by models is to use them as molecular signature in prognosis and diagnosis. Unlike signatures defined by brute-force statistical analysis of data [50,59], attractor-informed patterns capture the natural constraints imposed by the underlying regulatory network.

### 2.4. Comparison with observations II: heterogeneous features of colorectal cancer

The computed attractors S1–S4 and S6 (figure 2) together with their combination define a set of subtypes that the network model predicts. There are two proliferating attractors S1 and S2. Stat3 and Stat5 pathways are both active in attractor S1, while only Stat3 is active in attractor S2. Runx1, interleukin signalling, Wnt pathways and NR4A are more active in attractor S2, while Hox genes and FGF signalling are more active in S1 attractor. Attractor S2 also predicts upregulated OPN, which favours metastasis [60]. When comparing patients' mRNA profiles, we can determine which attractor the profile of an individual tumour is closer to. For example, patient 6 (in electronic supplementary material, file 2) is close to attractor S2, with high OPN, Runx1 and low Hoxa10. Compared with patient 6, patient 9 is closer to attractor S1, with low OPN, Runx1 and high Hoxa10. Attractor S6 resembles a differentiated state with possible secretory signature. There are reports of mucinous CRCs [61–63], which express Sox2, MUC5AC, MUC2, MUC5B and p21 (CDKN1A). In the microarray data (in electronic supplementary material, file 2), several tumours (of patients 2, 3, 4, 5, 7, 15) exhibit a molecular profile resembling that of S6, characterized by upregulated p53 or p27 and upregulated mucins. However, there is a difference between attractor S6 and these patients' profiles, in that the apparent Stat3 activity is high in these patients, as suggested by elevated SULF1, PIM3 and KLF4. It is now well documented that most (if not all) tumours, including CRC, contain multiple cell types, even among the isogenic cells [58]. Such phenotypic heterogeneity is best explained by multi-stability [48,49]. One possibility is that these patients’ tumours may contain cell types other than S6 that would map into attractor S1 or S2. These p53- or p27-positive patients are particularly interesting because they have a worse clinical outcome.

### 2.5. Transition between robust states

Mathematically, in addition to attractors, the network dynamics also predict saddles and other unstable fixed points. However, these states are not locally stable. When perturbed, they will flow to different attractors, driven by molecular interactions [9,10,64,65]. The saddle points have straightforward dynamical interpretation as the point of passage of the ‘least effort’ path for spontaneous transitions between attractors driven by noise [66–68]. Such paths are shown in figure 3*a*. Attractors S1–S4 are connected through multiple saddles and other unstable fixed points to each other. Therefore, it is likely that cells interconvert due to external perturbations or internal fluctuations. The exception is attractor S6, which is not connected to proliferating states represented by S1 and S2. As S6 resembles a non-proliferative and differentiated state, it has to coexist with either S1 or S2 to allow for tumour growth. This heterogeneity of growth behaviour fits the biological framework of cancer stem cells that drive tumour growth. Among the possible CRC attractors S1–S4, attractor S3 is connected by a saddle point to the normal intestine-like attractor S5. This would suggest the possibility that carcinogenesis could be initiated by a transition from the normal intestine-cell-type attractor S5–S3 through the saddle point.

Transition from one attractor to another in the model can also be enforced by resetting the values of a few selected nodes of the network, which in reality maps into upregulating or downregulating of the corresponding molecules by external induction. Starting from normal intestine-like attractor S5, if Myc is upregulated, and p21 and PTEN downregulated simultaneously, this would cause a switch to the proliferative state S2, with typical CRC molecular profile. In the model, when NF-κB is numerically upregulated in the normal intestine-like attractor S5, the cell will switch to attractor S3, consistent with the notion of inflammation as promoter of carcinogenesis. A switch from attractor S3–S1 is caused by activation of Myc. Computed trajectories for selected attractor switching are shown in figure 3*b*. Consistent with the modelling results that attractor S6 is not as closely connected through saddles or other unstable fixed points to the rest of the attractors, inducing S6 from S5 is more difficult.

### 2.6. Validation of the modelling results

We use standard hypothesis testing for the validation.

Comparison between modelling results and clinical data:

*Vector* (a). Extracting results from clinical data. We use the average gene expression value of the microarray data from CRC (26 samples) versus the average value of those from normal tissue (17 samples) to generate a *vector* (a column matrix) with 1 and −1 elements. The rule is listed below: if the expression level of a gene from the CRC data over that from the normal tissue is larger than 1 plus a threshold value, we consider this gene is upregulated in CRC and vice versa. In case the upregulation or downregulation is not determined by data, we remove the gene from the list, because we cannot distinguish the two possibilities that (i) the upregulation or downregulation of the gene is not shown due to inaccurate measurements, and (ii) the gene expression level is similar in CRC and normal tissue.
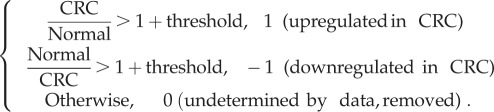


**Figure 5.**
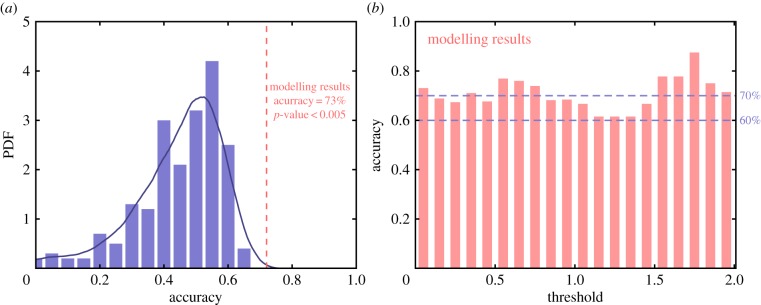
Validation of the modelling results through comparison with randomly rewiring networks. (*a*) Distribution of consistency with clinical data for a group of 200 randomly rewiring networks. Our model has 73% accuracy, which is significantly larger than randomly rewiring networks with *p* < 0.005. (*b*) The influence of the threshold parameter in the comparison with the clinical data is not significant. The details of the comparison are provided in the electronic supplementary material, file S3.

*Vector* (b). The *vector* (with −1, 1, 0 elements) for the modelling results can be similarly generated without removing zeros, because zeros mean that the modelling results predict a similar gene expression level. We use the same *threshold* for the two *vectors* (a) and (b). We could then compare the two *vectors* directly and compute an accuracy rate for the consistency (see electronic supplementary material, file 3 for details). For a 5% threshold value, the accuracy is about 73% with a total of 78 genes, which is close to the accuracy of microarray data based on our previous experience [8,10–12,23]. We also compute the accuracy with a varying threshold value in figure 5*b*. It demonstrates the robustness of our results.

*Test 1: Comparison with random guess.* We generate 1 million random vectors with uniformly distributed 1 and −1. For better matching with the data, we do not include zeros here. The distribution of the accuracy is shown in electronic supplementary material, figure S3. The test shows that our modelling results have *p* < 0.00001.

*Test 2: Comparison with randomly rewiring networks.* The null hypothesis is that the accuracy of the results from the CRC network model versus clinical data is not significant compared with results generated from randomly rewiring networks.

For randomly rewiring networks that have the same total number of interactions but are randomly rewired the connections and interaction types (inhibition or activation), we compute the accuracy rate distribution shown in figure 5*a*. For each randomly rewiring network, we compute its dynamical stable states and the optimal two-state combination (assigned to cancer and normal separately) compared with the data. The estimated distribution from 200 networks shows that the *p*-value is less than 0.005, such that the null hypothesis should be rejected.

## 3. Discussion

### 3.1. Clinical implications: colorectal cancer carcinogenesis, metastasis and treatment

CRC development may take years and involve multiple risk factors [3,69–71]. Intermediate steps have been observed. The molecular network constructed here exhibits both normal intestine-like attractors and proliferating CRC-like attractors. The predicted possibility for switching from normal intestinal to CRC states may be relevant to carcinogenesis summarized in figure 3. The results are consistent with the heterogeneous nature of carcinogenesis and the role of inflammation and stress [72,73]. The network model naturally allows for a multitude of paths for the transition to the cancerous attractors [74], many of which are associated with an inflammatory programme. Specifically, the model predicts that tumour attractors can be reached through changes in Myc, p21 or PTEN, or activation of NF-κB as intermediate steps. In addition, increased stress and p53, in combination with loss of retinoic inducible transcription factor AP2 and nuclear factor HNF4, may also lead to switching to CRC, from normal intestine-like attractor S5–S1.

The network supports several attractors that might associate with distinct phenotypes; in principle, how they contribute to metastasis can be discussed. For instance, the proliferating attractors S1 and S2 differ in Stat5 pathway activities. Stat5 signalling has been shown to have an independent effect on cell morphology via regulation on the expression of adhesion molecules [75,76]. Impaired Stat5 signalling leads to non-healing wounds in the intestine [76]. The loss of intestinal barrier due to Stat5 dysfunction might contribute to metastasis in the phenotypes given by the network. Another metastasis-related protein, PRL-3, is a downstream target of Stat5 and Stat3 [77,78]. Note that all the network-supported phenotypes of CRC have metastasis potential. The Runx1 and OPN high/low phenotype might favour metastasis through loss of intestinal barrier/activate PRL-3.

Transition from CRC to normal intestine state, on the other hand, might be relevant to the prevention and treatment of CRC. There are three major types of CRC, represented by the type with positive p53 or p27 (S6), and the OPN- and Runx1-positive (S2/S4) and-negative (S1/S3) types. For the OPN- and Runx1-negative phenotype, switching to the normal intestinal phenotype requires a suppression of the inflammation programme, which could be achieved through a transition from S3 to S5 by reducing Stat3 and Stat5. For the OPN- and Runx1-positive phenotype, switching to the normal intestinal phenotype would require first a switching to the OPN- and Runx1-negative phenotype, from S2 to S1, through suppression of RARs signalling and simultaneous anti-inflammation (reducing Stat3, increasing TGF-β signalling). The p53/p27-positive CRC type could be converted to the OPN- and Runx1-positive phenotype via S4, by promoting inflammation (NF-κB and TNF-α) and suppressing retinoic acid signalling RARs.

The theoretical base under immunotherapies is the cancer immunosurveillance hypothesis [79]. However, experimental evidence leads to various conclusions, ranging from that the immune system naturally protects against cancer [80] to that the immune reaction is almost always stimulatory to the tumour's growth [81]. The current focus on the differential responses to immunotherapy is usually about the state of the immune system, such as the T cell population and types [82,83]. Our results show that different intrinsic robust cancer subtypes may respond to immune cells, such as T cells and macrophages, differently (figure 3*b*). Since the focus of the model is on tumour cells, the calculated different responses are due to the network-wide regulation over NF-κB and Stat3/5 inside tumour cells. If the internal status of a tumour does not favour NF-κB activation, the remaining effect of cytokines is left as a growth factor. Therefore, the calculated likely routes for transitions between normal and cancer states, corresponding to the genesis and development of cancer and transition among subtypes, have non-identical dependence on immune activation.

The network for CRC presented in this work is essentially a core decision-making network for intestinal development and function. Signal transduction and transcriptional regulation are two main molecular interaction types included. There are no particular cancer-specific genes or cancer pathways in the network. As consequences of the network wiring diagram, its dynamics generate multiple attractors, which are robust states and correspond to both normal intestinal phenotype and CRC subtypes. For each of the robust state, a subnetwork of the active nodes thus represents ‘cancer network’ or ‘normal network’ separately, although they are part of the same endogenous network. Interestingly, the nodes active in the normal and CRC-like attractors have mutual inhibiting effects. The concept of multiple attractors emanating from a single network naturally suggests that healthy and abnormal cell states are ‘alternative sides of the same coin’—a feature not manifest in the traditional notion of specific linear pathways implicated in individual ‘hallmarks’ of cancer [84,85]. Here, we tested the cancer attractor concept, which has a solid foundation in a formal framework, but typically has been articulated in generic terms. We link it for the first time to specific biological observations, both in terms of the network topology (by modelling experimentally tested molecular interactions) and network dynamics (by comparing measured transcriptomes with predicted attractors). We show high consistency in that our network model predicted features of CRC including its diversity among individual patients.

## 4. Material and methods

### 4.1. Network construction

The construction of the network followed an incremental procedure aimed at capturing the essential features of CRC while keeping the network to the core level. To start with, we first built a minimal cellular model, with cell cycle, apoptosis, growth factor signalling, immune response and stress response modules, similar to previous works [9–12]. Molecules and molecular pathways well established in cancer, such as PTEN, p53, Myc, Ras and NF-κB, were in this minimal model. Then we added several additional modules specific to CRC. First, Cdx2 [86] and Notch signalling pathway [87] were included for their role in intestinal differentiation. Cdx2, HNF1 and Gata family were added for their indispensable roles for intestinal function [44]. Wnt [88,89], SHH and BMP signalling pathways [90–92] were also included for mesoderm development. Wnt signalling is active in posterior endoderm in early mouse development. Wnt induces Cdx2, a cell fate switch of intestinal differentiation [93]. Glucocorticoid pathway was included for its effect on GI mucosa [94] and PPARγ for inhibiting intestinal inflammation [95,96]. We explored inflammation beyond NF-κB pathway, into haematopoiesis because of many shared transcription factors in CRC and leukaemia. One possible feedback link is Runx1, which is often over-expressed in CRC. Runx1 is both a gastric gene and indispensable in haematopoiesis, and associates with the myeloid leukaemia [97–99]. A number of leukaemia-related genes and molecular signalling pathways (Hox family, Pu.1, IL3) were also added for their possible role in immune response. Finally, angiogenesis and metastasis modules were included in the model.

### 4.2. Network modelling

The dynamical model for the CRC network was constructed based on a framework of the endogenous molecular–cellular network proposed previously [7,8]. Endogenous network models have been built for hepatocellular [9], prostate [10] and gastric cancers [11], as well as acute promyelocytic leukaemia [12]. As detailed knowledge about the interactions between the network nodes (e.g. *in vivo* parameters) is usually absent, a coarse-grained modelling of the network dynamics was employed. The interactions between the network nodes (agents) were described by activation/inhibition using differential equations [9–12], as a refinement of coarser descriptions such as the threshold Boolean network [100,101].

The dynamical equations for the concentration/activity of an agent *x_i_* under the influence of other agents 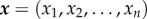 take the following form:
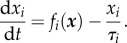


This expression consists of a generation term and a degradation term, which is consistent with the chemical rate equation. The degradation time is set to be unity for all agents (i.e. *τ_i_* = 1). The underlying assumption is that the baseline decay times for the agents are of the same order of magnitude. Although protein degradation time may vary significantly, most of the agents in the network for the core endogenous interactions operate at the regulatory level relevant to cell proliferation and death. It has been shown that the lifetimes of more than 4000 proteins in a non-dividing mammalian cell are narrowly distributed, with a median value of 35.5 h [102]. No structural proteins with a long lifetime are included in the model. For the signal transduction processes, the signalling pathways are treated as a whole until downstream transcription takes effect, avoiding the possible short response time of the initial stages. The active regulations of protein degradation, such as the regulation of PTEN stability, are included as activation/inhibition in the network [103].

Here, we use a nonlinear Hill function to implement the coarse-grained activation and inhibition. For example (see electronic supplementary material, table S1), HNF1 is activated by HNF4 α and Notch, and inhibited by C/EBP α; the equation has the form
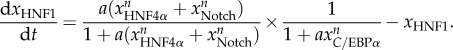


In general, if an agent *x* is activated by agents 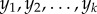 and inhibited by agents 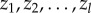, we have
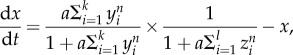
where two parameters *n* and *a* can be tuned in the equation. Note that our differential equation modelling results are consistent with those from the parameter-free Boolean dynamics (see electronic supplementary material, table S6), such that the model is intrinsically not sensitive to parameters. Our large-scale random parameter tests also validate this point (see electronic supplementary material, figure S2).

The value for each agent is normalized from zero to one. The dynamics have a conservation property: they will keep the value of each agent normalized (between zero and one). A full list of 89 equations can be viewed in the electronic supplementary material. The equations can generate several local attractors with biological significance. In this CRC model, we obtained 10 attractors, as well as 14 saddle points and seven other unstable fixed points (shown in electronic supplementary material, table S3 and table S7 separately). A series of random searches crossing several magnitudes in the number of random initial points are executed. It turns out that no new attractors can be found, which means the results may cover the major attractors that are biologically stable (with a relatively large attractive basin) in the state space.

To validate the modelling results: (i) we use both Newton's iteration method and Euler's method separately to obtain attractors from the differential equations (electronic supplementary material, table S3); (ii) we made a series of random parameter tests (see electronic supplementary material, figure S2) that allow independent random Hill coefficient *n* and prefactor *a* for each interaction (total of 525 interactions), and the obtained results are almost invariant; (iii) we used alternative forms of equations and the attractors are reproduced (electronic supplementary material, tables S4 and S5); and (iv) we computed the Boolean network model and found the result supports nine of the 10 attractors obtained from the differential equations (see electronic supplementary material, table S6).

### 4.3. Sample collection and microarray experiments

Colorectal tumour and normal tissue specimens from 26 CRC patients with clinical outcome and three healthy individuals were collected from the second affiliated hospital of Zhejiang University. All samples were concurrently analysed using Affymetrix Human Genome U133 Plus 2.0 microarrays. The extraction of RNA was performed following standard protocols provided by the manufacturers.

### 4.4. Microarray data analyses

For gene expression analysis, tumour and adjacent normal tissues were investigated using an Affymetrix Human Genome U133 Plus 2.0 microarray. Data were acquired by GeneChip operating software v. 1.4. After quality checks, raw intensity data were processed by quantile normalization with robust multi-array average to remove systematic bias using Affymetrix Expression Console v. 1.12. The complete table of microarray data with recognized gene symbol and function is provided in electronic supplementary material, file S2.

## Supplementary Material

Supplementary Materials

## Supplementary Material

Supporting file 1

## Supplementary Material

Supporting file 2

## Supplementary Material

Supporting file 3

## Data accessibility

All data of this work are available in the electronic supplementary material.
