# Supplementary material for: Beyond cancer genes: colorectal cancer as robust intrinsic states formed by molecular interactions
Source: Open Biol. 2017 Nov 8;7(11):170169. doi: 10.1098/rsob.170169 (PMC5717345; doi:10.1098/rsob.170169)
Supplement: Supplementary Materials [file rsob170169supp1.pdf]

Electronic supplementary material for "Beyond cancer genes: colorectal cancer as robust intrinsic states formed by molecular interactions"

Ruoshi Yuan, Suzhan Zhang, Jiekai Yu, Yanqin Huang, Demin Lu, Runtan Cheng, Sui Huang, Ping Ao, Shu Zheng, Leroy Hood, and Xiaomei Zhu

(Dated: October 22, 2017)

CONTENTS

I. Network construction 3

A. Molecular pathways underlying genetic risks 3

B. Identify molecules and pathways from gastrointestinal track development 3

C. Immune response and its possible active role in CRC 5

D. Key nuclear receptors in GI development and inflammation 5

Table S1: Molecular interactions in the CRC endogenous network 7

References 8

F. Network definition and reference for molecular interactions 10

Table S2: Molecular interactions in the CRC network with references 10

References 14

II. Network modeling 39

A. Modeling the network dynamics by mathematical equations 39

1. A full list of equations for the network dynamics 39

B. Robustness of the modeling results 43

1. Random parameter tests 43

2. Robustness tests by using alternative forms of equations 43

3. Comparison with Boolean dynamics 44

C. Transitions between attractors 45

References 45

III. More supplementary figures and tables 46

Figure S1: Effective sub-networks between attractors 46

Figure S2: Recurrence rate of attractors under random parameters 47

Figure S3: Hypothesis test of modeling results *vs.* random guess 48

Table S3: Molecular profile of computed attractors 49

Table S4 & S5: Recurrence of attractors using different forms of equations 50

Table S6: Boolean dynamics *vs.* differential equations 52

Table S7: Unstable fixed points from differential equations 53

I. NETWORK CONSTRUCTION

A. Molecular pathways underlying genetic risks

Up to one third of CRC are likely related to inheritance. First degree relatives of patients with CRC have a 2- to 4-fold risk of developing neoplasm compared with the general population [1–6]. One example clearly showing the extensiveness of heredity in CRC is the research conducted in Utah [4]. Utah has the lowest incidence of CRC in the United States for both men and women [7]. Since the Church of Jesus Christ of Latter-day Saints, which the majority of its residents belong to, has a health code against the use of coffee, tea, alcohol, and tobacco, presumable risk factors due to diet are excluded to some degree.

Between 2% to 5% of CRC arise with lynch syndrome, adenomatous and hamartomatous polyposis syndromes which demonstrate Mendelian inheritance pattern [6]. The genes responsible for these disorders have been identified and carriers of the pathogenic mutations have a high lifetime risk of cancers. Among the overlapped genes with the ones identified in somatic mutations [8] is adenomatous polyposis coli (APC), which encodes a protein repressing canonical Wnt signaling pathway at several levels. Another ones are involved in TGF-β signaling, such as Smad4 and BMPR1A [6, 8]. Except for APC, KRAS, p53, most of somatic mutations in oncogene and tumor suppressors happen at low frequencies. Possibly, canonical Wnt signaling and TGF-β signaling pathways are the critical ones for CRC development while others work as additional contributors.

The predisposed genetic condition in these studies has implications. The vast majority of the gene variants for CRC, mostly not yet identified, carry out normal physiological functions which are not grossly affected, therefore not critical by themselves. The combined effect of the gene variants can be additive, possibly through interactions. Therefore, it is necessary for us to extend the search for molecules for CRC development beyond current genetic studies. Also, a method to account for the cooperation may be helpful.

B. Identify molecules and pathways from gastrointestinal track development

Canonical Wnt signaling pathway, which is implicated in genetic study of CRC has an important role in controlling patterning and proliferation of gastrointestinal track (GI) at embryonic stage. In the study of familial and sporadic colon cancers, association between

the GI tract and the canonical Wnt pathway was first established [9, 10]. Mutations in the components of this pathway that result in tumor formation are those that leading to stabilization of beta-catenin and hence constitutive activation of the pathway. Mouse models with interruption in various components elucidated role of Wnt pathway in development [10]. For example, disruption of Tcf4 resulted in depletion of the epithelial stem cell compartment in the crypts of the small intestine [11].

We searched for molecules interacting with Wnt pathways in order to include feedbacks into the network. Wnt signaling is active in posterior endoderm in early mouse development and induces Cdx2 [12], a cell fate switch of intestinal specification in the endoderm [13]. Wnt is a transient process and appears to be repressed by Cdx2 after differentiation [13] while activation of Wnt signaling is sufficient enough to induce intestinal differentiation of embryonic stem cell-derived endoderm, specifically inducing posterior small intestine and large intestine fate [11]. The interaction of Cdx2 and Wnt pathway is complex that only 18% of the genes induced by Wnt activation were induced by Cdx2 while larger degree of overlap was observed between genes down-regulated by them, suggesting additional pathways may also play a role.

Endoderm-mesoderm interaction exists throughout GI developmental process [14]. Gut endoderm was able to induce smooth muscle differentiation in non-gut mesoderm, possibly through secreted molecule Sonic Hedgehog (SHH) [15]. SHH signaling has a dual role in proliferation and differentiation, as over-expression of SHH transgenic mice lead to increased cell proliferation of gut mesoderm as well as smooth muscle specification [16]. The endodermal signal, such as SHH, BMP4 allows the epithelial lumen to be surrounded by mesoderm specified to form visceral mesoderm [14]. Similar to Wnt pathway, hedgehog pathway was also found in cancer progress by induction of epithelial-mesenchymal transition. Through the expression of the transcription factor Gli1, hedgehog ultimately activates Snail and drives cancer invasion [17]. Another important signaling pathway in GI development is Notch. Notch regulates adult intestinal development by controlling the balance between secretory and absorptive cell types [18]. Notch activation in the embryonic foregut results in reversible defects in villus morphogenesis and loss of the proliferative progenitor compartment [18]. SNotch signaling pathway is also proposed to play an important role in cancer invasion [19]. These important pathways for developmental process and cancer also interact. For example, Notch may directly suppress Hedgehog via Hes1 mediated inhibition

of Gli1 transcription [20]. In addition to Wnt, SHH, Notch, which are important for developmental process, we include other molecules for GI development, such as those of GATA, Hox family.

C. Immune response and its possible active role in CRC

CRC is a type of cancer which demonstrates apparent association with inflammation [21, 22]. Both ulcerative colitis and Crohns colitis are at increased risk of developing CRC [22]. Colitis-associated colorectal cancer affects individuals at a younger age than the general population and the risk for developing CRC increases with longer duration of colitis. Inflammation is known to induce cellular stress as well as to modify microenvironment. In the context of network, we found indications that in the case of CRC, inflammation might not entirely be a passive response to stress and irritation, but possibly form an aberrant intracellular feedback. In adult human, Cdx2 is only detected in intestinal epithelial cells [23]. It is aberrantly expressed in most cases of acute myeloid leukemia and promotes leukemogenesis [24], and associated with Hox dysregulation expressions [24]. Another possible feedback link to inflammation in CRC is frequently observed Runx1 expression [25, 26]. Runx1 is a gastric [27] gene which also indispensable in hematopoiesis and associates with the myeloid leukemia [28]. In fact, aberrant developmental process is shown to be involved in carcinogenesis through epithelial-mesenchymal transition [17, 19]. We found no fundamental reason that aberrant developmental process in cancer is limited to epithelial-mesenchymal transition not that of leukemogenesis. The activation of pathways in leukemogenesis might lead to additional contribution to inflammation through receptors and cytokines usually observed in leukocytes. We included molecules and pathways known to interact with molecules such as Cdx2 and Hox family in acute myeloid leukemia in the network for necessary feedbacks to molecules of interest.

D. Key nuclear receptors in GI development and inflammation

Normal intestinal function also requires a number of nuclear receptors. Their expression and activities may also be altered by inflammation [22, 29]. PPARγ ligands have been shown to inhibit intestinal inflammation [30, 31]. Glucocorticoids affect development of the

gastrointestinal mucosa in addition to their anti-inflammation function [32]. Given the importance of glucocorticoids in GI track, the repressors of glucocorticoid pathway are also of interests. The NR4A subgroup of nuclear receptors might be considered early response genes which are induced by a large set of stimuli, such as growth factors, fatty acids, cytokines [33]. NR4As have anti-inflammation role through induction of iκB [34]. In addition, NR4As are also antagonists of glucocorticoids pathway [35], which is also anti-inflammatory. Thus inflammation stimuli NR4As and glucocorticoids form a complex relationship. There are at least two possible relations of NR4As to CRC. First, activation of NR4As favors angiogenesis [36]. Second, NR4As might interfere with nuclear signaling through competing with binding partners, such as VDR and RXR [33, 37]. Unlike the extensive research on signaling pathways in developmental processes such as Wnt, SHH and Notch, research on nuclear receptor signaling pathways in CRC is less mature. In the network model, we included only the ones which seem to be most important.

Table S1. Molecular interactions in the CRC endogenous network. Molecules and pathways are grouped according to their roles in cell cycle, apoptosis, differentiation, nuclear receptor signaling, immune response, growth factors, stress response, and interaction with extracellular matrix. NR: nuclear receptor signaling. ECM: molecules and pathways interact with extracellular matrix. Detailed description and references are shown in Table S2.

Category	Nodes	Activator	Inhibitor
Cell Cycle	Cyclin D/Cdk4,6	E2F; EGFR; Pu.1; Myc	p21; p53
Cell Cycle	Cyclin E/Cdk2	E2F; Myc	p21; p27; PTEN
Cell Cycle	E2F	E2F; Myc	p21
Cell Cycle	Myc	E2F; MAPK; Notch; pRb; SHH; Wnt	AP2; p53; TGF-β
Cell Cycle	p21	AP2; Gata1/2; HNF4α; p53; Runx1; TGF-β; TNF-α; GR	Akt; Hes1; Myc
Cell Cycle	p27	E-Cadherin; Gata1/2; PTEN; TGF-β; NR4A	Akt; Hes1; Myc
Cell Cycle	pRb	Cyclin D/Cdk4,6; Cyclin E/Cdk2	
Cell Cycle	Ras	EGFR; FGFR2; IL-6; VEGF; IR; HGF	p53; GR
Apoptosis	Bad	p53; TNF-α	Akt; MAPK; NF-κB; p21
Apoptosis	Bax	Bim; p53	Akt; Bcl-2; Sox4
Apoptosis	Bcl-2	Integrin; NF-κB; VEGF	Caspase 3; p53; TGF-β
Apoptosis	Bcl-xL	EGFR; NF-κB; Stat5	Caspase 3
Apoptosis	Bim		Akt; MAPK
Apoptosis	Caspase 3	Bad; Cytochrome C; Fas; TNF-α	NF-κB; XIAP
Apoptosis	Cytochrome C	Bad; Bax; Caspase 3	Bcl-2; Bcl-xL; p21
Apoptosis	Fas	TNF-α	Ras
Apoptosis	XIAP	Akt; Integrin; MAPK; NF-κB; RARs	Caspase 3; Cytochrome C
Differentiation	C/EBPα	C/EBPβ; HNF4α; NOG; Runx1	HIF; IL-1; MAPK; Notch; SHH; Stat5; NR2F2
Differentiation	C/EBPβ	AP2; IL-1; MAPK	Hoxa3; TGF-β
Differentiation	Cdx2	BMP; c-Jun; Gata1/2; HNF1; HNF4α; MAPK; Sox2; Wnt	C/EBPβ; Sox9; Stat3; TGF-β
Differentiation	Foxa1	AP2; Foxa2; RARs	Sox4
Differentiation	Foxa2	C/EBPα; RARs; SHH	Akt; PRDM14
Differentiation	Gata1/2	Cdx2; Notch; Stat3	c-Jun; Hes1; Hoxa10; VEGF; Pu.1
Differentiation	Gata4/6	PKA; Sox7; Wnt	c-Jun; Hey2; PRDM14; Sox2
Differentiation	Hes1	Notch; TGF-β	
Differentiation	Hey2	Notch	
Differentiation	HNF1	HNF4α; Notch	C/EBPα
Differentiation	HNF4α	C/EBPα; Cdx2; E-Cadherin; Gata4/6; HNF1; Stat5; NR2F2	MAPK; NF-κB; p53; PKA; Sox2; Stat3; IR; NR4A
Differentiation	Hoxa10	BMP; Hoxa9; Stat5	Cdx2; IL-1
Differentiation	Hoxa3	c-Jun; RARs; Wnt	Stat3
Differentiation	Hoxa5	Cdx2; Hoxa10; RARs	Runx1
Differentiation	Hoxa9	C/EBPα; Notch; Stat5	NF-κB; TGF-β
Differentiation	PGC-1	C/EBPβ	p53; Akt; c-Jun
Differentiation	PRDM14	Stat3	
Differentiation	Pu.1	C/EBPα; Runx1	Gata1/2; Sox4
Differentiation	Runx1	BMP; Cdx2; c-Jun; Gata1/2; Sox2; Notch	Hoxa3; Stat5
Differentiation	Runx2	BMP; Hoxa10; Runx1	C/EBPβ; FGFR3; Notch; Stat5; NR2F2; TGF-β
Differentiation	Sox2	AP2; FGFR2; Stat3; Wnt	p21; Cdx2
Differentiation	Sox4	Sox7; Stat5; TGF-β; Wnt	C/EBPα
Differentiation	Sox7	BMP; c-Jun; VEGF	PRDM14
Differentiation	Sox9	AP2; BMP; FGFR3; Notch; PKA; SHH; TGF-β; Wnt	c-Jun; IL-1; NF-κB; RARs
NR	PPARγ	C/EBPα; C/EBPβ	RARs; SHH; Stat5; TGF-β; TNF-α; NR2F2
NR	GR	HNF1; TGF-β	NR4A; NR2F2
NR	NR4A	EGFR; VEGF; Runx1	HNF4α; GR
NR	RARs	AP2; PPARγ	MAPK; NR2F2
NR	NR2F2	Gata4/6; HNF4α; MAPK; SHH; Wnt; GR	

Category	Nodes	Activator	Inhibitor
Immune Response	G-CSFR	C/EBPα; NF-κB; Pu.1	GR
Immune Response	IL-1	C/EBPβ; NF-κB; OPN	Akt; C/EBPα
Immune Response	IL-10	Fas; TNF-α	IL-10
Immune Response	IL-3	C/EBPα; Gata1/2; Runx1	
Immune Response	IL-6	C/EBPβ; Hoxa3; NF-κB; NGAL	HNF4α; RARs; Sox2
Immune Response	iκB	NF-κB; TGF-β; NR4A; GR	Akt; EGFR; Fas; TNF-α
Immune Response	NF-κB	C/EBPβ; CCK; IL-1; TNF-α	Cdx2; Foxa1; IL-10; iκB; PPARγ; Sox2; IR
Immune Response	Stat3	CCK; FGFR2; FGFR3; G-CSFR; FGFR4; Gast; IL-6; OPN; VEGF; HGF	Gata1/2; PPARγ; PTEN; RARs
Immune Response	Stat5	FGFR2; IL-3; VEGF; IR; G-CSFR	HNF4α; Runx1; Runx2; TGF-β
Immune Response	TGF-β	Hoxa10; Hoxa3; Hoxa9; NOG; OPN; p53; Sox7; Stat3; TNF-α	Gata4/6; NF-κB; RARs; Sox2; Stat5; NR2F2
Immune Response	TNF-α	IL-1; NF-κB	IL-10
Growth Factors	BMP	EGFR; FGFR2; Gata4/6; Runx1	HNF4α; NOG; TGF-β; NR2F2
Growth Factors	CCK	C/EBPβ; Cdx2; Gast; Gata4/6; IL-1; IR	
Growth Factors	EGFR	Myc; Runx1; Sox4; HIF	PTEN
Growth Factors	FGF2	Hoxa10; HSPG2	
Growth Factors	FGF4/8	SHH	Notch
Growth Factors	FGF7	HSPG2; IL-1	
Growth Factors	FGF9	Sox9	
Growth Factors	FGFR2	FGF2; FGF7; Hoxa10; Runx2	PRDM14
Growth Factors	FGFR3	FGF9	
Growth Factors	FGFR4	FGF4/8; FGF9	
Growth Factors	Gast	Sox2; TGF-β; Wnt	
Growth Factors	HGF	c-Jun; NF-κB; HIF	GR; TGF-β
Growth Factors	IR	C/EBPα; PPARγ; SHH; Wnt	Notch; TNF-α
Growth Factors	VEGF	Gata4/6; HIF; PGC-1; Stat3; TGF-β; NR2F2; NR4A	RARs; Sox9; Notch; Runx1
Stress Response	Akt	CCK; c-Jun; EGFR; FGFR2; Notch; NF-κB; PKA; Ras; RARs; IR; HGF	PTEN
Stress Response	AP2	C/EBPβ; RARs; NR2F2	
Stress Response	c-Jun	Akt; CCK; EGFR; FGFR3; Runx1; TNF-α	C/EBPα; Mkp2; GR
Stress Response	HIF	Akt; Stat3; NR4A	p53
Stress Response	MAPK	FGFR2; FGFR3; Integrin; NF-κB; PPARγ; Ras; NR2F2	PTEN; TGF-β
Stress Response	Mkp2	Hoxa10	
Stress Response	PKA	Akt; EGFR; TGF-β	p53
Stress Response	PTEN	AP2; p53; PPARγ	NF-κB; Stat3
Stress Response	p53	AP2; Myc; PPARγ; PTEN; RARs	Akt; Notch; Sox4
ECM	E-Cadherin	AP2; Cdx2; Foxa1; Foxa2; HNF4α	Akt; TGF-β; EGFR; HIF; SHH; Stat3; TNF-α
ECM	HSPG2	Runx2; TGF-β	
ECM	Integrin	EGFR; MAPK; TNF-α; VEGF	Myc; Ras; SHH
ECM	Notch	NF-κB; Cdx2; FGFR2; HNF1; MAPK; Stat3	BMP; C/EBPα; E-Cadherin; Hoxa5; NR2F2
ECM	NGAL	Runx1; TNF-α	
ECM	NOG	SHH; Sox9; Wnt	FGFR2
ECM	OPN	Runx1; Runx2; TGF-β	PGC-1
ECM	SHH	Foxa1; Sox2; TGF-β	BMP; FGFR2; Foxa2; Hes1
ECM	Wnt	EGFR; FGFR2; FGFR3; Gata4/6; PGC-1; Sox4; TGF-β; IR; HGF	Cdx2; HNF4α; Sox7; Sox9

[1] C. S. Fuchs, E. L. Giovannucci, G. A. Colditz, D. J. Hunter, F. E. Speizer, and W. C. Willett, N Engl J Med **331**, 1669 (1994).

[2] L. E. Johns and R. S. Houlston, Am J Gastroenterol **96**, 2992 (2001).

[3] D. E. Goldgar, D. F. Easton, L. A. Cannon-Albright, and M. H. Skolnick, J Natl Cancer Inst **86**, 1600 (1994).

[4] D. P. Taylor, R. W. Burt, M. S. Williams, P. J. Haug, and L. A. Cannon-Albright, Gastroenterol **138**, 877 (2010).

[5] F. Armelao and G. de Pretis, World J Gastroenterol **20**, 9292 (2014).

[6] K. W. Jasperson, T. M. Tuohy, D. W. Neklason, and R. W. Burt, Gastroenterol **138**, 2044 (2010).

[7] US Cancer Statistics Working Group, "United States cancer statistics: 1999–2010 incidence and mortality web-based report," (2014).

[8] E. R. Fearon, Annu Rev Pathol **6**, 479 (2011).

[9] P. Polakis, Genes Dev **14**, 1837 (2000).

[10] N. A. Theodosiou and C. J. Tabin, Dev Biol **259**, 258 (2003).

[11] V. Korinek, N. Barker, P. Moerer, E. van Donselaar, G. Huls, P. J. Peters, and H. Clevers, Nat Genet **19**, 379 (1998).

[12] R. I. Sherwood, R. Maehr, E. O. Mazzoni, and D. A. Melton, Mech Dev **128**, 387 (2011).

[13] N. Gao, P. White, and K. H. Kaestner, Dev Cell **16**, 588 (2009).

[14] D. J. Roberts, Dev Dyn **219**, 109 (2000).

[15] M. Kedinger, P. Simon-Assmann, F. Bouziges, C. Arnold, E. Alexandra, and K. Haffen, Differentiation **43**, 87 (1990).

[16] Å. Apelqvist, U. Ahlgren, and H. Edlund, Curr Biol **7**, 801 (1997).

[17] M. A. Huber, N. Kraut, and H. Beug, Curr Opin Cell Biol **17**, 548 (2005).

[18] B. Z. Stanger, R. Datar, L. C. Murtaugh, and D. A. Melton, Proc Natl Acad Sci USA **102**, 12443 (2005).

[19] Z. Wang, Y. Li, D. Kong, and F. H. Sarkar, Curr Drug Targets **11**, 745 (2010).

[20] K. C. Schreck, P. Taylor, L. Marchionni, V. Gopalakrishnan, E. E. Bar, N. Gaiano, and C. G. Eberhart, Clin Cancer Res **16**, 6060 (2010).

[21] M. L. Slattery and F. Fitzpatrick, Cancer Prev Res **2**, 922 (2009).

[22] S. H. Itzkowitz and X. Yio, Am J Physiol **287**, G7 (2004).

[23] D. G. Silberg, G. P. Swain, E. R. Suh, and P. G. Traber, Gastroenterol **119**, 961 (2000).

[24] C. Scholl, D. Bansal, K. Döhner, K. Eiwen, B. J. Huntly, B. H. Lee, F. G. Rücker, R. F. Schlenk, L. Bullinger, H. Döhner, *et al.*, J Clin Invest **117**, 1037 (2007).

[25] M. P. Pradhan, N. K. Prasad, and M. J. Palakal, BMC Cancer **12**, 331 (2012).

[26] C. J. F. Scheitz, T. S. Lee, D. J. McDermitt, and T. Tumbar, EMBO J **31**, 4124 (2012).

[27] D. Levanon, O. Brenner, V. Negreanu, D. Bettoun, E. Woolf, R. Eilam, J. Lotem, U. Gat, F. Otto, N. Speck, *et al.*, Mech Dev **109**, 413 (2001).

[28] T. Yamagata, K. Maki, and K. Mitani, Int J Hematol **82**, 1 (2005).

[29] C. Degirolamo, S. Modica, G. Palasciano, and A. Moschetta, Trends Mol Med **17**, 564 (2011).

[30] C. G. Su, X. Wen, S. T. Bailey, W. Jiang, S. M. Rangwala, S. A. Keilbaugh, A. Flanigan, S. Murthy, M. A. Lazar, and G. D. Wu, J Clin Invest **104**, 383 (1999).

[31] L. Dubuquoy, E. Å. Jansson, S. Deeb, S. Rakotobe, M. Karoui, J.-F. Colombel, J. Auwerx, S. Pettersson, and P. Desreumaux, Gastroenterol **124**, 1265 (2003).

[32] A. Quaroni, J. Q. Tian, M. Göke, and D. K. Podolsky, Am J Physiol **277**, G1027 (1999).

[33] M. A. Maxwell and G. E. M. Muscat, Nucl Recept Singal **4**, e002 (2006).

[34] B. You, Y.-Y. Jiang, S. Chen, G. Yan, and J. Sun, Circ Res **104**, 742 (2009).

[35] C. Martens, S. Bilodeau, M. Maira, Y. Gauthier, and J. Drouin, Mol Endocrinol **19**, 885 (2005).

[36] H. Zeng, L. Qin, D. Zhao, X. Tan, E. J. Manseau, M. Van Hoang, D. R. Senger, L. F. Brown, J. A. Nagy, and H. F. Dvorak, J Exp Med **203**, 719 (2006).

[37] P. A. Marshall, Z. Hernandez, I. Kaneko, T. Widener, C. Tabacaru, I. Aguayo, and P. W. Jurutka, J Steroid Biochem Mol Biol **132**, 147 (2012).

F. Network definition and reference for molecular interactions

Table S2. Molecular interactions in the CRC network with references.

	Activated By	Inhibited By	Notes
		**Cell Cycle**	
Cyclin D/Cdk4,6	E2F [1], EGFR [2], Myc [1], Pu.1 [3]	p21 [1, 4], p53 [1]	
Cyclin E/Cdk2	E2F [1], Myc [5]	PTEN [1], p21 [1, 4], p27 [1, 4]	
E2F	E2F [1], Myc [1]	p21 [4]	
Myc	E2F [6], MAPK [6, 7], Notch [8], SHH [9], Wnt [10], pRb [6]	AP2 [11], TGF-β [12, 13], p53 [6]	
p21	AP2 [14], GR [15], Gata1/2 [16], HNF4α [17], Runx1 [18], TGF-β [13], TNF-α [19], p53 [6, 19]	Akt [4], Hes1 [20], Myc [19, 21]	
p27	E-Cadherin [22], Gata1/2 [23], NR4A [24], PTEN [25], TGF-β [26]	Akt [25], Hes1 [27], Myc [6]	
pRb	Cyclin D/Cdk4,6 [1, 28], Cyclin E/Cdk2 [1, 28]		Retinoblastoma protein phosphorylation
Ras	EGFR [29], FGFR2 [30], HGF [31], IL-6 [32, 33], IR [34], VEGF [35, 36]	GR [37], p53 [38]	
		**Apoptosis**	
Bad	TNF-α [39], p53 [40]	Akt [41], MAPK [41], NF-κB [42], p21 [43]	
Bax	Bim [44], p53 [45, 46]	Akt [47], Bcl-2 [48], Sox4 [49]	
Bcl-2	Integrin [50], NF-κB [51], VEGF [52]	Caspase 3 [52], TGF-β [53], p53 [54]	
Bcl-xL	EGFR [55], NF-κB [56], Stat5 [57]	Caspase 3 [58]	
Bim		Akt [41], MAPK [41]	
Caspase 3	Bad [59, 60], Cytochrome C [46, 52, 61], Fas [62], TNF-α [63]	NF-κB [64], XIAP [46, 52, 65, 66]	
Cytochrome C	Bad [52], Bax [52], Caspase 3 [46, 52, 65, 67]	Bcl-2 [68, 69], Bcl-xL [68, 70], p21 [71, 72]	Cytochrome C, Apaf-1, and Caspase 9 complex
Fas	TNF-α [73]	Ras [74]	
XIAP	Akt [66], Integrin [75], MAPK [76], NF-κB [67, 77], RARs [78]	Caspase 3 [46, 52, 54, 65–67], Cytochrome C [46, 52, 67]	X-linked inhibitor of apoptosis
		**Differentiation**	
C/EBPα	C/EBPβ [79], HNF4α [80], NOG [81], Runx1 [82]	HIF [83], IL-1 [84], MAPK [85], NR2F2 [86], Notch [87], SHH [88], Stat5 [89]	
C/EBPβ	AP2 [90], IL-1 [84], MAPK [91]	Hoxa3 [92], TGF-β [93]	

Continued on next page

Table S2. Continued from previous page

	Activated By	Inhibited By	Notes
Cdx2	BMP [94], Gata1/2 [95, 96], HNF1 [97], HNF4α [98], MAPK [99], Sox2 [100], Wnt [101], c-Jun [102]	*C/EBPβ [102, 103], Sox9 [104], Stat3 [105], TGF-β [106]	*C/EBPβ downregulate Cdx2 by c-Fos upregulation
Foxa1	AP2 [107], Foxa2 [108], RARs [109, 110]	Sox4 [111]	
Foxa2	C/EBPα [112], RARs [109], SHH [113]	Akt [114], PRDM14 [115]	
Gata1/2	Cdx2 [116], Notch [117], Stat3 [118]	Hes1 [119], Hoxa10 [120], Pu.1 [121, 122], VEGF [123], c-Jun [124]	
Gata4/6	PKA [125], Sox7 [126], Wnt [127]	Hey2 [128], PRDM14 [115], Sox2 [100, 126, 129], c-Jun [130]	
Hes1	Notch [119], TGF-β [131]		
Hey2	Notch [132]		
HNF1	HNF4α [133], Notch [134]	C/EBPα [135]	
HNF4α	C/EBPα [85], Cdx2 [98], E-Cadherin [136], Gata4/6 [137], HNF1 [133], NR2F2 [138], Stat5 [139]	IR [140], MAPK [141], NF-κB [142], *NR4A [143, 144], PKA [140], Sox2 [145], Stat3 [146], p53 [147]	*indirect through competing available p300
Hoxa10	BMP [148], Hoxa9 [149], *Stat5 [150, 151]	Cdx2 [152], IL-1 [153]	*indirect
Hoxa3	RARs [154, 155], Wnt [156], c-Jun [157]	Stat3 [158]	
Hoxa5	Cdx2 [159], Hoxa10 [120], RARs [155]	Runx1 [160]	
Hoxa9	C/EBPα [161, 162], Notch [163], Stat5 [161]	NF-κB [164], TGF-β [165]	
PGC-1	C/EBPβ [166]	Akt [167], c-Jun [168], p53 [169]	
PRDM14	Stat3 [170]		
Pu.1	C/EBPα [171], Runx1 [172, 173]	Gata1/2 [121, 122], Sox4 [174]	
Runx1	BMP [175], Cdx2 [116], Gata1/2 [176], Notch [177], Sox2 [178], c-Jun [179]	Hoxa3 [92, 180], Stat5 [181, 182]	
Runx2	BMP [13], Hoxa10 [148], *Runx1 [183]	C/EBPβ [184], FGFR3 [185], NR2F2 [186], Notch [187], Stat5 [182], TGF-β [183]	*indirect
Sox2	AP2 [188], FGFR2 [189], Stat3 [100], Wnt [190]	*Cdx2 [191], p21 [192]	*indirect
Sox4	Sox7 [193], Stat5 [194], TGF-β [195, 196], Wnt [193]	C/EBPα [197]	
Sox7	BMP [198], VEGF [199], c-Jun [200]	PRDM14 [201]	
Sox9	AP2 [202], BMP [203], FGFR3 [204], Notch [134, 205], PKA [206], SHH [207], TGF-β [208], Wnt [104, 209]	IL-1 [210], NF-κB [210, 211], RARs [211, 212], c-Jun [213]	
Nuclear Receptor (NR)			
PPARγ	C/EBPα [214], C/EBPβ [215]	NR2F2 [86], RARs [216], SHH [88], Stat5 [217], TGF-β [216], TNF-α [218]	
GR	HNF1 [219], TGF-β [220]	NR2F2 [221], NR4A [222]	

Continued on next page

Table S2. Continued from previous page

	Activated By	Inhibited By	Notes
NR4A	EGFR [24], Runx1 [223], VEGF [224]	GR [225], *HNF4α [143, 144]	*indirect through competing available p300
RARs	AP2 [226], PPARγ [227]	MAPK [228], NR2F2 [229]	Retinoic acid receptor signaling
NR2F2	GR [221], Gata4/6 [230, 231], HNF4α [138], MAPK [232], SHH [233], Wnt [86]		
		Immune Response	
G-CSR	C/EBPα [234], NF-κB [235], Pu.1 [236]	GR [237]	
IL-1	C/EBPβ [238], NF-κB [239], OPN [240]	Akt [241], C/EBPα [242]	Interleukin 1
IL-10	Fas [239], TNF-α [239]	IL-10 [239]	Interleukin 10
IL-3	C/EBPα [243], Gata1/2 [244], Runx1 [245–247]		Interleukin 3
IL-6	C/EBPβ [248], Hoxa3 [249], NF-κB [250], NGAL [251]	HNF4α [146], RARs [252], Sox2 [253]	Interleukin 6
iκB	GR [237], NF-κB [77, 254, 255], NR4A [256], TGF-β [257]	Akt [258], EGFR [259], Fas [260], TNF-α [77, 254, 255]	
NF-κB	C/EBPβ [261], CCK [262, 263], IL-1 [77, 254, 255, 264], TNF-α [77, 254, 255]	Cdx2 [265], Foxa1 [107], IL-10 [266], IR [267], PPARγ [268], Sox2 [269], iκB [77, 254, 255]	
Stat3	CCK [270], FGFR2 [271], FGFR3 [272], FGFR4 [273], G-CSR [274], Gast [275], HGF [31], IL-6 [276], OPN [277], VEGF [278]	Gata1/2 [279], PPARγ [280], PTEN [281, 282], RARs [283]	
Stat5	FGFR2 [284], G-CSR [274], IL-3 [57], IR [285], VEGF [286]	HNF4α [139], Runx1 [181], Runx2 [181], TGF-β [287]	
TGF-β	Hoxa10 [288], Hoxa3 [92], Hoxa9 [289], NOG [290], OPN [290], Sox7 [291], Stat3 [292], TNF-α [13, 293], p53 [294, 295]	Gata4/6 [296], NF-κB [13], NR2F2 [297], RARs [298], Sox2 [299], Stat5 [300]	Transforming growth factor beta
TNF-α	IL-1 [77, 254, 255], NF-κB [77, 254, 255]	IL-10 [77, 254, 255]	
		Growth Factors	
BMP	EGFR [301], FGFR2 [302], Gata4/6 [303], Runx1 [304]	HNF4α [305], NOG [306], NR2F2 [307], TGF-β [308]	
CCK	C/EBPβ [309], Cdx2 [310], Gast [309, 311], Gata4/6 [312], IL-1 [313–315], IR [316]		
EGFR	HIF [317], Myc [318], Runx1 [319], Sox4 [196]	PTEN [320]	
FGF2	HSPG2 [321], Hoxa10 [288]		Fibroblast growth factor 2

Continued on next page

Table S2. Continued from previous page

	Activated By	Inhibited By	Notes
FGF4/8	SHH [322]	Notch [323]	
FGF7	HSPG2 [321], IL-1 [324]		Fibroblast growth factor 7
FGF9	Sox9 [325]		
FGFR2	FGF2 [271], FGF7 [271], Hoxa10 [288], Runx2 [326]	PRDM14 [327]	Fibroblast growth factor receptor 2
FGFR3	FGF9 [328]		
FGFR4	FGF4/8 [329], FGF9 [329]		
Gast	Sox2 [145], TGF-β [330, 331], Wnt [330, 331]		
HGF	HIF [31], NF-κB [332], c-Jun [332]	GR [333], TGF-β [333]	
IR	C/EBPα [214], PPARγ [334], SHH [335], Wnt [336]	Notch [337], TNF-α [338]	
VEGF	Gata4/6 [339], HIF [340], NR2F2 [341], NR4A [342], PGC-1 [343], Stat3 [344–346], TGF-β [347, 348]	Notch [349], RARs [350], Runx1 [351], Sox9 [352]	Vascular endothelial growth factor
	Stress Response		
Akt	CCK [311, 353], EGFR [354], FGFR2 [271], HGF [31], IR [355], NF-κB [41, 255], Notch [356], PKA [357], RARs [358], Ras [359], c-Jun [360]	PTEN [361]	
AP2	C/EBPβ [362], NR2F2 [363], RARs [364]		Activating Protein 2 (AP-2)
c-Jun	Akt [365], CCK [366], EGFR [367], FGFR3 [185], Runx1 [179], TNF-α [368]	C/EBPα [369], GR [370, 371], Mkp2 [372]	
HIF	Akt [41, 373], NR4A [374], Stat3 [346]	p53 [373]	Hypoxia-inducible factors
MAPK	FGFR2 [375], FGFR3 [376], Integrin [377, 378], NF-κB [7], NR2F2 [232], PPARγ [379], Ras [35]	PTEN [361, 378], TGF-β [380]	Mitogen-activated protein kinase signaling
Mkp2	Hoxa10 [381]		
PKA	Akt [382], EGFR [383], TGF-β [384, 385]	p53 [386]	
PTEN	AP2 [387], PPARγ [388], p53 [389]	NF-κB [390], Stat3 [281]	
p53	AP2 [391], Myc [35], PPARγ [392], PTEN [393], RARs [392, 394]	Akt [35], Notch [395], Sox4 [396]	
	ECM (Extracellular Matrix)		
E-Cadherin	AP2 [397], Cdx2 [398], Foxa1 [109, 399], Foxa2 [399], HNF4α [400]	Akt [401, 402], EGFR [403], HIF [404, 405], SHH [406], Stat3 [275, 407], TGF-β [408], TNF-α [409]	

Continued on next page

Table S2. Continued from previous page

	Activated By	Inhibited By	Notes
HSPG2	Runx2 [410], TGF-β [411]		
Integrin	EGFR [50, 377], MAPK [412], TNF-α [377, 413], VEGF [50, 377]	Myc [414], Ras [415], SHH [416]	
Notch	Cdx2 [417, 418], FGFR2 [419], HNF1 [97], MAPK [420], NF-κB [421], Stat3 [422]	BMP [423], C/EBPα [424], E-Cadherin [425], Hoxa5 [426], NR2F2 [427]	
NGAL	Runx1 [428], TNF-α [429]		
NOG	SHH [430], Sox9 [431], Wnt [430]	FGFR2 [432]	Noggin
OPN	Runx1 [428], Runx2 [428], TGF-β [433]	*PGC-1 [434, 435]	Osteopontin, *indirect
SHH	Foxa1 [108], Sox2 [178], TGF-β [436]	BMP [437, 438], FGFR2 [439], Foxa2 [108], Hes1 [440]	Sonic hedgehog pathway
Wnt	EGFR [441], FGFR2 [441], FGFR3 [441], Gata4/6 [442], HGF [443, 444], IR [445], PGC-1 [446], Sox4 [196], TGF-β [447]	Cdx2 [448, 449], HNF4α [450, 451], Sox7 [452, 453], Sox9 [454]	

[1] A. W. Murray and T. Hunt, *The Cell Cycle: An Introduction* (Oxford University Press, Oxford, 1993).

[2] Y. Tao, X. Song, X. Deng, D. Xie, L. M. Lee, Y. Liu, W. Li, L. Li, L. Deng, Q. Wu, *et al.*, Exp Cell Res **303**, 240 (2005).

[3] K. S. Choe, O. Ujhelly, S. N. Wontakal, and A. I. Skoultchi, J Biol Chem **285**, 3044 (2010).

[4] C. J. Sherr and J. M. Roberts, Genes Dev **13**, 1501 (1999).

[5] A. J. Obaya, M. K. Mateyak, and J. M. Sedivy, Oncogene **18**, 2934 (1999).

[6] C. V. Dang, Mol Cell Biol **19**, 1 (1999).

[7] G. Pearson, F. Robinson, T. B. Gibson, B.-e. Xu, M. Karandikar, K. Berman, and M. H. Cobb, Endocr Rev **22**, 153 (2001).

[8] T. Palomero, W. K. Lim, D. T. Odom, M. L. Sulis, P. J. Real, A. Margolin, K. C. Barnes, J. O'Neil, D. Neuberg, A. P. Weng, *et al.*, Proc Natl Acad Sci USA **103**, 18261 (2006).

[9] B. A. Hatton, P. S. Knoepfler, A. M. Kenney, D. H. Rowitch, I. M. de Alborán, J. M. Olson, and R. N. Eisenman, Cancer Res **66**, 8655 (2006).

[10] S. Zhang, Y. Li, Y. Wu, K. Shi, L. Bing, and J. Hao, Anat Rec **295**, 2104 (2012).

[11] L. Yu, M. J. Hitchler, W. Sun, E. H. Sarsour, P. C. Goswami, A. J. Klingelhutz, and F. E. Domann, J Oncol **2009** (2009).

[12] J. Romero-Gallo, E. G. Sozmen, A. Chytil, W. E. Russell, R. Whitehead, W. T. Parks, M. S. Holdren, M. F. Her, S. Gautam, M. Magnuson, *et al.*, Oncogene **24**, 3028 (2005).

[13] J. Massagué, J. Seoane, and D. Wotton, Genes Dev **19**, 2783 (2005).

[14] Y.-X. Zeng, K. Somasundaram, and W. S. El-Deiry, Nat Genet **15**, 78 (1997).

[15] C. Harms, K. Albrecht, U. Harms, K. Seidel, L. Hauck, T. Baldinger, D. Hübner, G. Kronenberg, J. An, K. Ruscher, *et al.*, J Neurosci **27**, 4562 (2007).

[16] M. Papetti, S. N. Wontakal, T. Stopka, and A. I. Skoultchi, Cell Cycle **9**, 1972 (2010).

[17] U. Apte, A. Bell, C. Walesky, G. Edwards, and A. Wolfe, FASEB J (2010).

[18] C. S. Hoi, S. E. Lee, S.-Y. Lu, D. J. McDermitt, K. M. Osorio, C. M. Piskun, R. M. Peters, R. Paus, and T. Tumbar, Mol Cell Biol **30**, 2518 (2010).

[19] A. L. Gartel and S. K. Radhakrishnan, Cancer Res **65**, 3980 (2005).

[20] P. Monahan, S. Rybak, and L. T. Raetzman, Endocrinology **150**, 4386 (2009).

[21] J. Seoane, H.-V. Le, and J. Massagué, Nature **419**, 729 (2002).

[22] B. S. Croix, C. Sheehan, J. W. Rak, V. A. Flørenes, J. M. Slingerland, and R. S. Kerbel, J Cell Biol **142**, 557 (1998).

[23] S. Ezoe, I. Mtsumura, M. Mizuki, A. Kawasaki, T. Machii, M. Yamamoto, T. Enver, and Y. Kanakura, Blood **98**, 759A (2001).

[24] J. Martínez-González and L. Badimon, Cardiovasc Res **65**, 609 (2005).

[25] A. R. Gottschalk, D. Basila, M. Wong, N. M. Dean, C. H. Brandts, D. Stokoe, and D. A. Haas-Kogan, Cancer Res **61**, 2105 (2001).

[26] J. Lecanda, V. Ganapathy, C. D'Aquino-Ardalan, B. Evans, C. Cadacio, A. Ayala, and L. I. Gold, Cell Cycle **8**, 742 (2009).

[27] K. Murata, M. Hattori, N. Hirai, Y. Shinozuka, H. Hirata, R. Kageyama, T. Sakai, and N. Minato, Mol Cell Biol **25**, 4262 (2005).

[28] R. A. Weinberg, Cell **81**, 323 (1995).

[29] J. Mendelsohn and J. Baselga, Oncogene **19**, 6550 (2000).

[30] M. R. Ramsey, C. Wilson, B. Ory, S. M. Rothenberg, W. Faquin, A. A. Mills, L. W. Ellisen, *et al.*, J Clin Invest **123**, 3525 (2013).

[31] S. L. Organ and M.-S. Tsao, Ther Adv Med Oncol **3**, S7 (2011).

[32] J. R. Jackson, M. Seed, C. Kircher, D. Willoughby, and J. Winkler, FASEB J **11**, 457 (1997).

[33] T. Cohen, D. Nahari, L. W. Cerem, G. Neufeld, and B.-Z. Levi, J Biol Chem **271**, 736 (1996).

[34] K. Yonezawa, A. Ando, Y. Kaburagi, R. Yamamoto-Honda, T. Kitamura, K. Hara, M. Nakafuku, Y. Okabayashi, T. Kadowaki, and Y. Kaziro, J Biol Chem **269**, 4634 (1994).

[35] A. B. Vojtek and C. J. Der, J Biol Chem **273**, 19925 (1998).

[36] A. J. Dannenberg, S. M. Lippman, J. R. Mann, K. Subbaramaiah, and R. N. DuBois, J Clin Oncol **23**, 254 (2005).

[37] S. B. Eastman-Reks and W. V. Vedeckis, Cancer Res **46**, 2457 (1986).

[38] P. S. Nelson, N. Clegg, H. Arnold, C. Ferguson, M. Bonham, J. White, L. Hood, and B. Lin, Proc Natl Acad Sci USA **99**, 11890 (2002).

[39] S. Álvarez, A. Blanco, M. Fresno, and M. Á. Muñoz-Fernández, PLoS One **6**, e16100 (2011).

[40] L.-H. Chen, C.-Y. Hsu, C.-F. Weng, *et al.*, World J Gastroenterol **12**, 5175 (2006).

[41] J. E. Thompson and C. B. Thompson, J Clin Oncol **22**, 4217 (2004).

[42] R. Cianfrocca, M. Muscolini, V. Marzano, A. Annibaldi, B. Marinari, M. Levrero, A. Costanzo, and L. Tuosto, Cell Death Differ **15**, 354 (2008).

[43] M. Castedo, J. Perfettini, T. Roumier, and G. Kroemer, Cell Death Differ **9**, 1287 (2002).

[44] T. Kuwana, M. R. Mackey, G. Perkins, M. H. Ellisman, M. Latterich, R. Schneiter, D. R. Green, and D. D. Newmeyer, Cell **111**, 331 (2002).

[45] E. L. Soucie, M. G. Annis, J. Sedivy, J. Filmus, B. Leber, D. W. Andrews, and L. Z. Penn, Mol Cell Biol **21**, 4725 (2001).

[46] H. Y. Chang and X. Yang, Microbiol Mol Biol Rev **64**, 821 (2000).

[47] S. J. Gardai, D. A. Hildeman, S. K. Frankel, B. B. Whitlock, S. C. Frasch, N. Borregaard, P. Marrack, D. L. Bratton, and P. M. Henson, J Biol Chem **279**, 21085 (2004).

[48] A. Basu and S. Haldar, Mol Hum Reprod **4**, 1099 (1998).

[49] W. Hur, H. Rhim, C. K. Jung, J. D. Kim, S. H. Bae, J. W. Jang, J. M. Yang, S.-T. Oh, D. G. Kim, H. J. Wang, *et al.*, Carcinogenesis **31**, 1298 (2010).

[50] D. G. Stupack and D. A. Cheresh, J Cell Sci **115**, 3729 (2002).

[51] C.-Y. Wang, D. C. Guttridge, M. W. Mayo, and A. S. Baldwin, Mol Cell Biol **19**, 5923 (1999).

[52] I. Budihardjo, H. Oliver, M. Lutter, X. Luo, and X. Wang, Annu Rev Cell Dev Biol **15**, 269 (1999).

[53] M. M. Ahmed, R. A. Alcock, D. Chendil, S. Dey, A. Das, K. Venkatasubbarao, M. Mohiuddin, L. Sun, W. E. Strodel, and J. W. Freeman, J Biol Chem **277**, 2234 (2002).

[54] S. B. Bratton, G. Walker, S. M. Srinivasula, X.-M. Sun, M. Butterworth, E. S. Alnemri, and

G. M. Cohen, EMBO J **20**, 998 (2001).

[55] J. Rueddel, V. E. Wennekes, W. Meissner, J. A. Werner, and R. Mandic, Anticancer Res **30**, 4579 (2010).

[56] C. Chen, L. C. Edelstein, and C. Gélinas, Mol Cell Biol **20**, 2687 (2000).

[57] S. Dumon, S. C. R. Santos, F. Debierre-Grockiego, V. Gouilleux-Gruart, L. Cocault, C. Boucheron, P. Mollat, S. Gisselbrecht, and F. Gouilleux, Oncogene **18**, 4191 (1999).

[58] J. M. Adams and S. Cory, Science **281**, 1322 (1998).

[59] J. Won, D. Y. Kim, M. La, D. Kim, G. G. Meadows, and C. O. Joe, J Biol Chem **278**, 19347 (2003).

[60] J. E. Springer, R. D. Azbill, S. A. Nottingham, and S. E. Kennedy, J Neurosci **20**, 7246 (2000).

[61] C. Adrain and S. J. Martin, Trends Biochem Sci **26**, 390 (2001).

[62] T. S. Zheng, S. F. Schlosser, T. Dao, R. Hingorani, I. N. Crispe, J. L. Boyer, and R. A. Flavell, Proc Natl Acad Sci USA **95**, 13618 (1998).

[63] X. Zhao, B. Bausano, B. R. Pike, J. K. Newcomb-Fernandez, K. K. Wang, E. Shohami, N. Ringger, S. DeFord, D. K. Anderson, and R. L. Hayes, J Neurosci Res **64**, 121 (2001).

[64] L. He, B. Y. Kim, K. A. Kim, O. Kwon, S. O. Kim, E. Y. Bae, M. S. Lee, M. S. Kim, M. Jung, A. Moon, *et al.*, Cell Signal **19**, 1713 (2007).

[65] H. Zou, R. Yang, J. Hao, J. Wang, C. Sun, S. W. Fesik, J. C. Wu, K. J. Tomaselli, and R. C. Armstrong, J Biol Chem **278**, 8091 (2003).

[66] Z. Liu, H. Li, M. Derouet, J. Filmus, E. C. LaCasse, R. G. Korneluk, R. S. Kerbel, and K. V. Rosen, J Biol Chem **280**, 37383 (2005).

[67] Q. L. Deveraux and J. C. Reed, Genes Dev **13**, 239 (1999).

[68] D. T. Chao and S. J. Korsmeyer, Annu Rev Immunol **16**, 395 (1998).

[69] Z. Zhang, S. M. Lapolla, M. G. Annis, M. Truscott, G. J. Roberts, Y. Miao, Y. Shao, C. Tan, J. Peng, A. E. Johnson, *et al.*, J Biol Chem **279**, 43920 (2004).

[70] J. E. Chipuk, M. Bhat, A. Y. Hsing, J. Ma, and D. Danielpour, J Biol Chem **276**, 26614 (2001).

[71] T. Glaser, B. Wagenknecht, and M. Weller, Oncogene **20**, 4757 (2001).

[72] O. Coqueret, Trends Cell Biol **13**, 65 (2003).

[73] K. Kimura and E. P. Gelmann, J Biol Chem **275**, 8610 (2000).

[74] J. Peli, M. Schröter, C. Rudaz, M. Hahne, C. Meyer, E. Reichmann, and J. Tschopp, EMBO J **18**, 1824 (1999).

[75] S. Dong and D. J. Tweardy, Blood **99**, 2637 (2002).

[76] M. Abkhezr, A. R. Keramati, S. N. Ostad, J. Davoodi, and M. H. Ghahremani, Mol Biol Rep **37**, 2037 (2010).

[77] H. L. Pahl, Oncogene **18**, 6853 (1999).

[78] J. Wang, Y. Peng, Y. W. Sun, H. He, S. Zhu, X. An, M. Li, M. Lin, B. Zou, H. H.-X. Xia, *et al.*, Gastroenterol **130**, 747 (2006).

[79] Z. Wu, Y. Xie, N. Bucher, and S. R. Farmer, Genes Dev **9**, 2350 (1995).

[80] A. Bailly, G. Spath, V. Bender, and M. C. Weiss, J Cell Sci **111**, 2411 (1998).

[81] A. Sawant, D. Chanda, T. Isayeva, G. Tsuladze, W. T. Garvey, and S. Ponnazhagan, J Biol Chem **287**, 12241 (2012).

[82] H. Guo, O. Ma, N. A. Speck, and A. D. Friedman, Blood **119**, 4408 (2012).

[83] E. R. Anderson, M. Taylor, X. Xue, A. Martin, D. S. Moons, M. B. Omary, and Y. M. Shah, Mol Cell Biol **32**, 4068 (2012).

[84] M. R. An, C. C. Hsieh, P. D. Reisner, J. P. Rabek, S. G. Scott, D. T. Kuninger, and J. Papaconstantinou, Mol Cell Biol **16**, 2295 (1996).

[85] P. Hatzis, I. Kyrmizi, and I. Talianidis, Mol Cell Biol **26**, 7017 (2006).

[86] M. Okamura, H. Kudo, K.-i. Wakabayashi, T. Tanaka, A. Nonaka, A. Uchida, S. Tsutsumi, I. Sakakibara, M. Naito, T. F. Osborne, *et al.*, Proc Natl Acad Sci USA **106**, 5819 (2009).

[87] H. Yamasaki, A. Sada, T. Iwata, T. Niwa, M. Tomizawa, K. G. Xanthopoulos, T. Koike, and N. Shiojiri, Development **133**, 4233 (2006).

[88] S. Spinella-Jaegle, G. Rawadi, S. Kawai, S. Gallea, C. Faucheu, P. Mollat, B. Courtois, B. Bergaud, V. Ramez, A. M. Blanchet, *et al.*, J Cell Sci **114**, 2085 (2001).

[89] A. T. Wierenga, H. Schepers, M. A. Moore, E. Vellenga, and J. J. Schuringa, Blood **107**, 4326 (2006).

[90] X. Wang, H. A. Pasolli, T. Williams, and E. Fuchs, J Cell Biol **183**, 37 (2008).

[91] M. N. Bradley, L. Zhou, and S. T. Smale, Mol Cell Biol **23**, 4841 (2003).

[92] M. Iacovino, D. Chong, I. Szatmari, L. Hartweck, D. Rux, A. Caprioli, O. Cleaver, and M. Kyba, Nat Cell Biol **13**, 72 (2010).

[93] L. Choy and R. Derynck, J Biol Chem **278**, 9609 (2003).

[94] R. Barros, B. Pereira, I. Duluc, M. Azevedo, N. Mendes, V. Camilo, R. Jacobs, P. Paulo, F. Santos-Silva, I. van Seuningen, *et al.*, J Pathol **215**, 411 (2008).

[95] P. Home, S. Ray, D. Dutta, I. Bronshteyn, M. Larson, and S. Paul, J Biol Chem **284**, 28729 (2009).

[96] H. Bai, T. Sakurai, Y. Someya, T. Konno, A. Ideta, Y. Aoyagi, and K. Imakawa, J Reprod Dev **57**, 518 (2011).

[97] A. D'Angelo, O. Bluteau, M. A. Garcia-Gonzalez, L. Gresh, A. Doyen, S. Garbay, S. Robine, and M. Pontoglio, Development **137**, 1573 (2010).

[98] T. Saandi, F. Baraille, L. Derbal-Wolfrom, A. Cattin, F. Benahmed, E. Martin, P. Cardot, B. Duclos, A. Ribeiro, J. Freund, *et al.*, Oncogene **32**, 3782 (2012).

[99] M. Murohashi, T. Nakamura, S. Tanaka, T. Ichise, N. Yoshida, T. Yamamoto, M. Shibuya, J. Schlessinger, and N. Gotoh, Stem Cells **28**, 113 (2010).

[100] K. Adachi, H. Suemori, S.-y. Yasuda, N. Nakatsuji, and E. Kawase, Genes Cells **15**, 455 (2010).

[101] T. Zhao, Q. Gan, A. Stokes, R. N. Lassiter, Y. Wang, J. Chan, J. X. Han, D. E. Pleasure, J. A. Epstein, and C. J. Zhou, Development **141**, 148 (2014).

[102] O. Lorentz, A. Cadoret, I. Duluc, J. Capeau, C. Gespach, G. Cherqui, and J.-N. Freund, Oncogene **18**, 87 (1999).

[103] J. Liao, G. Piwien-Pilipuk, S. E. Ross, C. L. Hodge, L. Sealy, O. A. MacDougald, and J. Schwartz, J Biol Chem **274**, 31597 (1999).

[104] P. Blache, M. Van De Wetering, I. Duluc, C. Domon, P. Berta, J.-N. Freund, H. Clevers, and P. Jay, J Cell Biol **166**, 37 (2004).

[105] H. Ura, M. Usuda, K. Kinoshita, C. Sun, K. Mori, T. Akagi, T. Matsuda, H. Koide, and T. Yokota, J Biol Chem **283**, 9713 (2008).

[106] K. Selesniemi, M. Reedy, A. Gultice, L. J. Guilbert, and T. L. Brown, Stem Cells Dev **14**, 697 (2005).

[107] A. Naderi, M. Meyer, and D. H. Dowhan, Neoplasia **14**, 283 (2012).

[108] Y. E. Mavromatakis, W. Lin, E. Metzakopian, A. L. Ferri, C. H. Yan, H. Sasaki, J. Whisett, and S.-L. Ang, Mech Dev **128**, 90 (2011).

[109] D. J. DeGraff, P. E. Clark, J. M. Cates, H. Yamashita, V. L. Robinson, X. Yu, M. E. Smolkin, S. S. Chang, M. S. Cookson, M. K. Herrick, *et al.*, PLoS One **7**, e36669 (2012).

[110] J. H. Taube, K. Allton, S. A. Duncan, L. Shen, and M. C. Barton, J Biol Chem **285**, 16135 (2010).

[111] C. D. Scharer, C. D. McCabe, M. Ali-Seyed, M. F. Berger, M. L. Bulyk, and C. S. Moreno, Cancer Res **69**, 709 (2009).

[112] P. C. Martis, J. A. Whitsett, Y. Xu, A.-K. T. Perl, H. Wan, and M. Ikegami, Development **133**, 1155 (2006).

[113] R. D. Bayly, C. Y. Brown, and S. Agarwala, Dev Biol **369**, 32 (2012).

[114] J. J. Howell and M. Stoffel, J Biol Chem **284**, 24816 (2009).

[115] Z. Ma, T. Swigut, A. Valouev, A. Rada-Iglesias, and J. Wysocka, Nat Struct Mol Biol **18**, 120 (2011).

[116] V. P. Rawat, R. K. Humphries, and C. Buske, Blood **120**, 519 (2012).

[117] T. Borggrefe and F. Oswald, Cell Mol Life Sci **66**, 1631 (2009).

[118] K. Kang, H. Jung, S. Nam, and J.-S. Lim, Immune Netw **11**, 348 (2011).

[119] E. Ishiko, I. Matsumura, S. Ezoe, K. Gale, J. Ishiko, Y. Satoh, H. Tanaka, H. Shibayama, M. Mizuki, T. Era, *et al.*, J Biol Chem **280**, 4929 (2005).

[120] M. Magnusson, A. C. Brun, N. Miyake, J. Larsson, M. Ehinger, J. M. Bjornsson, A. Wutz, M. Sigvardsson, and S. Karlsson, Blood **109**, 3687 (2007).

[121] S. N. Wontakal, X. Guo, C. Smith, T. MacCarthy, E. H. Bresnick, A. Bergman, M. P. Snyder, S. M. Weissman, D. Zheng, and A. I. Skoultchi, Proc Natl Acad Sci USA **109**, 3832 (2012).

[122] N. Rekhtman, F. Radparvar, T. Evans, and A. I. Skoultchi, Genes Dev **13**, 1398 (1999).

[123] B. Drogat, J. Kalucka, L. Gutiérrez, H. Hammad, S. Goossens, M. F. Ghahremani, S. Bartunkova, K. Haigh, K. Deswarte, O. Nyabi, *et al.*, Blood **116**, 2141 (2010).

[124] K. E. Elagib, M. Xiao, I. M. Hussaini, L. L. Delehanty, L. A. Palmer, F. K. Racke, M. J. Birrer, G. Shanmugasundaram, M. A. McDevitt, and A. N. Goldfarb, Mol Cell Biol **24**, 7779 (2004).

[125] J. J. Tremblay, F. Hamel, and R. S. Viger, Endocrinology **143**, 3935 (2002).

[126] A. Murakami, H. Shen, S. Ishida, and C. Dickson, J Biol Chem **279**, 28564 (2004).

[127] Y. Tian, L. Yuan, A. M. Goss, T. Wang, J. Yang, J. J. Lepore, D. Zhou, R. J. Schwartz, V. Patel, E. D. Cohen, *et al.*, Dev Cell **18**, 275 (2010).

[128] A. Fischer, J. Klattig, B. Kneitz, H. Diez, M. Maier, B. Holtmann, C. Englert, and M. Gessler, Mol Cell Biol **25**, 8960 (2005).

[129] T. Shirai, S. Miyagi, D. Horiuchi, T. Okuda-Katayanagi, M. Nishimoto, M. Muramatsu, Y. Sakamoto, M. Nagata, K. Hagiwara, and A. Okuda, J Biol Chem **280**, 7244 (2005).

[130] H. Ushijima and M. Maeda, Biochem Biophys Res Commun **423**, 679 (2012).

[131] X. Guo and X.-F. Wang, Cell Res **19**, 71 (2008).

[132] M. Katoh and M. Katoh, Int J Oncol **31**, 461 (2007).

[133] D. T. Odom, N. Zizlsperger, D. B. Gordon, G. W. Bell, N. J. Rinaldi, H. L. Murray, T. L. Volkert, J. Schreiber, P. A. Rolfe, D. K. Gifford, *et al.*, Science **303**, 1378 (2004).

[134] S. Afelik, X. Qu, E. Hasrouni, M. A. Bukys, T. Deering, S. Nieuwoudt, W. Rogers, R. J. MacDonald, and J. Jensen, Development **139**, 1744 (2012).

[135] H. Yamasaki, A. Sada, T. Iwata, T. Niwa, M. Tomizawa, K. G. Xanthopoulos, T. Koike, and N. Shiojiri, Development **133**, 4233 (2006).

[136] G. Peignon, S. Thenet, C. Schreider, S. Fouquet, A. Ribeiro, E. Dussaulx, J. Chambaz, P. Cardot, M. Pinçon-Raymond, and J. Le Beyec, J Biol Chem **281**, 3560 (2006).

[137] E. E. Morrisey, Z. Tang, K. Sigrist, M. M. Lu, F. Jiang, H. S. Ip, and M. S. Parmacek, Genes Dev **12**, 3579 (1998).

[138] M. Boutant, O. H. P. Ramos, C. Lecoeur, E. Vaillant, J. Philippe, P. Zhang, A. Perilhou, B. Valcarcel, S. Sebert, M.-R. Jarvelin, *et al.*, PLoS One **7**, e35810 (2012).

[139] S. Park, C. Wiwi, and D. Waxman, Biochem J **397**, 159 (2006).

[140] K. Hirota, H. Daitoku, H. Matsuzaki, N. Araya, K. Yamagata, S. Asada, T. Sugaya, and A. Fukamizu, J Biol Chem **278**, 13056 (2003).

[141] S. Reddy, W. Yang, D. G. Taylor, X.-q. Shen, D. Oxender, G. Kust, and T. Leff, J Biol Chem **274**, 33050 (1999).

[142] B.-F. Ning, J. Ding, J. Liu, C. Yin, W.-P. Xu, W.-M. Cong, Q. Zhang, F. Chen, T. Han, X. Deng, *et al.*, Hepatology (2014).

[143] J.-C. Wang, J. M. Stafford, and D. K. Granner, J Biol Chem **273**, 30847 (1998).

[144] H. Maruoka, H. Sasaya, K. Sugihara, K. Shimoke, and T. Ikeuchi, J Biochem (Tokyo) **150**, 473 (2011).

[145] L. Raghoebir, E. R. Bakker, J. C. Mills, S. Swagemakers, M. Buscop-van Kempen, A. Boerema-de Munck, S. Driegen, D. Meijer, F. Grosveld, D. Tibboel, *et al.*, J Mol Cell Biol , mjs030 (2012).

[146] M. Hatziapostolou, C. Polytarchou, E. Aggelidou, A. Drakaki, G. A. Poultsides, S. A. Jaeger,

H. Ogata, M. Karin, K. Struhl, M. Hadzopoulou-Cladaras, *et al.*, Cell **147**, 1233 (2011).

[147] Y. Maeda, W. Hwang-Verslues, G. Wei, T. Fukazawa, M. Durbin, L. Owen, X. Liu, and F. Sladek, Biochem J **400**, 303 (2006).

[148] M. Q. Hassan, R. Tare, S. H. Lee, M. Mandeville, B. Weiner, M. Montecino, A. J. Van Wijnen, J. L. Stein, G. S. Stein, and J. B. Lian, Mol Cell Biol **27**, 3337 (2007).

[149] B. Xu, D. Geerts, K. Qian, H. Zhang, and G. Zhu, Hum Reprod **23**, 1394 (2008).

[150] X. Liu and C.-K. Qu, J Signal Transduct **2011** (2011).

[151] N. Chughtai, S. Schimchowitsch, J.-J. Lebrun, and S. Ali, J Biol Chem **277**, 31107 (2002).

[152] C. Scholl, D. Bansal, K. Döhner, K. Eiwen, B. J. Huntly, B. H. Lee, F. G. Rücker, R. F. Schlenk, L. Bullinger, H. Döhner, *et al.*, J Clin Invest **117**, 1037 (2007).

[153] G. Weiss, L. T. Goldsmith, R. N. Taylor, D. Bellet, and H. S. Taylor, Reprod Sci **16**, 216 (2009).

[154] N. Y.-G. Diman, S. Remacle, N. Bertrand, J. J. Picard, S. Zaffran, and R. Rezsohazy, PLoS One **6**, e27624 (2011).

[155] D. Su and L. J. Gudas, Biochem Pharmacol **75**, 1129 (2008).

[156] P. M. In der Rieden, F. L. Vilaspasa, and A. J. Durston, Dev Dyn **239**, 126 (2010).

[157] F. Mechta-Grigoriou, F. Giudicelli, C. Pujades, P. Charnay, and M. Yaniv, Dev Biol **258**, 419 (2003).

[158] B. L. Kidder, J. Yang, and S. Palmer, PLoS One **3**, e3932 (2008).

[159] V. P. Rawat, S. Thoene, V. M. Naidu, N. Arseni, B. Heilmeier, K. Metzeler, K. Petropoulos, A. Deshpande, L. Quintanilla-Martinez, S. K. Bohlander, *et al.*, Blood **111**, 309 (2008).

[160] K. Ross, A. K. Sedello, G. P. Todd, M. Paszkowski-Rogacz, A. W. Bird, L. Ding, T. Grinenko, K. Behrens, N. Hubner, M. Mann, *et al.*, Blood **119**, 4152 (2012).

[161] Y. Huang, K. Sitwala, J. Bronstein, D. Sanders, M. Dandekar, C. Collins, G. Robertson, J. MacDonald, T. Cezard, M. Bilenky, *et al.*, Blood **119**, 388 (2012).

[162] C. Collins, J. Wang, H. Miao, J. Bronstein, H. Nawer, T. Xu, M. Figueroa, A. G. Muntean, and J. L. Hess, Proc Natl Acad Sci USA **111**, 9899 (2014).

[163] F. Weerkamp, T. Luis, B. Naber, E. Koster, L. Jeannotte, J. van Dongen, and F. Staal, Leukemia **20**, 1967 (2006).

[164] C. M. Trivedi, R. C. Patel, and C. V. Patel, Gene **408**, 187 (2008).

[165] N. Wang, H.-G. Kim, C. V. Cotta, M. Wan, Y. Tang, C. A. Klug, and X. Cao, EMBO J

25, 1469 (2006).

[166] H. Wang, T. H. Peiris, A. Mowery, J. Le Lay, Y. Gao, and L. E. Greenbaum, Mol Endocrinol **22**, 1596 (2008).

[167] R. J. Southgate, C. R. Bruce, A. L. Carey, G. R. Steinberg, K. Walder, R. Monks, M. J. Watt, J. A. Hawley, M. J. Birnbaum, and M. A. Febbraio, FASEB J **19**, 2072 (2005).

[168] J. Liang, Y. Yang, X. Zhu, X. Wang, and R. Chen, J Neurosci Res **88**, 1918 (2010).

[169] C. Villeneuve, C. Guilbeau-Frugier, P. Sicard, O. Lairez, C. Ordener, T. Duparc, D. De Paulis, B. Couderc, O. Spreux-Varoquaux, F. Tortosa, *et al.*, Antioxid Redox sign **18**, 5 (2013).

[170] A. Gillich, S. Bao, N. Grabole, K. Hayashi, M. W. Trotter, V. Pasque, E. Magnúsdóttir, and M. A. Surani, Cell Stem Cell **10**, 425 (2012).

[171] C. Yeamans, D. Wang, I. Paz-Priel, B. E. Torbett, D. G. Tenen, and A. D. Friedman, Blood **110**, 3136 (2007).

[172] Z. Hu, X. Gu, K. Baraoidan, V. Ibanez, A. Sharma, S. Kadkol, R. Munker, S. Ackerman, G. Nucifora, and Y. Saunthararajah, Blood **117**, 6498 (2011).

[173] G. Huang, P. Zhang, H. Hirai, S. Elf, X. Yan, Z. Chen, S. Koschmieder, Y. Okuno, T. Dayaram, J. D. Growney, *et al.*, Nat Genet **40**, 51 (2007).

[174] G. Aue, Y. Du, S. M. Cleveland, S. B. Smith, U. P. Davé, D. Liu, M. A. Weniger, J. Y. Metais, N. A. Jenkins, N. G. Copeland, *et al.*, Blood **118**, 4674 (2011).

[175] J. E. Pimanda, I. J. Donaldson, M. F. de Bruijn, S. Kinston, K. Knezevic, L. Huckle, S. Piltz, J.-R. Landry, A. R. Green, D. Tannahill, *et al.*, Proc Natl Acad Sci USA **104**, 840 (2007).

[176] K. E. Elagib and A. N. Goldfarb, Crit Rev Eukaryot Gene Expr **17**, 271 (2007).

[177] Y. Guo, I. Maillard, S. Chakraborti, E. V. Rothenberg, and N. A. Speck, Blood **112**, 480 (2008).

[178] X. Fang, J.-G. Yoon, L. Li, W. Yu, J. Shao, D. Hua, S. Zheng, L. Hood, D. R. Goodlett, G. Foltz, *et al.*, BMC Genomics **12**, 11 (2011).

[179] N. Pencovich, R. Jaschek, A. Tanay, and Y. Groner, Blood **117**, e1 (2011).

[180] M. Iacovino, D. Chong, I. Szatmari, L. Hartweck, D. Rux, A. Caprioli, O. Cleaver, and M. Kyba, Nat Cell Biol **13**, 72 (2011).

[181] S. Ogawa, M. Satake, and K. Ikuta, J Biochem (Tokyo) **143**, 695 (2008).

[182] S. Ogawa, M. Satake, and K. Ikuta, J Biochem (Tokyo) **143**, 695 (2008).

[183] Y. Wang, R. M. Belflower, Y.-F. Dong, E. M. Schwarz, R. J. O'Keefe, and H. Drissi, J Bone Miner Res **20**, 1624 (2005).

[184] N. Wiper-Bergeron, C. St-Louis, and J. M. Lee, Mol Endocrinol **21**, 2124 (2007).

[185] L. Schibler, L. Gibbs, C. Benoist-Lasselin, C. Decraene, J. Martinovic, P. Loget, A.-L. Delezoide, M. Gonzales, A. Munnich, J.-P. Jais, *et al.*, PLoS One **4**, e7633 (2009).

[186] K.-N. Lee, W.-G. Jang, E.-J. Kim, S.-H. Oh, H.-J. Son, S.-H. Kim, R. Franceschi, X.-K. Zhang, S.-E. Lee, and J.-T. Koh, J Biol Chem **287**, 18888 (2012).

[187] F. Engin, T. Bertin, O. Ma, M. M. Jiang, L. Wang, R. E. Sutton, L. A. Donehower, and B. Lee, Hum Mol Genet **18**, 1464 (2009).

[188] J. Lengler, T. Bittner, D. Münster, A.-D. Gawad, and J. Graw, Ophthalmic Res **37**, 301 (2005).

[189] K. Liu, M. Jiang, Y. Lu, H. Chen, J. Sun, S. Wu, W.-Y. Ku, H. Nakagawa, Y. Kita, S. Natsugoe, *et al.*, Cell Stem Cell **12**, 304 (2013).

[190] T. Okubo, L. H. Pevny, and B. L. Hogan, Genes Dev **20**, 2654 (2006).

[191] V. Camilo, R. Barros, S. Sousa, A. M. Magalhães, T. Lopes, A. M. Santos, T. Pereira, C. Figueiredo, L. David, and R. Almeida, Carcinogenesis , bgs233 (2012).

[192] M. Marqués-Torrejón, E. Porlan, A. Banito, E. Gómez-Ibarlucea, A. J. Lopez-Contreras, Ó. Fernández-Capetillo, A. Vidal, J. Gil, J. Torres, and I. Fariñas, Cell Stem Cell **12**, 88 (2013).

[193] M. Saegusa, M. Hashimura, and T. Kuwata, Lab Invest **92**, 511 (2012).

[194] N. Geijsen, I. J. Uings, C. Pals, J. Armstrong, M. McKinnon, J. A. Raaijmakers, J.-W. J. Lammers, L. Koenderman, and P. J. Coffer, Science **293**, 1136 (2001).

[195] S. J. Vervoort, A. R. Lourenço, R. van Boxtel, and P. J. Coffer, PLoS One **8**, e53238 (2013).

[196] C. D. Scharer, C. D. McCabe, M. Ali-Seyed, M. F. Berger, M. L. Bulyk, and C. S. Moreno, Cancer Res **69**, 709 (2009).

[197] H. Zhang, M. Alberich-Jorda, G. Amabile, H. Yang, P. B. Staber, A. DiRuscio, R. S. Welner, A. Ebralidze, J. Zhang, E. Levantini, *et al.*, Cancer Cell **24**, 575 (2013).

[198] K. Harada, A. Ogai, T. Takahashi, M. Kitakaze, H. Matsubara, and H. Oh, J Biol Chem **283**, 26705 (2008).

[199] A. Gandillet, A. G. Serrano, S. Pearson, M. Lie-A-Ling, G. Lacaud, and V. Kouskoff, Blood **114**, 4813 (2009).

[200] X. Zhou, S.-Y. Huang, J.-X. Feng, Y.-Y. Gao, L. Zhao, J. Lu, B.-Q. Huang, and Y. Zhang, World J Gastroenterol **17**, 4922 (2011).

[201] F. Nakaki and M. Saitou, Trends Biochem Sci **39**, 289 (2014).

[202] Z. Huang, H. Xu, and L. Sandell, J Bone Miner Res **19**, 245 (2004).

[203] M.-H. Lee, Y.-J. Kim, H.-J. Kim, H.-D. Park, A.-R. Kang, H.-M. Kyung, J.-H. Sung, J. M. Wozney, and H.-M. Ryoo, J Biol Chem **278**, 34387 (2003).

[204] S. Murakami, M. Kan, W. L. McKeehan, and B. De Crombrugghe, Proc Natl Acad Sci USA **97**, 1113 (2000).

[205] H. P. Shih, J. L. Kopp, M. Sandhu, C. L. Dubois, P. A. Seymour, A. Grapin-Botton, and M. Sander, Development **139**, 2488 (2012).

[206] W. Huang, X. Zhou, V. Lefebvre, and B. de Crombrugghe, Mol Cell Biol **20**, 4149 (2000).

[207] J. Park, J. J. Zhang, A. Moro, M. Kushida, M. Wegner, and P. C. Kim, Dev Dyn **239**, 514 (2010).

[208] T. Furumatsu, M. Tsuda, N. Taniguchi, Y. Tajima, and H. Asahara, J Biol Chem **280**, 8343 (2005).

[209] Y. Kim, A. Kobayashi, R. Sekido, L. DiNapoli, J. Brennan, M.-C. Chaboissier, F. Poulat, R. R. Behringer, R. Lovell-Badge, and B. Capel, PLoS Biol **4**, e187 (2006).

[210] S. Murakami, V. Lefebvre, and B. de Crombrugghe, J Biol Chem **275**, 3687 (2000).

[211] J. S. Rockel, J. C. Kudirka, A. J. Guzi, and S. M. Bernier, Arthritis Res Ther **10**, R3 (2008).

[212] I. Sekiya, P. Koopman, K. Tsuji, S. Mertin, V. Harley, Y. Yamada, K. Shinomiya, A. Niguji, and M. Noda, J Cell Biochem **81**, 71 (2001).

[213] S.-G. Hwang, S.-S. Yu, H. Poo, and J.-S. Chun, J Biol Chem **280**, 29780 (2005).

[214] Z. Wu, E. D. Rosen, R. Brun, S. Hauser, G. Adelmant, A. E. Troy, C. McKeon, G. J. Darlington, and B. M. Spiegelman, Mol Cell **3**, 151 (1999).

[215] F. Oger, J. Dubois-Chevalier, C. Gheeraert, S. Avner, E. Durand, P. Froguel, G. Salbert, B. Staels, P. Lefebvre, and J. Eeckhoute, J Biol Chem **289**, 708 (2014).

[216] F. Marchildon, C. St-Louis, R. Akter, V. Roodman, and N. L. Wiper-Bergeron, J Biol Chem **285**, 13274 (2010).

[217] M. Baik, J. H. Yu, and L. Hennighausen, Ann N Y Acad Sci **1229**, 29 (2011).

[218] H. Xing, J. P. Northrop, J. R. Grove, K. E. Kilpatrick, J.-L. Su, and G. M. Ringold, Endocrinology **138**, 2776 (1997).

[219] W.-Y. Lin, Y.-J. Hu, and Y.-H. Lee, Am J Physiol **295**, G542 (2008).

[220] J. Peltier, J. Perez, A. Bellocq, B. Escoubet, B. Fouqueray, and L. Baud, Kidney Int **63**, 2028 (2003).

[221] M. U. MARTINO, S. ALESCI, G. P. CHROUSOS, and T. KINO, Ann N Y Acad Sci **1024**, 72 (2004).

[222] C. Martens, S. Bilodeau, M. Maira, Y. Gauthier, and J. Drouin, Mol Endocrinol **19**, 885 (2005).

[223] D. Bluteau, L. Gilles, M. Hilpert, I. Antony-Debré, C. James, N. Debili, V. Camara-Clayette, O. Wagner-Ballon, V. Cordette-Lagarde, T. Robert, *et al.*, Blood **118**, 6310 (2011).

[224] D. Liu, H. Jia, D. I. R. Holmes, A. Stannard, and I. Zachary, Arterioscler Thromb Vasc Biol **23**, 2002 (2003).

[225] L. J. Martin and J. J. Tremblay, Biol Reprod **83**, 3 (2010).

[226] L. A. McPherson, G. W. Woodfield, and R. J. Weigel, J Surg Res **138**, 71 (2007).

[227] W. J. Housley, C. A. O'Conor, F. Nichols, L. Puddington, E. G. Lingenheld, L. Zhu, and R. B. Clark, J Leukoc Biol **86**, 293 (2009).

[228] M. A. Antonyak, C. J. McNeill, J. J. Wakshlag, J. E. Boehm, and R. A. Cerione, J Biol Chem **278**, 15859 (2003).

[229] S. Malpel, C. Mendelsohn, and W. V. Cardoso, Development **127**, 3057 (2000).

[230] L. Li, E. Arman, P. Ekblom, D. Edgar, P. Murray, and P. Lonai, Development **131**, 5277 (2004).

[231] J. Fujikura, E. Yamato, S. Yonemura, K. Hosoda, S. Masui, K. Nakao, J.-i. Miyazaki, and H. Niwa, Genes Dev **16**, 784 (2002).

[232] E. More, T. Fellner, H. Doppelmayr, C. Hauser-Kronberger, N. Dandachi, P. Obrist, F. Sandhofer, and B. Paulweber, J Endocrinol **176**, 83 (2003).

[233] Y. Li, Y. Xia, Y. Wang, L. Mao, Y. Gao, Q. He, M. Huang, S. Chen, and B. Hu, Mol Neurobiol **47**, 967 (2013).

[234] D.-E. Zhang, P. Zhang, N.-d. Wang, C. J. Hetherington, G. J. Darlington, and D. G. Tenen, Proc Natl Acad Sci USA **94**, 569 (1997).

[235] S. M. Dunn, L. S. Coles, R. K. Lang, S. Gerondakis, M. A. Vadas, and M. F. Shannon, Blood **83**, 2469 (1994).

[236] L. T. Smith, S. Hohaus, D. Gonzalez, S. Dziennis, and D. Tenen, Blood **88**, 1234 (1996).

[237] R. Newton, Thorax **55**, 603 (2000).

[238] J. Fields, A. Ghorpade, *et al.*, J Neuroinflammation **9**, 1 (2012).

[239] W. E. Paul, ed., *Fundamental Immunology*, 7th ed. (Lippincot Williams and Wilkins, Philadelphia, 2012).

[240] J Clin Invest **107**, 1055 (2001).

[241] Y. Kitagishi, M. Kobayashi, K. Kikuta, and S. Matsuda, Depress Res Treat **2012** (2012).

[242] A. B. Roos and M. Nord, J Endocrinol **212**, 291 (2012).

[243] T. B. van Dijk, B. Baltus, J. A. Raaijmakers, J.-W. J. Lammers, L. Koenderman, and R. P. de Groot, J Immunol **163**, 2674 (1999).

[244] E. W. Baxter, F. Mirabella, S. R. Bowers, S. R. James, A.-M. Bonavita, E. Bertrand, R. Strogantsev, A. Hawwari, A. G. Bert, A. G. de Arce, *et al.*, J Immunol **189**, 4459 (2012).

[245] X. Zhao, V. Jankovic, A. Gural, G. Huang, A. Pardanani, S. Menendez, J. Zhang, R. Dunne, A. Xiao, H. Erdjument-Bromage, *et al.*, Genes Dev **22**, 640 (2008).

[246] S. Nimer, J. Zhang, H. Avraham, and Y. Miyazaki, Blood **88**, 66 (1996).

[247] C. Robin, K. Ottersbach, C. Durand, M. Peeters, L. Vanes, V. Tybulewicz, and E. Dzierzak, Dev Cell **11**, 171 (2006).

[248] G. Xu, Y. Zhang, L. Zhang, A. I. Roberts, and Y. Shi, Stem Cells **27**, 942 (2009).

[249] E. Mahdipour, J. C. Charnock, and K. A. Mace, Blood **117**, 815 (2011).

[250] W. Xiao, D. R. Hodge, L. Wang, X. Yang, X. Zhang, and W. L. Farrar, Cancer Biol Ther **3**, 1007 (2004).

[251] S. J. Lee, E. Borsting, A.-E. Declèves, P. Singh, and R. Cunard, Nephron Exp Nephrol **121**, e86 (2012).

[252] S. Sawatsri, N. Desai, J. A. Rock, and N. Sidell, Fertil Steril **73**, 1012 (2000).

[253] S. Chen, Y. Xu, Y. Chen, X. Li, W. Mou, L. Wang, Y. Liu, R. A. Reisfeld, R. Xiang, D. Lv, *et al.*, PLoS One **7**, e36326 (2012).

[254] M. J. May and S. Ghosh, Immunol Today **19**, 80 (1998).

[255] F. Meng and S. R. D'Mello, Biochim Biophys Acta **1630**, 35 (2003).

[256] B. You, Y.-Y. Jiang, S. Chen, G. Yan, and J. Sun, Circ Res **104**, 742 (2009).

[257] X. Yan, Z. Liu, and Y. Chen, Acta Biochim Biophys Sinica **41**, 263 (2009).

[258] D. Bai, L. Ueno, and P. K. Vogt, Int J Cancer **125**, 2863 (2009).

[259] M. Ohtsubo, A. Takayanagi, S. Gamou, and N. Shimizu, J Cell Physiol **184**, 131 (2000).

[260] E. Dudley, F. Hornung, L. Zheng, D. Scherer, D. Ballard, and M. Lenardo, Eur J Immunol **29**, 878 (1999).

[261] C. Cappello, A. Zwergal, S. Kanclerski, S. C. Haas, J. D. Kandemir, R. Huber, S. Page, and K. Brand, Cell Signal **21**, 1918 (2009).

[262] X. Chen, B. Ji, B. Han, S. A. Ernst, D. Simeone, and C. D. Logsdon, Gastroenterol **122**, 448 (2002).

[263] B. Han and C. D. Logsdon, Am J Physiol **278**, C344 (2000).

[264] L. A. Solt, L. A. Madge, J. S. Orange, and M. J. May, J Biol Chem **282**, 8724 (2007).

[265] H. Mutoh, H. Hayakawa, H. Sakamoto, and K. Sugano, J Gastroenterol **42**, 719 (2007).

[266] J. R. Heyen, S.-m. Ye, B. N. Finck, and R. W. Johnson, Mol Brain Res **77**, 138 (2000).

[267] P. Dandona, A. Aljada, and P. Mohanty, Diabetologia **45**, 924 (2002).

[268] A. Remels, R. Langen, H. R. Gosker, A. Russell, F. Spaapen, J. Voncken, P. Schrauwen, and A. M. Schols, Am J Physiol Endocrinol Metab **297**, E174 (2009).

[269] J. Torres and F. M. Watt, Nat Cell Biol **10**, 194 (2008).

[270] C. Owyang and A. Heldsinger, J Neurogastroenterol Motil **17**, 338 (2011).

[271] B. Kwabi-Addo, M. Ozen, and M. Ittmann, Endocr Relat Cancer **11**, 709 (2004).

[272] E. K. Duperret, S. J. Oh, A. McNeal, S. M. Prouty, and T. W. Ridky, Cell Cycle **13**, 0 (2014).

[273] R. Turkington, D. Longley, W. Allen, L. Stevenson, K. McLaughlin, P. Dunne, J. Blayney, M. Salto-Tellez, S. Van Schaeybroeck, and P. Johnston, Cell Death Differ **5**, e1046 (2014).

[274] S.-S. Tian, P. Tapley, C. Sincich, R. B. Stein, J. Rosen, and P. Lamb, Blood **88**, 4435 (1996).

[275] A. Ferrand, A. Kowalski-Chauvel, C. Bertrand, L. Pradayrol, D. Fourmy, M. Dufresne, and C. Seva, Exp Cell Res **301**, 128 (2004).

[276] M. Berishaj, S. P. Gao, S. Ahmed, K. Leslie, H. Al-Ahmadie, W. L. Gerald, W. Bornmann, J. F. Bromberg, *et al.*, Breast Cancer Res **9**, R32 (2007).

[277] R. Behera, V. Kumar, K. Lohite, S. Karnik, and G. C. Kundu, Carcinogenesis **31**, 192 (2010).

[278] M. Bartoli, D. PLATT, T. Lemtalsi, X. Gu, S. E. Brooks, M. B. Marrero, and R. B. Caldwell, FASEB J **17**, 1562 (2003).

[279] S. Ezoe, I. Matsumura, K. Gale, Y. Satoh, J. Ishikawa, M. Mizuki, S. Takahashi, N. Minegishi, K. Nakajima, M. Yamamoto, *et al.*, J Biol Chem **280**, 13163 (2005).

[280] L. H. Wang, X. Y. Yang, X. Zhang, J. Huang, J. Hou, J. Li, H. Xiong, K. Mihalic, H. Zhu, W. Xiao, *et al.*, Immunity **20**, 205 (2004).

[281] J. Zhou, J. Wulfkuhle, H. Zhang, P. Gu, Y. Yang, J. Deng, J. B. Margolick, L. A. Liotta, E. Petricoin, and Y. Zhang, Proc Natl Acad Sci USA **104**, 16158 (2007).

[282] S. Sun and B. M. Steinberg, J Gen Virol **83**, 1651 (2002).

[283] Z. Syed, S. B. Cheepala, J. N. Gill, J. Stein, C. A. Nathan, J. DiGiovanni, V. Batra, P. Adegboyega, H. E. Kleiner, and J. L. Clifford, Cancer Prev Res **2**, 903 (2009).

[284] J. P. Cerliani, T. Guillardoy, S. Giulianelli, J. P. Vaque, J. S. Gutkind, S. I. Vanzulli, R. Martins, E. Zeitlin, C. A. Lamb, and C. Lanari, Cancer Res **71**, 3720 (2011).

[285] J. Chen, H. B. Sadowski, R. A. Kohanski, and L.-H. Wang, Proc Natl Acad Sci USA **94**, 2295 (1997).

[286] A. x. Dudley, D. Thomas, J. Best, and A. Jenkins, Biochem J **390**, 427 (2005).

[287] E. Cocolakis, M. Dai, L. Drevet, J. Ho, E. Haines, S. Ali, and J.-J. Lebrun, J Biol Chem **283**, 1293 (2008).

[288] C. A. Shah, L. Bei, H. Wang, L. C. Platanias, and E. A. Eklund, J Biol Chem **287**, 18230 (2012).

[289] S. Y. Ko, N. Barengo, A. Ladanyi, J.-S. Lee, F. Marini, E. Lengyel, H. Naora, *et al.*, J Clin Invest **122**, 3603 (2012).

[290] S. A. Vetrone, E. Montecino-Rodriguez, E. Kudryashova, I. Kramerova, E. P. Hoffman, S. D. Liu, M. C. Miceli, M. J. Spencer, *et al.*, J Clin Invest **119**, 1583 (2009).

[291] C. Zhang, T. Basta, and M. W. Klymkowsky, Dev Dyn **234**, 878 (2005).

[292] R.-Y. Liu, Y. Zeng, Z. Lei, L. Wang, H. Yang, Z. Liu, J. Zhao, and H.-T. Zhang, Int J Oncol **44**, 1643 (2014).

[293] J. P. Annes, J. S. Munger, and D. B. Rifkin, J Cell Sci **116**, 217 (2003).

[294] M. Cordenonsi, S. Dupont, S. Maretto, A. Insinga, C. Imbriano, and S. Piccolo, Cell **113**, 301 (2003).

[295] R. Elston and G. J. Inman, J Signal Transduct **2012** (2012).

[296] N. Froese, B. Kattih, A. Breitbart, A. Grund, R. Geffers, J. D. Molkentin, A. Kispert, K. C. Wollert, H. Drexler, and J. Heineke, J Biol Chem **286**, 5680 (2011).

[297] J. Qin, S.-P. Wu, C. J. Creighton, F. Dai, X. Xie, C.-M. Cheng, A. Frolov, G. Ayala, X. Lin, X.-H. Feng, *et al.*, Nature **493**, 236 (2013).

[298] G. Salbert, A. Fanjul, F. J. Piedrafita, X. P. Lu, S.-J. Kim, P. Tran, and M. Pfahl, Mol Endocrinol **7**, 1347 (1993).

[299] D. H. Tompkins, V. Besnard, A. W. Lange, S. E. Wert, A. R. Keiser, A. N. Smith, R. Lang, and J. A. Whitsett, PLoS One **4**, e8248 (2009).

[300] A. Hosui, A. Kimura, D. Yamaji, B.-m. Zhu, R. Na, and L. Hennighausen, J Exp Med **206**, 819 (2009).

[301] E.-L. Alarmo and A. Kallioniemi, Endocr Relat Cancer **17**, R123 (2010).

[302] J. Huang, L. K. Dattilo, R. Rajagopal, Y. Liu, V. Kaartinen, Y. Mishina, C.-X. Deng, L. Umans, A. Zwijsen, A. B. Roberts, *et al.*, Development **136**, 1741 (2009).

[303] L. Rong, J. Liu, Y. Qi, A. M. Graham, M. S. Parmacek, and S. Li, Mol Biol Cell **23**, 3754 (2012).

[304] M. Keita, M. Bachvarova, C. Morin, M. Plante, J. Gregoire, M.-C. Renaud, A. Sebastianelli, X. B. Trinh, and D. Bachvarov, Cell Cycle **12**, 972 (2013).

[305] J. A. Bonzo, C. H. Ferry, T. Matsubara, J.-H. Kim, and F. J. Gonzalez, J Biol Chem **287**, 7345 (2012).

[306] D. A. Lim, A. D. Tramontin, J. M. Trevejo, D. G. Herrera, J. M. García-Verdugo, and A. Alvarez-Buylla, Neuron **28**, 713 (2000).

[307] J. Wang, J. W. Park, H. Drissi, X. Wang, and R.-H. Xu, J Biol Chem **289**, 2384 (2014).

[308] E. Grönroos, I. J. Kingston, A. Ramachandran, R. A. Randall, P. Vizán, and C. S. Hill, Mol Cell Biol **32**, 2904 (2012).

[309] H. L. Ashurst, A. Varro, and R. Dimaline, Exp Physiol **93**, 223 (2008).

[310] C. van de Ven, M. Bialecka, R. Neijts, T. Young, J. E. Rowland, E. J. Stringer, C. Van Rooijen, F. Meijlink, A. Nóvoa, J.-N. Freund, *et al.*, Development **138**, 3451 (2011).

[311] J. C. Harris, P. A. Clarke, A. Awan, J. Jankowski, and S. A. Watson, Cancer Res **64**, 1915 (2004).

[312] E. Beuling, B. E. Aronson, L. M. Tran, K. A. Stapleton, E. N. ter Horst, L. A. Vissers, M. P. Verzi, and S. D. Krasinski, Mol Cell Biol **32**, 3392 (2012).

[313] M. Kurosawa, K. Uvnäs-Moberg, K. Miyasaka, and T. Lundeberg, J Auton Nerv Syst **62**, 72 (1997).

[314] S. Ohgo, K. Nakatsuru, E. Ishikawa, and S. Matsukura, Brain Res **593**, 25 (1992).

[315] J. M. Daun and D. O. McCarthy, Physiol Behav **54**, 237 (1993).

[316] S. Julien, J. Laine, and J. Morisset, Diabetes **53**, 1526 (2004).

[317] L. Gunaratnam, M. Morley, A. Franovic, N. de Paulsen, K. Mekhail, D. A. Parolin, E. Nakamura, I. A. Lorimer, and S. Lee, J Biol Chem **278**, 44966 (2003).

[318] D. F. Stern, A. Roberts, N. Roche, M. Sporn, and R. Weinberg, Mol Cell Biol **6**, 870 (1986).

[319] E.-S. Park, S. Choi, K. N. Muse, T. E. Curry Jr, and M. Jo, Endocrinology **149**, 3025 (2008).

[320] T. R. Fenton, D. Nathanson, C. P. de Albuquerque, D. Kuga, A. Iwanami, J. Dang, H. Yang, K. Tanaka, S. M. Oba-Shinjo, M. Uno, *et al.*, Proc Natl Acad Sci USA **109**, 14164 (2012).

[321] N. Quarto and F. Amalric, J Cell Sci **107**, 3201 (1994).

[322] D. Fraidenraich, A. Iwahori, M. Rudnicki, and C. Basilico, Dev Biol **225**, 392 (2000).

[323] D. Small, D. Kovalenko, R. Soldi, A. Mandinova, V. Kolev, R. Trifonova, C. Bagala, D. Kacer, C. Battelli, L. Liaw, *et al.*, J Biol Chem **278**, 16405 (2003).

[324] C. Palmieri, D. Roberts-Clark, A. Assadi-Sabet, R. Coope, M. O'Hare, A. Sunters, A. Hanby, M. Slade, J. Gomm, E. Lam, *et al.*, J Endocrinol **177**, 65 (2003).

[325] S. Jakob and R. Lovell-Badge, FEBS J **278**, 1002 (2011).

[326] Y. Lu, Y. Li, A. C. Cavender, S. Wang, A. Mansukhani, and R. N. D'Souza, Dev Dyn **241**, 1708 (2012).

[327] M. Yamaji, J. Ueda, K. Hayashi, H. Ohta, Y. Yabuta, K. Kurimoto, R. Nakato, Y. Yamada, K. Shirahige, and M. Saitou, Cell Stem Cell **12**, 368 (2013).

[328] B. P. Jacky, P. E. Garay, J. Dupuy, J. B. Nelson, B. Cai, Y. Molina, J. Wang, L. E. Steward, R. S. Broide, J. Francis, *et al.*, PLoS Pathog **9**, e1003369 (2013).

[329] F. S. Falvella, E. Frullanti, A. Galvan, M. Spinola, S. Noci, L. De Cecco, M. Nosotti, L. Santambrogio, M. Incarbone, M. Alloisio, *et al.*, Int J Cancer **124**, 2880 (2009).

[330] A. Chakladar, A. Dubeykovskiy, L. J. Wojtukiewicz, J. Pratap, S. Lei, and T. C. Wang, Biochem Biophys Res Commun **336**, 190 (2005).

[331] S. Lei, A. Dubeykovskiy, A. Chakladar, L. Wojtukiewicz, and T. C. Wang, J Biol Chem **279**, 42492 (2004).

[332] S. Mathas, M. Hinz, I. Anagnostopoulos, D. Krappmann, A. Lietz, F. Jundt, K. Bommert, F. Mechta-Grigoriou, H. Stein, B. Dörken, *et al.*, EMBO J **21**, 4104 (2002).

[333] K. Matsumoto, H. Tajima, H. Okazaki, and T. Nakamura, J Biol Chem **267**, 24917 (1992).

[334] A. Leonardini, L. Laviola, S. Perrini, A. Natalicchio, and F. Giorgino, PPAR Res **2009**

(2010).

[335] S. R. Parathath, L. A. Mainwaring, A. Fernandez-L, D. O. Campbell, and A. M. Kenney, Development **135**, 3291 (2008).

[336] M. Abiola, M. Favier, E. Christodoulou-Vafeiadou, A.-L. Pichard, I. Martelly, and I. Guillet-Deniau, PLoS One **4**, e8509 (2009).

[337] U. B. Pajvani, C. J. Shawber, V. T. Samuel, A. L. Birkenfeld, G. I. Shulman, J. Kitajewski, and D. Accili, Nat Med **17**, 961 (2011).

[338] L. Rui, V. Aguirre, J. K. Kim, G. I. Shulman, A. Lee, A. Corbould, A. Dunaif, M. F. White, *et al.*, J Clin Invest **107**, 181 (2001).

[339] J. Heineke, M. Auger-Messier, J. Xu, T. Oka, M. A. Sargent, A. York, R. Klevitsky, S. Vaikunth, S. A. Duncan, B. J. Aronow, *et al.*, J Clin Invest **117**, 3198 (2007).

[340] E. B. Rankin, J. Rha, T. L. Unger, C. H. Wu, H. P. Shutt, R. S. Johnson, M. C. Simon, B. Keith, and V. H. Haase, Oncogene **27**, 5354 (2008).

[341] F. A. Pereira, Y. Qiu, G. Zhou, M.-J. Tsai, and S. Y. Tsai, Genes Dev **13**, 1037 (1999).

[342] H. Zeng, L. Qin, D. Zhao, X. Tan, E. J. Manseau, M. Van Hoang, D. R. Senger, L. F. Brown, J. A. Nagy, and H. F. Dvorak, J Exp Med **203**, 719 (2006).

[343] Z. Arany, S.-Y. Foo, Y. Ma, J. L. Ruas, A. Bommi-Reddy, G. Girnun, M. Cooper, D. Laznik, J. Chinsomboon, S. M. Rangwala, *et al.*, Nature **451**, 1008 (2008).

[344] J. E. Jung, H. G. Lee, I. H. Cho, D. H. Chung, S.-H. Yoon, Y. M. Yang, J. W. Lee, S. Choi, J.-W. Park, S.-K. Ye, *et al.*, FASEB J **19**, 1296 (2005).

[345] G. Niu, K. L. Wright, M. Huang, L. Song, E. Haura, J. Turkson, S. Zhang, T. Wang, D. Sinibaldi, D. Coppola, *et al.*, Oncogene **21**, 2000 (2002).

[346] Q. Xu, J. Briggs, S. Park, G. Niu, M. Kortylewski, S. Zhang, T. Gritsko, J. Turkson, H. Kay, G. L. Semenza, *et al.*, Oncogene **24**, 5552 (2005).

[347] C. N. Nagineni, W. Samuel, S. Nagineni, K. Pardhasaradhi, B. Wiggert, B. Detrick, and J. J. Hooks, J Cell Physiol **197**, 453 (2003).

[348] G. Ferrari, B. D. Cook, V. Terushkin, G. Pintucci, and P. Mignatti, J Cell Physiol **219**, 449 (2009).

[349] O. Watson, P. Novodvorsky, C. Gray, A. M. Rothman, A. Lawrie, D. C. Crossman, A. Haase, K. McMahon, M. Gering, F. J. Van Eeden, *et al.*, Cardiovasc Res **100**, 252 (2013).

[350] N. Sidell, Y. Feng, L. Hao, J. Wu, J. Yu, M. A. Kane, J. L. Napoli, and R. N. Taylor, Mol

Endocrinol **24**, 148 (2010).

[351] H. Hirai, I. M. Samokhvalov, T. Fujimoto, S. Nishikawa, J. Imanishi, and S.-I. Nishikawa, Blood **106**, 1948 (2005).

[352] T. Hattori, C. Müller, S. Gebhard, E. Bauer, F. Pausch, B. Schlund, M. R. Bösl, A. Hess, C. Surmann-Schmitt, H. von der Mark, *et al.*, Development **137**, 901 (2010).

[353] L. Zhou, H. Yang, E. U. Okoro, and Z. Guo, J Biol Chem **289**, 12989 (2014).

[354] T. Galbaugh, M. G. Cerrito, C. C. Jose, and M. L. Cutler, BMC Cell Biol **7**, 34 (2006).

[355] K. Siddle, J Mol Endocrinol **47**, R1 (2011).

[356] T. Palomero, M. L. Sulis, M. Cortina, P. J. Real, K. Barnes, M. Ciofani, E. Caparros, J. Buteau, K. Brown, S. L. Perkins, *et al.*, Nat Med **13**, 1203 (2007).

[357] J. T. Stuenæs, A. Bolling, A. Ingvaldsen, C. Rommundstad, E. Sudar, F.-C. Lin, Y.-C. Lai, and J. Jensen, Br J Pharmacol **160**, 116 (2010).

[358] J. Bastien, J. Plassat, B. Payrastre, and C. Rochette-Egly, Oncogene **25**, 2040 (2005).

[359] E. Castellano and J. Downward, Genes Cancer **2**, 261 (2011).

[360] Z. Shao, K. Bhattacharya, E. Hsich, L. Park, B. Walters, U. Germann, Y.-M. Wang, J. Kyriakis, R. Mohanlal, K. Kuida, *et al.*, Circ Res **98**, 111 (2006).

[361] K. A. Waite and C. Eng, Am J Hum Genet **70**, 829 (2002).

[362] E. V. Maytin, J. C. Lin, R. Krishnamurthy, N. Batchvarova, D. Ron, P. J. Mitchell, and J. F. Habener, Dev Biol **216**, 164 (1999).

[363] M. A. Hubert, S. L. Sherritt, C. J. Bachurski, and S. Handwerger, PLoS One **5**, e9417 (2010).

[364] B. Lüscher, P. J. Mitchell, T. Williams, and R. Tjian, Genes Dev **3**, 1507 (1989).

[365] Y.-M. Go, Y. C. Boo, H. Park, M. C. Maland, R. Patel, K. A. Pritchard, Y. Fujio, K. Walsh, V. Darley-Usmar, and H. Jo, J Appl Physiol **91**, 1574 (2001).

[366] L. Guo, M. D. Sans, Y. Hou, S. A. Ernst, and J. A. Williams, Am J Physiol **302**, G1381 (2012).

[367] R. Kajanne, P. Miettinen, A. Mehlem, S.-K. Leivonen, M. Birrer, M. Foschi, V.-M. Kähäri, and S. Leppä, J Cell Physiol **212**, 489 (2007).

[368] A. Mukhopadhyay, S. Shishodia, X.-Y. Fu, and B. B. Aggarwal, J Cell Biochem **84**, 803 (2002).

[369] J. Rangatia, R. K. Vangala, N. Treiber, P. Zhang, H. Radomska, D. G. Tenen, W. Hiddemann,

and G. Behre, Mol Cell Biol **22**, 8681 (2002).

[370] J.-J. Ventura, C. Roncero, I. Fabregat, and M. Benito, Hepatology **29**, 849 (1999).

[371] P. R. Mittelstadt and J. D. Ashwell, J Biol Chem **276**, 29603 (2001).

[372] M. Al-Mutairi, S. Al-Harthi, L. Cadalbert, and R. Plevin, Br J Pharmacol **161**, 782 (2010).

[373] G. L. Semenza, Cancer Metastasis Rev **19**, 59 (2000).

[374] B.-Y. Kim, H. Kim, E.-J. Cho, and H.-D. Youn, Exp Mol Med **40**, 71 (2008).

[375] A. Lonic, E. F. Barry, C. Quach, B. Kobe, N. Saunders, and M. A. Guthridge, Mol Cell Biol **28**, 3372 (2008).

[376] S. Murakami, G. Balmes, S. McKinney, Z. Zhang, D. Givol, and B. de Crombrugghe, Genes Dev **18**, 290 (2004).

[377] B. P. Eliceiri, Circ Res **89**, 1104 (2001).

[378] J. Gu, M. Tamura, and K. M. Yamada, J Cell Biol **143**, 1375 (1998).

[379] O. S. Gardner, B. J. Dewar, and L. M. Graves, Mol Pharmacol **68**, 933 (2005).

[380] M. K. Lee, C. Pardoux, M. C. Hall, P. S. Lee, D. Warburton, J. Qing, S. M. Smith, and R. Derynck, EMBO J **26**, 3957 (2007).

[381] H. Wang, Y. Lu, W. Huang, E. T. Papoutsakis, P. Fuhrken, and E. A. Eklund, J Biol Chem **282**, 16164 (2007).

[382] A. Bellis, D. Castaldo, V. Trimarco, M. G. Monti, P. Chivasso, J. Sadoshima, B. Trimarco, and C. Morisco, Arterioscler Thromb Vasc Biol **29**, 1207 (2009).

[383] G. Tortora, V. Damiano, C. Bianco, G. Baldassarre, A. R. Bianco, L. Lanfrancone, P. G. Pelicci, and F. Ciardiello, Oncogene **14**, 923 (1997).

[384] H. Yang, G. Li, J.-J. Wu, L. Wang, M. Uhler, and D. M. Simeone, J Biol Chem **288**, 8737 (2013).

[385] L. Zhang, C. J. Duan, C. Binkley, G. Li, M. D. Uhler, C. D. Logsdon, and D. M. Simeone, Mol Cell Biol **24**, 2169 (2004).

[386] O. V. Leontieva, A. V. Gudkov, and M. V. Blagosklonny, Cell Cycle **9**, 4323 (2010).

[387] H.-J. Choi, T.-W. Chung, S.-J. Kim, S.-Y. Cho, Y.-S. Lee, Y.-C. Lee, J.-H. Ko, and C.-H. Kim, Glycobiology **18**, 395 (2008).

[388] S. Y. Lee, G. Y. Hur, K. H. Jung, H. C. Jung, S. Y. Lee, J. H. Kim, C. Shin, J. J. Shim, K. H. In, K. H. Kang, *et al.*, Lung Cancer **51**, 297 (2006).

[389] Z. Chen, L. C. Trotman, D. Shaffer, H.-K. Lin, Z. A. Dotan, M. Niki, J. A. Koutcher, H. I.

Scher, T. Ludwig, W. Gerald, *et al.*, Nature **436**, 725 (2005).

[390] K. M. Vasudevan, S. Gurumurthy, and V. M. Rangnekar, Mol Cell Biol **24**, 1007 (2004).

[391] L. A. McPherson, A. V. Loktev, and R. J. Weigel, J Biol Chem **277**, 45028 (2002).

[392] D. Bonofiglio, E. Cione, H. Qi, A. Pingitore, M. Perri, S. Catalano, D. Vizza, M. L. Panno, G. Genchi, S. A. Fuqua, *et al.*, Am J Pathol **175**, 1270 (2009).

[393] V. Stambolic, D. MacPherson, D. Sas, Y. Lin, B. Snow, Y. Jang, S. Benchimol, and T. Mak, Mol Cell **8**, 317 (2001).

[394] J. C. Curtin, K. H. Dragnev, D. Sekula, A. J. Christie, E. Dmitrovsky, and M. J. Spinella, Oncogene **20**, 2559 (2001).

[395] L. J. Beverly, D. W. Felsher, and A. J. Capobianco, Cancer Res **65**, 7159 (2005).

[396] X. Pan, J. Zhao, W.-N. Zhang, H.-Y. Li, R. Mu, T. Zhou, H.-Y. Zhang, W.-L. Gong, M. Yu, J.-H. Man, *et al.*, Proc Natl Acad Sci USA **106**, 3788 (2009).

[397] B. Schwartz, V. Melnikova, C. Tellez, A. Mourad-Zeidan, K. Blehm, Y. Zhao, M. McCarty, L. Adam, and M. Bar-Eli, Oncogene **26**, 4049 (2007).

[398] S. Funakoshi, J. Kong, M. A. Crissey, L. Dang, D. Dang, and J. P. Lynch, Am J Physiol **299**, G1054 (2010).

[399] Y. Song, M. K. Washington, and H. C. Crawford, Cancer Res **70**, 2115 (2010).

[400] G. F. Späth and M. C. Weiss, J Cell Biol **140**, 935 (1998).

[401] M.-T. Lau, W.-K. So, and P. C. Leung, PLoS One **8**, e59083 (2013).

[402] J. Yan, N. Wong, C. Hung, W. X.-Y. Chen, and D. Tang, PLoS One **8**, e65463 (2013).

[403] R. B. Hazan and L. Norton, J Biol Chem **273**, 9078 (1998).

[404] S. Jing, Y. Wang, L.-Q. Chen, M. Sang, M. Zheng, G. Sun, Q. Liu, Y. Cheng, and C. Yang, Diseases of the Esophagus **26**, 75 (2013).

[405] B. Krishnamachary, D. Zagzag, H. Nagasawa, K. Rainey, H. Okuyama, J. H. Baek, and G. L. Semenza, Cancer Res **66**, 2725 (2006).

[406] X. Li, W. Deng, S. Lobo-Ruppert, and J. Ruppert, Oncogene **26**, 4489 (2007).

[407] H. Xiong, J. Hong, W. Du, Y.-w. Lin, L.-l. Ren, Y.-c. Wang, W.-y. Su, J.-l. Wang, Y. Cui, Z.-h. Wang, *et al.*, J Biol Chem **287**, 5819 (2012).

[408] R. Vogelmann, M.-D. Nguyen-tat, K. Giehl, G. Adler, D. Wedlich, and A. Menke, J Cell Sci **118**, 4901 (2005).

[409] J. Y. Yi, Y.-J. Jung, S. S. Choi, and E. Chung, Cancer Res Treat **41**, 164 (2009).

[410] N. M. Teplyuk, L. M. Haupt, L. Ling, C. Dombrowski, F. K. Mun, S. S. Nathan, J. B. Lian, J. L. Stein, G. S. Stein, S. M. Cool, *et al.*, J Cell Biochem **107**, 144 (2009).

[411] V. Kolm, U. Sauer, B. Olgemooller, and E. Schleicher, Am J Physiol Renal Physiol **270**, F812 (1996).

[412] D. Woods, H. Cherwinski, E. Venetsanakos, A. Bhat, S. Gysin, M. Humbert, P. F. Bray, V. L. Saylor, and M. McMahon, Mol Cell Biol **21**, 3192 (2001).

[413] S. Nakao, T. Kuwano, T. Ishibashi, M. Kuwano, and M. Ono, J Immunol **170**, 5704 (2003).

[414] A. Wilson, M. J. Murphy, T. Oskarsson, K. Kaloulis, M. D. Bettess, G. M. Oser, A.-C. Pasche, C. Knabenhans, H. R. MacDonald, and A. Trumpp, Genes Dev **18**, 2747 (2004).

[415] P. E. Hughes, M. W. Renshaw, M. Pfaff, J. Forsyth, V. M. Keivens, M. A. Schwartz, and M. H. Ginsberg, Cell **88**, 521 (1997).

[416] C. Fournier-Thibault, C. Blavet, A. Jarov, F. Bajanca, S. Thorsteinsdóttir, and J.-L. Duband, J Neurosci **29**, 12506 (2009).

[417] S. Grainger, J. Lam, J. G. Savory, A. J. Mears, F. M. Rijli, and D. Lohnes, Dev Biol **361**, 1 (2012).

[418] T. Uesaka, N. Kageyama, and H. Watanabe, J Mol Biol **337**, 647 (2004).

[419] B. G. Rash, H. D. Lim, J. J. Breunig, and F. M. Vaccarino, J Neurosci **31**, 15604 (2011).

[420] I. Tremblay, E. Paré, D. Arsenault, M. Douziech, and M.-J. Boucher, PLoS One **8**, e85502 (2013).

[421] L. Espinosa, S. Cathelin, T. D'Altri, T. Trimarchi, A. Statnikov, J. Guiu, V. Rodilla, J. Inglés-Esteve, J. Nomdedeu, B. Bellosillo, *et al.*, Cancer Cell **18**, 268 (2010).

[422] T. Yoshimatsu, D. Kawaguchi, K. Oishi, K. Takeda, S. Akira, N. Masuyama, and Y. Gotoh, Development **133**, 2553 (2006).

[423] T. A. Mitsiadis, D. Graf, H. Luder, T. Gridley, and G. Bluteau, Development **137**, 3025 (2010).

[424] Y.-C. Shi, H. Zhao, C. Yin, X. Zeng, Q. Zhang, W.-P. Xu, J. Wei, F. Chen, and W.-F. Xie, Digestive and Liver Disease **45**, 844 (2013).

[425] A. C. Ferreira, G. Suriano, N. Mendes, B. Gomes, X. Wen, F. Carneiro, R. Seruca, and J. C. Machado, Hum Mol Genet , ddr469 (2011).

[426] O. Boucherat, J. Chakir, and L. Jeannotte, Biol Open , BIO20121701 (2012).

[427] X. Chen, J. Qin, C.-M. Cheng, M.-J. Tsai, and S. Y. Tsai, Mol Endocrinol **26**, 1268 (2012).

[428] X. Wang, C. Blagden, J. Fan, S. J. Nowak, I. Taniuchi, D. R. Littman, and S. J. Burden, Genes Dev **19**, 1715 (2005).

[429] P. Zhao and J. M. Stephens, Mol Metab **2**, 161 (2013).

[430] E. Hirsinger, D. Duprez, C. Jouve, P. Malapert, J. Cooke, and O. Pourquié, Development **124**, 4605 (1997).

[431] B. K. Zehentner, A. Haussmann, and H. Burtscher, Dev Growth Differ **44**, 1 (2002).

[432] M. I. Reinhold, M. Abe, R. M. Kapadia, Z. Liao, and M. C. Naski, J Biol Chem **279**, 38209 (2004).

[433] C. Neumann, F. Garreis, F. Paulsen, C. M. Hammer, M. T. Birke, and M. Scholz, PLoS One **9**, e92762 (2014).

[434] T. Nakamachi, T. Nomiyama, F. Gizard, E. B. Heywood, K. L. Jones, Y. Zhao, L. Fuentes, K. Takebayashi, Y. Aso, B. Staels, *et al.*, Diabetes **56**, 1662 (2007).

[435] A. Mazzucotelli, N. Viguerie, C. Tiraby, J.-S. Annicotte, A. Mairal, E. Klimcakova, E. Lepin, P. Delmar, S. Dejean, G. Tavernier, *et al.*, Diabetes **56**, 2467 (2007).

[436] S. Ali, A. Ahmad, S. Gadgeel, F. H. Sarkar, *et al.*, PLoS One **6**, e16068 (2011).

[437] I. Rios, R. Alvarez-Rodríguez, E. Martí, and S. Pons, Development **131**, 3159 (2004).

[438] M. F. Bastida, R. Sheth, and M. A. Ros, Development **136**, 3779 (2009).

[439] B. Spencer-Dene, F. G. Sala, S. Bellusci, S. Gschmeissner, G. Stamp, and C. Dickson, Gastroenterol **130**, 1233 (2006).

[440] K. C. Schreck, P. Taylor, L. Marchionni, V. Gopalakrishnan, E. E. Bar, N. Gaiano, and C. G. Eberhart, Clin Cancer Res **16**, 6060 (2010).

[441] P. Krejci, A. Aklian, M. Kaucka, E. Sevcikova, J. Prochazkova, J. K. Masek, P. Mikolka, T. Pospisilova, T. Spoustova, M. Weis, *et al.*, PLoS One **7**, e35826 (2012).

[442] A. Alexandrovich, M. Arno, R. K. Patient, A. M. Shah, J. A. Pizzey, and A. C. Brewer, Mech Dev **123**, 297 (2006).

[443] S. P. Monga, W. M. Mars, P. Pediaditakis, A. Bell, K. Mulé, W. C. Bowen, X. Wang, R. Zarnegar, and G. K. Michalopoulos, Cancer Res **62**, 2064 (2002).

[444] F. M. Koraishy, C. Silva, S. Mason, D. Wu, and L. G. Cantley, J Biol Chem **289**, 14341 (2014).

[445] Y. Geng, Y. Ju, F. Ren, Y. Qiu, Y. Tomita, M. Tomoeda, M. Kishida, Y. Wang, L. Jin, F. Su, *et al.*, J Biol Chem **289**, 11230 (2014).

[446] K. L. Auld, S. P. Berasi, Y. Liu, M. Cain, Y. Zhang, C. Huard, S. Fukayama, J. Zhang, S. Choe, W. Zhong, *et al.*, J Mol Endocrinol **48**, 177 (2012).

[447] A. Akhmetshina, K. Palumbo, C. Dees, C. Bergmann, P. Venalis, P. Zerr, A. Horn, T. Kireva, C. Beyer, J. Zwerina, *et al.*, Nat Commun **3**, 735 (2012).

[448] X. Liu, X. Zhang, Q. Zhan, M. V. Brock, J. G. Herman, and M. Guo, Cancer Biol Ther **13**, 1152 (2012).

[449] R.-J. Guo, S. Funakoshi, H. H. Lee, J. Kong, and J. P. Lynch, Carcinogenesis **31**, 159 (2010).

[450] M. Yang, S.-N. Li, K. M. Anjum, L.-X. Gui, S.-S. Zhu, J. Liu, J.-K. Chen, Q.-F. Liu, G.-D. Ye, W.-J. Wang, *et al.*, J Cell Sci **126**, 5692 (2013).

[451] B.-F. Ning, J. Ding, C. Yin, W. Zhong, K. Wu, X. Zeng, W. Yang, Y.-X. Chen, J.-P. Zhang, X. Zhang, *et al.*, Cancer Res **70**, 7640 (2010).

[452] L. Guo, D. Zhong, S. Lau, X. Liu, X.-Y. Dong, X. Sun, V. W. Yang, P. M. Vertino, C. S. Moreno, V. Varma, *et al.*, Mol Cancer Res **6**, 1421 (2008).

[453] D. W. Chan, C. S. Mak, T. H. Leung, K. K. Chan, and H. Y. Ngan, Oncotarget **3**, 1546 (2012).

[454] L. Topol, W. Chen, H. Song, T. F. Day, and Y. Yang, J Biol Chem **284**, 3323 (2009).

II. NETWORK MODELING

A. Modeling the network dynamics by mathematical equations

We use nonlinear Hill type function to implement coarse-grained activation and inhibition between different agents. The dynamical equation for the concentration/activities of agents $\mathbf{x} = (x_1, x_2, \ldots, x_m)$ under the influence of the other agents takes the following form:

$$\frac{d\mathbf{x}}{dt} = \mathbf{f}(\mathbf{x}) - \frac{\mathbf{x}}{\tau} \ . \tag{1}$$

This expression is consistent with chemical rate equations. A main simplification is that the natural protein degradation time is set to be unity for all proteins: $\tau = 1$. If the agent x_2 activates agent x_1, function $f_1(x_2)$ is assigned as the form

$$f_1(x_2) = \frac{a \cdot x_2^n}{1 + a \cdot x_2^n} \ , \tag{2}$$

while for the inhibition on x_1 by x_2

$$f_1(x_2) = \frac{1}{1 + a \cdot x_2^n} \ . \tag{3}$$

If there are multiple activations x_2, x_3 and inhibitions x_4, x_5 to x_1, we have

$$f_1(x_2, \ldots, x_5) = \frac{a \cdot (x_2^n + x_3^n)}{1 + a \cdot (x_2^n + x_3^n)} \times \frac{1}{1 + a \cdot (x_4^n + x_5^n)} \ . \tag{4}$$

The values for each agent is normalized from zero to one. One may notice that our equations guarantee that the values of agents will not go beyond the internal from zero to one while evolving with time. The values for the parameters are typically chosen to be $a = 10$ and $n = 3$. We demonstrate in the following that our results are robust under random parameter tests.

1. *A full list of equations for the network dynamics*

We list below all equations for the network dynamics described in Table S2:

$$\frac{dx_{Cyclin\ D/Cdk4,6}}{dt} = \frac{a \cdot (x_{E2F}^n + x_{Myc}^n + x_{Pu.1}^n + x_{EGFR}^n)}{1 + a \cdot (x_{E2F}^n + x_{Myc}^n + x_{Pu.1}^n + x_{EGFR}^n)} \times \frac{1}{1 + a \cdot (x_{p21}^n + x_{p53}^n)} - x_{Cyclin\ D/Cdk4,6}$$

$$\frac{dx_{Cyclin\ E/Cdk2}}{dt} = \frac{a\cdot(x^n_{E2F}+x^n_{Myc})}{1+a\cdot(x^n_{E2F}+x^n_{Myc})} \times \frac{1}{1+a\cdot(x^n_{p21}+x^n_{p27}+x^n_{PTEN})} - x_{Cyclin\ E/Cdk2}$$

$$\frac{dx_{E2F}}{dt} = \frac{a\cdot(x^n_{E2F}+x^n_{Myc})}{1+a\cdot(x^n_{E2F}+x^n_{Myc})} \times \frac{1}{1+a\cdot(x^n_{p21})} - x_{E2F}$$

$$\frac{dx_{Myc}}{dt} = \frac{a\cdot(x^n_{E2F}+x^n_{pRb}+x^n_{MAPK}+x^n_{Notch}+x^n_{SHH}+x^n_{Wnt})}{1+a\cdot(x^n_{E2F}+x^n_{pRb}+x^n_{MAPK}+x^n_{Notch}+x^n_{SHH}+x^n_{Wnt})} \times \frac{1}{1+a\cdot(x^n_{TGF-\beta}+x^n_{AP2}+x^n_{p53})} - x_{Myc}$$

$$\frac{dx_{p21}}{dt} = \frac{a\cdot(x^n_{Gata1/2}+x^n_{HNF4\alpha}+x^n_{Runx1}+x^n_{GR}+x^n_{TGF-\beta}+x^n_{TNF-\alpha}+x^n_{AP2}+x^n_{p53})}{1+a\cdot(x^n_{Gata1/2}+x^n_{HNF4\alpha}+x^n_{Runx1}+x^n_{GR}+x^n_{TGF-\beta}+x^n_{TNF-\alpha}+x^n_{AP2}+x^n_{p53})} \times \frac{1}{1+a\cdot(x^n_{Myc}+x^n_{Hes1}+x^n_{Akt})} - x_{p21}$$

$$\frac{dx_{p27}}{dt} = \frac{a\cdot(x^n_{Gata1/2}+x^n_{NR4A}+x^n_{TGF-\beta}+x^n_{PTEN}+x^n_{E-Cadherin})}{1+a\cdot(x^n_{Gata1/2}+x^n_{NR4A}+x^n_{TGF-\beta}+x^n_{PTEN}+x^n_{E-Cadherin})} \times \frac{1}{1+a\cdot(x^n_{Myc}+x^n_{Hes1}+x^n_{Akt})} - x_{p27}$$

$$\frac{dx_{pRb}}{dt} = \frac{a\cdot(x^n_{Cyclin\ D/Cdk4,6}+x^n_{Cyclin\ E/Cdk2})}{1+a\cdot(x^n_{Cyclin\ D/Cdk4,6}+x^n_{Cyclin\ E/Cdk2})} - x_{pRb}$$

$$\frac{dx_{Ras}}{dt} = \frac{a\cdot(x^n_{IL-6}+x^n_{EGFR}+x^n_{FGFR2}+x^n_{HGF}+x^n_{IR}+x^n_{VEGF})}{1+a\cdot(x^n_{IL-6}+x^n_{EGFR}+x^n_{FGFR2}+x^n_{HGF}+x^n_{IR}+x^n_{VEGF})} \times \frac{1}{1+a\cdot(x^n_{GR}+x^n_{p53})} - x_{Ras}$$

$$\frac{dx_{Bad}}{dt} = \frac{a\cdot(x^n_{TNF-\alpha}+x^n_{p53})}{1+a\cdot(x^n_{TNF-\alpha}+x^n_{p53})} \times \frac{1}{1+a\cdot(x^n_{p21}+x^n_{NF-\kappa B}+x^n_{Akt}+x^n_{MAPK})} - x_{Bad}$$

$$\frac{dx_{Bax}}{dt} = \frac{a\cdot(x^n_{Bim}+x^n_{p53})}{1+a\cdot(x^n_{Bim}+x^n_{p53})} \times \frac{1}{1+a\cdot(x^n_{Bcl-2}+x^n_{Sox4}+x^n_{Akt})} - x_{Bax}$$

$$\frac{dx_{Bcl-2}}{dt} = \frac{a\cdot(x^n_{NF-\kappa B}+x^n_{VEGF}+x^n_{Integrin})}{1+a\cdot(x^n_{NF-\kappa B}+x^n_{VEGF}+x^n_{Integrin})} \times \frac{1}{1+a\cdot(x^n_{Caspase\ 3}+x^n_{TGF-\beta}+x^n_{p53})} - x_{Bcl-2}$$

$$\frac{dx_{Bcl-xL}}{dt} = \frac{a\cdot(x^n_{NF-\kappa B}+x^n_{Stat5}+x^n_{EGFR})}{1+a\cdot(x^n_{NF-\kappa B}+x^n_{Stat5}+x^n_{EGFR})} \times \frac{1}{1+a\cdot(x^n_{Caspase\ 3})} - x_{Bcl-xL}$$

$$\frac{dx_{Bim}}{dt} = \frac{1}{1+a\times(x^n_{Akt}+x^n_{MAPK})} - x_{Bim}$$

$$\frac{dx_{Caspase\ 3}}{dt} = \frac{a\cdot(x^n_{Bad}+x^n_{Cytochrome\ C}+x^n_{Fas}+x^n_{TNF-\alpha})}{1+a\cdot(x^n_{Bad}+x^n_{Cytochrome\ C}+x^n_{Fas}+x^n_{TNF-\alpha})} \times \frac{1}{1+a\cdot(x^n_{XIAP}+x^n_{NF-\kappa B})} - x_{Caspase\ 3}$$

$$\frac{dx_{Cytochrome\ C}}{dt} = \frac{a\cdot(x^n_{Bad}+x^n_{Bax}+x^n_{Caspase\ 3})}{1+a\cdot(x^n_{Bad}+x^n_{Bax}+x^n_{Caspase\ 3})} \times \frac{1}{1+a\cdot(x^n_{p21}+x^n_{Bcl-2}+x^n_{Bcl-xL})} - x_{Cytochrome\ C}$$

$$\frac{dx_{Fas}}{dt} = \frac{a\cdot(x^n_{TNF-\alpha})}{1+a\cdot(x^n_{TNF-\alpha})} \times \frac{1}{1+a\cdot(x^n_{Ras})} - x_{Fas}$$

$$\frac{dx_{XIAP}}{dt} = \frac{a\cdot(x^n_{RARs}+x^n_{NF-\kappa B}+x^n_{Akt}+x^n_{MAPK}+x^n_{Integrin})}{1+a\cdot(x^n_{RARs}+x^n_{NF-\kappa B}+x^n_{Akt}+x^n_{MAPK}+x^n_{Integrin})} \times \frac{1}{1+a\cdot(x^n_{Caspase\ 3}+x^n_{Cytochrome\ C})} - x_{XIAP}$$

$$\frac{dx_{C/EBP\alpha}}{dt} = \frac{a\cdot(x^n_{C/EBP\beta}+x^n_{HNF4\alpha}+x^n_{Runx1}+x^n_{NOG})}{1+a\cdot(x^n_{C/EBP\beta}+x^n_{HNF4\alpha}+x^n_{Runx1}+x^n_{NOG})} \times \frac{1}{1+a\cdot(x^n_{NR2F2}+x^n_{IL-1}+x^n_{Stat5}+x^n_{HIF}+x^n_{MAPK}+x^n_{Notch}+x^n_{SHH})} - x_{C/EBP\alpha}$$

$$\frac{dx_{C/EBP\beta}}{dt} = \frac{a\cdot(x^n_{IL-1}+x^n_{AP2}+x^n_{MAPK})}{1+a\cdot(x^n_{IL-1}+x^n_{AP2}+x^n_{MAPK})} \times \frac{1}{1+a\cdot(x^n_{Hoxa3}+x^n_{TGF-\beta})} - x_{C/EBP\beta}$$

$$\frac{dx_{Cdx2}}{dt} = \frac{a\cdot(x^n_{Gata1/2}+x^n_{HNF1}+x^n_{HNF4\alpha}+x^n_{Sox2}+x^n_{BMP}+x^n_{c-Jun}+x^n_{MAPK}+x^n_{Wnt})}{1+a\cdot(x^n_{Gata1/2}+x^n_{HNF1}+x^n_{HNF4\alpha}+x^n_{Sox2}+x^n_{BMP}+x^n_{c-Jun}+x^n_{MAPK}+x^n_{Wnt})} \times \frac{1}{1+a\cdot(x^n_{C/EBP\beta}+x^n_{Sox9}+x^n_{Stat3}+x^n_{TGF-\beta})} - x_{Cdx2}$$

$$\frac{dx_{Foxa1}}{dt} = \frac{a\cdot(x^n_{Foxa2}+x^n_{RARs}+x^n_{AP2})}{1+a\cdot(x^n_{Foxa2}+x^n_{RARs}+x^n_{AP2})} \times \frac{1}{1+a\cdot(x^n_{Sox4})} - x_{Foxa1}$$

$$\frac{dx_{Foxa2}}{dt} = \frac{a\cdot(x^n_{C/EBP\alpha}+x^n_{RARs}+x^n_{SHH})}{1+a\cdot(x^n_{C/EBP\alpha}+x^n_{RARs}+x^n_{SHH})} \times \frac{1}{1+a\cdot(x^n_{PRDM14}+x^n_{Akt})} - x_{Foxa2}$$

$$\frac{dx_{Gata1/2}}{dt} = \frac{a\cdot(x^n_{Cdx2}+x^n_{Stat3}+x^n_{Notch})}{1+a\cdot(x^n_{Cdx2}+x^n_{Stat3}+x^n_{Notch})} \times \frac{1}{1+a\cdot(x^n_{Hes1}+x^n_{Hoxa10}+x^n_{Pu.1}+x^n_{VEGF}+x^n_{c-Jun})} - x_{Gata1/2}$$

$$\frac{dx_{Gata4/6}}{dt} = \frac{a\cdot(x^n_{Sox7}+x^n_{PKA}+x^n_{Wnt})}{1+a\cdot(x^n_{Sox7}+x^n_{PKA}+x^n_{Wnt})} \times \frac{1}{1+a\cdot(x^n_{Hey2}+x^n_{PRDM14}+x^n_{Sox2}+x^n_{c-Jun})} - x_{Gata4/6}$$

$$\frac{dx_{Hes1}}{dt} = \frac{a\cdot(x^n_{TGF-\beta}+x^n_{Notch})}{1+a\cdot(x^n_{TGF-\beta}+x^n_{Notch})} - x_{Hes1}$$

$$\frac{dx_{Hey2}}{dt} = \frac{a\cdot(x^n_{Notch})}{1+a\cdot(x^n_{Notch})} - x_{Hey2}$$

$$\frac{dx_{HNF1}}{dt} = \frac{a\cdot(x^n_{HNF4\alpha}+x^n_{Notch})}{1+a\cdot(x^n_{HNF4\alpha}+x^n_{Notch})} \times \frac{1}{1+a\cdot(x^n_{C/EBP\alpha})} - x_{HNF1}$$

$$\frac{dx_{HNF4\alpha}}{dt} = \frac{a\cdot(x^n_{C/EBP\alpha}+x^n_{Cdx2}+x^n_{Gata4/6}+x^n_{HNF1}+x^n_{NR2F2}+x^n_{Stat5}+x^n_{E-Cadherin})}{1+a\cdot(x^n_{C/EBP\alpha}+x^n_{Cdx2}+x^n_{Gata4/6}+x^n_{HNF1}+x^n_{NR2F2}+x^n_{Stat5}+x^n_{E-Cadherin})} \times \frac{1}{1+a\cdot(x^n_{Sox2}+x^n_{NR4A}+x^n_{NF-\kappa B}+x^n_{Stat3}+x^n_{IR}+x^n_{MAPK}+x^n_{PKA}+x^n_{p53})} - x_{HNF4\alpha}$$

$$\frac{dx_{Hoxa10}}{dt} = \frac{a\cdot(x^n_{Hoxa9}+x^n_{Stat5}+x^n_{BMP})}{1+a\cdot(x^n_{Hoxa9}+x^n_{Stat5}+x^n_{BMP})}\times\frac{1}{1+a\cdot(x^n_{Cdx2}+x^n_{IL-1})} - x_{Hoxa10}$$

$$\frac{dx_{Hoxa3}}{dt} = \frac{a\cdot(x^n_{RARs}+x^n_{c-Jun}+x^n_{Wnt})}{1+a\cdot(x^n_{RARs}+x^n_{c-Jun}+x^n_{Wnt})}\times\frac{1}{1+a\cdot(x^n_{Stat3})} - x_{Hoxa3}$$

$$\frac{dx_{Hoxa5}}{dt} = \frac{a\cdot(x^n_{Cdx2}+x^n_{Hoxa10}+x^n_{RARs})}{1+a\cdot(x^n_{Cdx2}+x^n_{Hoxa10}+x^n_{RARs})}\times\frac{1}{1+a\cdot(x^n_{Runx1})} - x_{Hoxa5}$$

$$\frac{dx_{Hoxa9}}{dt} = \frac{a\cdot(x^n_{C/EBP\alpha}+x^n_{Stat5}+x^n_{Notch})}{1+a\cdot(x^n_{C/EBP\alpha}+x^n_{Stat5}+x^n_{Notch})}\times\frac{1}{1+a\cdot(x^n_{NF-\kappa B}+x^n_{TGF-\beta})} - x_{Hoxa9}$$

$$\frac{dx_{PGC-1}}{dt} = \frac{a\cdot(x^n_{C/EBP\beta})}{1+a\cdot(x^n_{C/EBP\beta})}\times\frac{1}{1+a\cdot(x^n_{Akt}+x^n_{c-Jun}+x^n_{p53})} - x_{PGC-1}$$

$$\frac{dx_{PRDM14}}{dt} = \frac{a\cdot(x^n_{Stat3})}{1+a\cdot(x^n_{Stat3})} - x_{PRDM14}$$

$$\frac{dx_{Pu.1}}{dt} = \frac{a\cdot(x^n_{C/EBP\alpha}+x^n_{Runx1})}{1+a\cdot(x^n_{C/EBP\alpha}+x^n_{Runx1})}\times\frac{1}{1+a\cdot(x^n_{Gata1/2}+x^n_{Sox4})} - x_{Pu.1}$$

$$\frac{dx_{Runx1}}{dt} = \frac{a\cdot(x^n_{Cdx2}+x^n_{Gata1/2}+x^n_{Sox2}+x^n_{BMP}+x^n_{c-Jun}+x^n_{Notch})}{1+a\cdot(x^n_{Cdx2}+x^n_{Gata1/2}+x^n_{Sox2}+x^n_{BMP}+x^n_{c-Jun}+x^n_{Notch})}\times\frac{1}{1+a\cdot(x^n_{Hoxa3}+x^n_{Stat5})} - x_{Runx1}$$

$$\frac{dx_{Runx2}}{dt} = \frac{a\cdot(x^n_{Hoxa10}+x^n_{Runx1}+x^n_{BMP})}{1+a\cdot(x^n_{Hoxa10}+x^n_{Runx1}+x^n_{BMP})}\times\frac{1}{1+a\cdot(x^n_{C/EBP\beta}+x^n_{NR2F2}+x^n_{Stat5}+x^n_{TGF-\beta}+x^n_{FGFR3}+x^n_{Notch})} - x_{Runx2}$$

$$\frac{dx_{Sox2}}{dt} = \frac{a\cdot(x^n_{Stat3}+x^n_{FGFR2}+x^n_{AP2}+x^n_{Wnt})}{1+a\cdot(x^n_{Stat3}+x^n_{FGFR2}+x^n_{AP2}+x^n_{Wnt})}\times\frac{1}{1+a\cdot(x^n_{p21}+x^n_{Cdx2})} - x_{Sox2}$$

$$\frac{dx_{Sox4}}{dt} = \frac{a\cdot(x^n_{Sox7}+x^n_{Stat5}+x^n_{TGF-\beta}+x^n_{Wnt})}{1+a\cdot(x^n_{Sox7}+x^n_{Stat5}+x^n_{TGF-\beta}+x^n_{Wnt})}\times\frac{1}{1+a\cdot(x^n_{C/EBP\alpha})} - x_{Sox4}$$

$$\frac{dx_{Sox7}}{dt} = \frac{a\cdot(x^n_{BMP}+x^n_{VEGF}+x^n_{c-Jun})}{1+a\cdot(x^n_{BMP}+x^n_{VEGF}+x^n_{c-Jun})}\times\frac{1}{1+a\cdot(x^n_{PRDM14})} - x_{Sox7}$$

$$\frac{dx_{Sox9}}{dt} = \frac{a\cdot(x^n_{TGF-\beta}+x^n_{BMP}+x^n_{FGFR3}+x^n_{AP2}+x^n_{PKA}+x^n_{Notch}+x^n_{SHH}+x^n_{Wnt})}{1+a\cdot(x^n_{TGF-\beta}+x^n_{BMP}+x^n_{FGFR3}+x^n_{AP2}+x^n_{PKA}+x^n_{Notch}+x^n_{SHH}+x^n_{Wnt})}$$
$$\times\frac{1}{1+a\cdot(x^n_{RARs}+x^n_{IL-1}+x^n_{NF-\kappa B}+x^n_{c-Jun})} - x_{Sox9}$$

$$\frac{dx_{PPAR\gamma}}{dt} = \frac{a\cdot(x^n_{C/EBP\alpha}+x^n_{C/EBP\beta})}{1+a\cdot(x^n_{C/EBP\alpha}+x^n_{C/EBP\beta})}\times\frac{1}{1+a\cdot(x^n_{RARs}+x^n_{NR2F2}+x^n_{Stat5}+x^n_{TGF-\beta}+x^n_{TNF-\alpha}+x^n_{SHH})} - x_{PPAR\gamma}$$

$$\frac{dx_{GR}}{dt} = \frac{a\cdot(x^n_{HNF1}+x^n_{TGF-\beta})}{1+a\cdot(x^n_{HNF1}+x^n_{TGF-\beta})}\times\frac{1}{1+a\cdot(x^n_{NR4A}+x^n_{NR2F2})} - x_{GR}$$

$$\frac{dx_{NR4A}}{dt} = \frac{a\cdot(x^n_{Runx1}+x^n_{EGFR}+x^n_{VEGF})}{1+a\cdot(x^n_{Runx1}+x^n_{EGFR}+x^n_{VEGF})}\times\frac{1}{1+a\cdot(x^n_{HNF4\alpha}+x^n_{GR})} - x_{NR4A}$$

$$\frac{dx_{RARs}}{dt} = \frac{a\cdot(x^n_{PPAR\gamma}+x^n_{AP2})}{1+a\cdot(x^n_{PPAR\gamma}+x^n_{AP2})}\times\frac{1}{1+a\cdot(x^n_{NR2F2}+x^n_{MAPK})} - x_{RARs}$$

$$\frac{dx_{NR2F2}}{dt} = \frac{a\cdot(x^n_{Gata4/6}+x^n_{HNF4\alpha}+x^n_{GR}+x^n_{MAPK}+x^n_{SHH}+x^n_{Wnt})}{1+a\cdot(x^n_{Gata4/6}+x^n_{HNF4\alpha}+x^n_{GR}+x^n_{MAPK}+x^n_{SHH}+x^n_{Wnt})} - x_{NR2F2}$$

$$\frac{dx_{G-CSR}}{dt} = \frac{a\cdot(x^n_{C/EBP\alpha}+x^n_{Pu.1}+x^n_{NF-\kappa B})}{1+a\cdot(x^n_{C/EBP\alpha}+x^n_{Pu.1}+x^n_{NF-\kappa B})}\times\frac{1}{1+a\cdot(x^n_{GR})} - x_{G-CSR}$$

$$\frac{dx_{IL-1}}{dt} = \frac{a\cdot(x^n_{C/EBP\beta}+x^n_{NF-\kappa B}+x^n_{OPN})}{1+a\cdot(x^n_{C/EBP\beta}+x^n_{NF-\kappa B}+x^n_{OPN})}\times\frac{1}{1+a\cdot(x^n_{C/EBP\alpha}+x^n_{Akt})} - x_{IL-1}$$

$$\frac{dx_{IL-10}}{dt} = \frac{a\cdot(x^n_{Fas}+x^n_{TNF-\alpha})}{1+a\cdot(x^n_{Fas}+x^n_{TNF-\alpha})}\times\frac{1}{1+a\cdot(x^n_{IL-10})} - x_{IL-10}$$

$$\frac{dx_{IL-3}}{dt} = \frac{a\cdot(x^n_{C/EBP\alpha}+x^n_{Gata1/2}+x^n_{Runx1})}{1+a\cdot(x^n_{C/EBP\alpha}+x^n_{Gata1/2}+x^n_{Runx1})} - x_{IL-3}$$

$$\frac{dx_{IL-6}}{dt} = \frac{a\cdot(x^n_{C/EBP\beta}+x^n_{Hoxa3}+x^n_{NF-\kappa B}+x^n_{NGAL})}{1+a\cdot(x^n_{C/EBP\beta}+x^n_{Hoxa3}+x^n_{NF-\kappa B}+x^n_{NGAL})}\times\frac{1}{1+a\cdot(x^n_{HNF4\alpha}+x^n_{Sox2}+x^n_{RARs})} - x_{IL-6}$$

$$\frac{dx_{i\kappa B}}{dt} = \frac{a\cdot(x^n_{GR}+x^n_{NR4A}+x^n_{NF-\kappa B}+x^n_{TGF-\beta})}{1+a\cdot(x^n_{GR}+x^n_{NR4A}+x^n_{NF-\kappa B}+x^n_{TGF-\beta})}\times\frac{1}{1+a\cdot(x^n_{Fas}+x^n_{TNF-\alpha}+x^n_{EGFR}+x^n_{Akt})} - x_{i\kappa B}$$

$$\frac{dx_{NF-\kappa B}}{dt} = \frac{a\cdot(x^n_{C/EBP\beta}+x^n_{IL-1}+x^n_{TNF-\alpha}+x^n_{CCK})}{1+a\cdot(x^n_{C/EBP\beta}+x^n_{IL-1}+x^n_{TNF-\alpha}+x^n_{CCK})}\times\frac{1}{1+a\cdot(x^n_{Cdx2}+x^n_{Foxa1}+x^n_{Sox2}+x^n_{PPAR\gamma}+x^n_{IL-10}+x^n_{i\kappa B}+x^n_{IR})} - x_{NF-\kappa B}$$

$$\frac{dx_{Stat3}}{dt} = \frac{a\cdot(x^n_{G-CSR}+x^n_{IL-6}+x^n_{CCK}+x^n_{FGFR2}+x^n_{FGFR3}+x^n_{FGFR4}+x^n_{Gast}+x^n_{HGF}+x^n_{VEGF}+x^n_{OPN})}{1+a\cdot(x^n_{G-CSR}+x^n_{IL-6}+x^n_{CCK}+x^n_{FGFR2}+x^n_{FGFR3}+x^n_{FGFR4}+x^n_{Gast}+x^n_{HGF}+x^n_{VEGF}+x^n_{OPN})}$$
$$\times\frac{1}{1+a\cdot(x^n_{Gata1/2}+x^n_{PPAR\gamma}+x^n_{RARs}+x^n_{PTEN})} - x_{Stat3}$$

$$\frac{dx_{Stat5}}{dt} = \frac{a\cdot(x^n_{G-CSR}+x^n_{IL-3}+x^n_{FGFR2}+x^n_{IR}+x^n_{VEGF})}{1+a\cdot(x^n_{G-CSR}+x^n_{IL-3}+x^n_{FGFR2}+x^n_{IR}+x^n_{VEGF})}\times\frac{1}{1+a\cdot(x^n_{HNF4\alpha}+x^n_{Runx1}+x^n_{Runx2}+x^n_{TGF-\beta})} - x_{Stat5}$$

$$\frac{dx_{TGF-\beta}}{dt} = \frac{a\cdot(x^n_{Hoxa10}+x^n_{Hoxa3}+x^n_{Hoxa9}+x^n_{Sox7}+x^n_{Stat3}+x^n_{TNF-\alpha}+x^n_{p53}+x^n_{NOG}+x^n_{OPN})}{1+a\cdot(x^n_{Hoxa10}+x^n_{Hoxa3}+x^n_{Hoxa9}+x^n_{Sox7}+x^n_{Stat3}+x^n_{TNF-\alpha}+x^n_{p53}+x^n_{NOG}+x^n_{OPN})} \times\frac{1}{1+a\cdot(x^n_{Gata4/6}+x^n_{Sox2}+x^n_{RARs}+x^n_{NR2F2}+x^n_{NF-\kappa B}+x^n_{Stat5})} - x_{TGF-\beta}$$

$$\frac{dx_{TNF-\alpha}}{dt} = \frac{a\cdot(x^n_{IL-1}+x^n_{NF-\kappa B})}{1+a\cdot(x^n_{IL-1}+x^n_{NF-\kappa B})}\times\frac{1}{1+a\cdot(x^n_{IL-10})} - x_{TNF-\alpha}$$

$$\frac{dx_{BMP}}{dt} = \frac{a\cdot(x^n_{Gata4/6}+x^n_{Runx1}+x^n_{EGFR}+x^n_{FGFR2})}{1+a\cdot(x^n_{Gata4/6}+x^n_{Runx1}+x^n_{EGFR}+x^n_{FGFR2})}\times\frac{1}{1+a\cdot(x^n_{HNF4\alpha}+x^n_{NR2F2}+x^n_{TGF-\beta}+x^n_{NOG})} - x_{BMP}$$

$$\frac{dx_{CCK}}{dt} = \frac{a\cdot(x^n_{C/EBP\beta}+x^n_{Cdx2}+x^n_{Gata4/6}+x^n_{IL-1}+x^n_{Gast}+x^n_{IR})}{1+a\cdot(x^n_{C/EBP\beta}+x^n_{Cdx2}+x^n_{Gata4/6}+x^n_{IL-1}+x^n_{Gast}+x^n_{IR})} - x_{CCK}$$

$$\frac{dx_{EGFR}}{dt} = \frac{a\cdot(x^n_{Myc}+x^n_{Runx1}+x^n_{Sox4}+x^n_{HIF})}{1+a\cdot(x^n_{Myc}+x^n_{Runx1}+x^n_{Sox4}+x^n_{HIF})}\times\frac{1}{1+a\cdot(x^n_{PTEN})} - x_{EGFR}$$

$$\frac{dx_{FGF2}}{dt} = \frac{a\cdot(x^n_{Hoxa10}+x^n_{HSPG2})}{1+a\cdot(x^n_{Hoxa10}+x^n_{HSPG2})} - x_{FGF2}$$

$$\frac{dx_{FGF4/8}}{dt} = \frac{a\cdot(x^n_{SHH})}{1+a\cdot(x^n_{SHH})}\times\frac{1}{1+a\cdot(x^n_{Notch})} - x_{FGF4/8}$$

$$\frac{dx_{FGF7}}{dt} = \frac{a\cdot(x^n_{IL-1}+x^n_{HSPG2})}{1+a\cdot(x^n_{IL-1}+x^n_{HSPG2})} - x_{FGF7}$$

$$\frac{dx_{FGF9}}{dt} = \frac{a\cdot(x^n_{Sox9})}{1+a\cdot(x^n_{Sox9})} - x_{FGF9}$$

$$\frac{dx_{FGFR2}}{dt} = \frac{a\cdot(x^n_{Hoxa10}+x^n_{Runx2}+x^n_{FGF2}+x^n_{FGF7})}{1+a\cdot(x^n_{Hoxa10}+x^n_{Runx2}+x^n_{FGF2}+x^n_{FGF7})}\times\frac{1}{1+a\cdot(x^n_{PRDM14})} - x_{FGFR2}$$

$$\frac{dx_{FGFR3}}{dt} = \frac{a\cdot(x^n_{FGF9})}{1+a\cdot(x^n_{FGF9})} - x_{FGFR3}$$

$$\frac{dx_{FGFR4}}{dt} = \frac{a\cdot(x^n_{FGF4/8}+x^n_{FGF9})}{1+a\cdot(x^n_{FGF4/8}+x^n_{FGF9})} - x_{FGFR4}$$

$$\frac{dx_{Gast}}{dt} = \frac{a\cdot(x^n_{Sox2}+x^n_{TGF-\beta}+x^n_{Wnt})}{1+a\cdot(x^n_{Sox2}+x^n_{TGF-\beta}+x^n_{Wnt})} - x_{Gast}$$

$$\frac{dx_{HGF}}{dt} = \frac{a\cdot(x^n_{NF-\kappa B}+x^n_{c-Jun}+x^n_{HIF})}{1+a\cdot(x^n_{NF-\kappa B}+x^n_{c-Jun}+x^n_{HIF})}\times\frac{1}{1+a\cdot(x^n_{GR}+x^n_{TGF-\beta})} - x_{HGF}$$

$$\frac{dx_{IR}}{dt} = \frac{a\cdot(x^n_{C/EBP\alpha}+x^n_{PPAR\gamma}+x^n_{SHH}+x^n_{Wnt})}{1+a\cdot(x^n_{C/EBP\alpha}+x^n_{PPAR\gamma}+x^n_{SHH}+x^n_{Wnt})}\times\frac{1}{1+a\cdot(x^n_{TNF-\alpha}+x^n_{Notch})} - x_{IR}$$

$$\frac{dx_{VEGF}}{dt} = \frac{a\cdot(x^n_{Gata4/6}+x^n_{PGC-1}+x^n_{NR4A}+x^n_{NR2F2}+x^n_{Stat3}+x^n_{TGF-\beta}+x^n_{HIF})}{1+a\cdot(x^n_{Gata4/6}+x^n_{PGC-1}+x^n_{NR4A}+x^n_{NR2F2}+x^n_{Stat3}+x^n_{TGF-\beta}+x^n_{HIF})} \times\frac{1}{1+a\cdot(x^n_{Runx1}+x^n_{Sox9}+x^n_{RARs}+x^n_{Notch})} - x_{VEGF}$$

$$\frac{dx_{Akt}}{dt} = \frac{a\cdot(x^n_{Ras}+x^n_{RARs}+x^n_{NF-\kappa B}+x^n_{CCK}+x^n_{EGFR}+x^n_{FGFR2}+x^n_{HGF}+x^n_{IR}+x^n_{c-Jun}+x^n_{PKA}+x^n_{Notch})}{1+a\cdot(x^n_{Ras}+x^n_{RARs}+x^n_{NF-\kappa B}+x^n_{CCK}+x^n_{EGFR}+x^n_{FGFR2}+x^n_{HGF}+x^n_{IR}+x^n_{c-Jun}+x^n_{PKA}+x^n_{Notch})} \times\frac{1}{1+a\cdot(x^n_{PTEN})} - x_{Akt}$$

$$\frac{dx_{AP2}}{dt} = \frac{a\cdot(x^n_{C/EBP\beta}+x^n_{RARs}+x^n_{NR2F2})}{1+a\cdot(x^n_{C/EBP\beta}+x^n_{RARs}+x^n_{NR2F2})} - x_{AP2}$$

$$\frac{dx_{c-Jun}}{dt} = \frac{a\cdot(x^n_{Runx1}+x^n_{TNF-\alpha}+x^n_{CCK}+x^n_{EGFR}+x^n_{FGFR3}+x^n_{Akt})}{1+a\cdot(x^n_{Runx1}+x^n_{TNF-\alpha}+x^n_{CCK}+x^n_{EGFR}+x^n_{FGFR3}+x^n_{Akt})}\times\frac{1}{1+a\cdot(x^n_{C/EBP\alpha}+x^n_{GR}+x^n_{Mkp2})} - x_{c-Jun}$$

$$\frac{dx_{HIF}}{dt} = \frac{a\cdot(x^n_{NR4A}+x^n_{Stat3}+x^n_{Akt})}{1+a\cdot(x^n_{NR4A}+x^n_{Stat3}+x^n_{Akt})}\times\frac{1}{1+a\cdot(x^n_{p53})} - x_{HIF}$$

$$\frac{dx_{MAPK}}{dt} = \frac{a\cdot(x^n_{Ras}+x^n_{PPAR\gamma}+x^n_{NR2F2}+x^n_{NF-\kappa B}+x^n_{FGFR2}+x^n_{FGFR3}+x^n_{Integrin})}{1+a\cdot(x^n_{Ras}+x^n_{PPAR\gamma}+x^n_{NR2F2}+x^n_{NF-\kappa B}+x^n_{FGFR2}+x^n_{FGFR3}+x^n_{Integrin})}\times\frac{1}{1+a\cdot(x^n_{TGF-\beta}+x^n_{PTEN})} - x_{MAPK}$$

$$\frac{dx_{Mkp2}}{dt} = \frac{a\cdot(x^n_{Hoxa10})}{1+a\cdot(x^n_{Hoxa10})} - x_{Mkp2}$$

$$\frac{dx_{PKA}}{dt} = \frac{a\cdot(x^n_{TGF-\beta}+x^n_{EGFR}+x^n_{Akt})}{1+a\cdot(x^n_{TGF-\beta}+x^n_{EGFR}+x^n_{Akt})}\times\frac{1}{1+a\cdot(x^n_{p53})} - x_{PKA}$$

$$\frac{dx_{PTEN}}{dt} = \frac{a\cdot(x^n_{PPAR\gamma}+x^n_{AP2}+x^n_{p53})}{1+a\cdot(x^n_{PPAR\gamma}+x^n_{AP2}+x^n_{p53})}\times\frac{1}{1+a\cdot(x^n_{NF-\kappa B}+x^n_{Stat3})} - x_{PTEN}$$

$$\frac{dx_{p53}}{dt} = \frac{a\cdot(x^n_{Myc}+x^n_{PPAR\gamma}+x^n_{RARs}+x^n_{AP2}+x^n_{PTEN})}{1+a\cdot(x^n_{Myc}+x^n_{PPAR\gamma}+x^n_{RARs}+x^n_{AP2}+x^n_{PTEN})}\times\frac{1}{1+a\cdot(x^n_{Sox4}+x^n_{Akt}+x^n_{Notch})} - x_{p53}$$

$$\frac{dx_{E-Cadherin}}{dt} = \frac{a\cdot(x^n_{Cdx2}+x^n_{Foxa1}+x^n_{Foxa2}+x^n_{HNF4\alpha}+x^n_{AP2})}{1+a\cdot(x^n_{Cdx2}+x^n_{Foxa1}+x^n_{Foxa2}+x^n_{HNF4\alpha}+x^n_{AP2})}$$

$$\times \frac{1}{1 + a \cdot (x^n_{Stat3} + x^n_{TGF-\beta} + x^n_{TNF-\alpha} + x^n_{EGFR} + x^n_{Akt} + x^n_{HIF} + x^n_{SHH})} - x_{E-Cadherin}$$

$$\frac{dx_{HSPG2}}{dt} = \frac{a \cdot (x^n_{Runx2} + x^n_{TGF-\beta})}{1 + a \cdot (x^n_{Runx2} + x^n_{TGF-\beta})} - x_{HSPG2}$$

$$\frac{dx_{Integrin}}{dt} = \frac{a \cdot (x^n_{TNF-\alpha} + x^n_{EGFR} + x^n_{VEGF} + x^n_{MAPK})}{1 + a \cdot (x^n_{TNF-\alpha} + x^n_{EGFR} + x^n_{VEGF} + x^n_{MAPK})} \times \frac{1}{1 + a \cdot (x^n_{Myc} + x^n_{Ras} + x^n_{SHH})} - x_{Integrin}$$

$$\frac{dx_{Notch}}{dt} = \frac{a \cdot (x^n_{Cdx2} + x^n_{HNF1} + x^n_{NF-\kappa B} + x^n_{Stat3} + x^n_{FGFR2} + x^n_{MAPK})}{1 + a \cdot (x^n_{Cdx2} + x^n_{HNF1} + x^n_{NF-\kappa B} + x^n_{Stat3} + x^n_{FGFR2} + x^n_{MAPK})}$$

$$\times \frac{1}{1 + a \cdot (x^n_{C/EBP\alpha} + x^n_{Hoxa5} + x^n_{NR2F2} + x^n_{BMP} + x^n_{E-Cadherin})} - x_{Notch}$$

$$\frac{dx_{NGAL}}{dt} = \frac{a \cdot (x^n_{Runx1} + x^n_{TNF-\alpha})}{1 + a \cdot (x^n_{Runx1} + x^n_{TNF-\alpha})} - x_{NGAL}$$

$$\frac{dx_{NOG}}{dt} = \frac{a \cdot (x^n_{Sox9} + x^n_{SHH} + x^n_{Wnt})}{1 + a \cdot (x^n_{Sox9} + x^n_{SHH} + x^n_{Wnt})} \times \frac{1}{1 + a \cdot (x^n_{FGFR2})} - x_{NOG}$$

$$\frac{dx_{OPN}}{dt} = \frac{a \cdot (x^n_{Runx1} + x^n_{Runx2} + x^n_{TGF-\beta})}{1 + a \cdot (x^n_{Runx1} + x^n_{Runx2} + x^n_{TGF-\beta})} \times \frac{1}{1 + a \cdot (x^n_{PGC-1})} - x_{OPN}$$

$$\frac{dx_{SHH}}{dt} = \frac{a \cdot (x^n_{Foxa1} + x^n_{Sox2} + x^n_{TGF-\beta})}{1 + a \cdot (x^n_{Foxa1} + x^n_{Sox2} + x^n_{TGF-\beta})} \times \frac{1}{1 + a \cdot (x^n_{Foxa2} + x^n_{Hes1} + x^n_{BMP} + x^n_{FGFR2})} - x_{SHH}$$

$$\frac{dx_{Wnt}}{dt} = \frac{a \cdot (x^n_{Gata4/6} + x^n_{PGC-1} + x^n_{Sox4} + x^n_{TGF-\beta} + x^n_{EGFR} + x^n_{FGFR2} + x^n_{FGFR3} + x^n_{HGF} + x^n_{IR})}{1 + a \cdot (x^n_{Gata4/6} + x^n_{PGC-1} + x^n_{Sox4} + x^n_{TGF-\beta} + x^n_{EGFR} + x^n_{FGFR2} + x^n_{FGFR3} + x^n_{HGF} + x^n_{IR})}$$

$$\times \frac{1}{1 + a \cdot (x^n_{Cdx2} + x^n_{HNF4\alpha} + x^n_{Sox7} + x^n_{Sox9})} - x_{Wnt}$$

B. Robustness of the modeling results

1. *Random parameter tests*

The results and details of the random parameter tests are summarized in Fig. S2.

2. *Robustness tests by using alternative forms of equations*

We use two alternative forms of differential equations to verify the robustness [1] of the attractors we obtained.

a. Alternative form (a) The first alternative form can be expressed as, for each x_i:

$$\frac{dx_i}{dt} = -x_i + \frac{1}{|act|} \left(\sum_{u \in act} \frac{a \cdot x^n_u}{1 + a \cdot x^n_u} \right) \cdot \frac{1}{1 + a \cdot (\sum_{v \in inh} x^n_v)} \tag{5}$$

where $i = 1, \ldots, 89$, act represents the set of all nodes that activate x_i and inh the set of nodes that inhibit x_i, and $|act|$ here means the total number of nodes in act. For instance,

if x_2, x_3, x_4 activate x_1 and x_5, x_6 inhibit x_1, we have

$$\frac{dx_1}{dt} = -x_1 + \frac{1}{3}\left(\frac{a \cdot x_2^n}{1 + a \cdot x_2^n} + \frac{a \cdot x_3^n}{1 + a \cdot x_3^n} + \frac{a \cdot x_4^n}{1 + a \cdot x_4^n}\right) \cdot \frac{1}{1 + a \cdot (x_5^n + x_6^n)} \tag{6}$$

Using *Euler method*, all 10 attractors are obtained (See Table S4).

b. Alternative form (b) A formal representation for the second form would be, for each x_i:

$$\frac{dx_i}{dt} = -x_i + \frac{a \cdot (\sum_{u \in act} x_u)^n}{1 + a \cdot (\sum_{u \in act} x_u)^n} \cdot \frac{1}{1 + a \cdot (\sum_{v \in inh} x_v)^n} \tag{7}$$

where $i = 1, \ldots, 89$, act represents the set of all nodes that activate x_i and inh the set of nodes that inhibit x_i. For example, if x_2, x_3, x_4 activate x_1 and x_5, x_6 inhibit x_1, we have

$$\frac{dx_1}{dt} = -x_1 + \frac{a \cdot (x_2 + x_3 + x_4)^n}{1 + a \cdot (x_2 + x_3 + x_4)^n} \cdot \frac{1}{1 + a \cdot (x_5 + x_6)^n} \ . \tag{8}$$

By using *Euler method*, 9 attractors are found with values listed in Table S5. Among them, 8 attractors directly correspond to those obtained in in Table S3. Interestingly, we also found a limit cycle attractor. The averaged value along one period of the limit cycle is put in Table S5. It has features of both S5 and S6.

3. Comparison with Boolean dynamics

Boolean network modeling as an effective and simple tool have been used for the modeling of biological networks [2] and endogenous molecular-cellular networks [3–5]. To test the robustness of the attractors obtained from differential equations, we employed Boolean network with threshold functions to model our network as well. Each x_i has two states, ON $x_i = 1$ or OFF $x_i = 0$. The dynamics of the Boolean network is defined as

$$x_i(t+1) = \begin{cases} 0 & \sum_j r_{ij} x_j(t) <= 0 \\ 1 & \sum_j r_{ij} x_j(t) > 0 \end{cases}$$

where the adjacent matrix R for the network has elements r_{ij}. $r_{ij} = 1$ represents that node i is activated by node j, and $r_{ij} = -100$ denotes node i is inhibited by node j. Inhibition

is dominant in our model corresponds to a usual biological situation. We show that the attractors obtained from differential equations are consistent Boolean network modeling in Table S6.

C. Transitions between attractors

There are different ways of implementing transitions between attractors. They may be classified as: a) stochastic transition as a result of internal fluctuation. For two attractors connected by a saddle point, the most probable path of the stochastic transition usually go through the saddle point. The landscape (Fig. 3(a)) demonstrates the connections between attractors by saddle and other unstable fixed points as a roadmap of stochastic transitions. b) induced transition by external perturbation. Perturbations may cause by environmental change or drug treatment. These perturbations can be impulsive, continuous, or has a specific pattern. The effects are able to be carried out based on our modeling results. The whole phase space (each point in the space represents a distinct state of the system) is separated by attractive basins of the attractors, which means, for instance, starting inside the basin of S1, the system will go to S1 afterwards. As an illustration, for a given attractor, we enumerate the combinations of 3 nodes with down-regulation to 0 and up-regulation to 1 separately to generate perturbed starting positions that may fall into other attractive basins. Following the trajectories calculated by Euler method, we obtain the attractor the system will finally reach. Systematic investigation on induced switching between all 10 attractors is performed. Part of the results is drawn in Fig. 3(b). It suggests new biomarkers for CRC.

---

[1] M. W. Hirsch and S. Smale, *Differential equations, dynamical systems, and linear algebra* (Academic Press, San Diego, 1974).

[2] S. Bornholdt, J R Soc Interface **5**, S85 (2008).

[3] G.-W. Wang, X.-M. Zhu, J.-R. Gu, and P. Ao, Interface Focus **4**, 20130064 (2014).

[4] X. Zhu, R. Yuan, L. Hood, and P. Ao, Prog Biophys Mol Biol **117**, 30 (2015).

[5] S. Li, X. Zhu, B. Liu, G. Wang, and P. Ao, Oncotarget **6**, 13607 (2015).

III. MORE SUPPLEMENTARY FIGURES AND TABLES

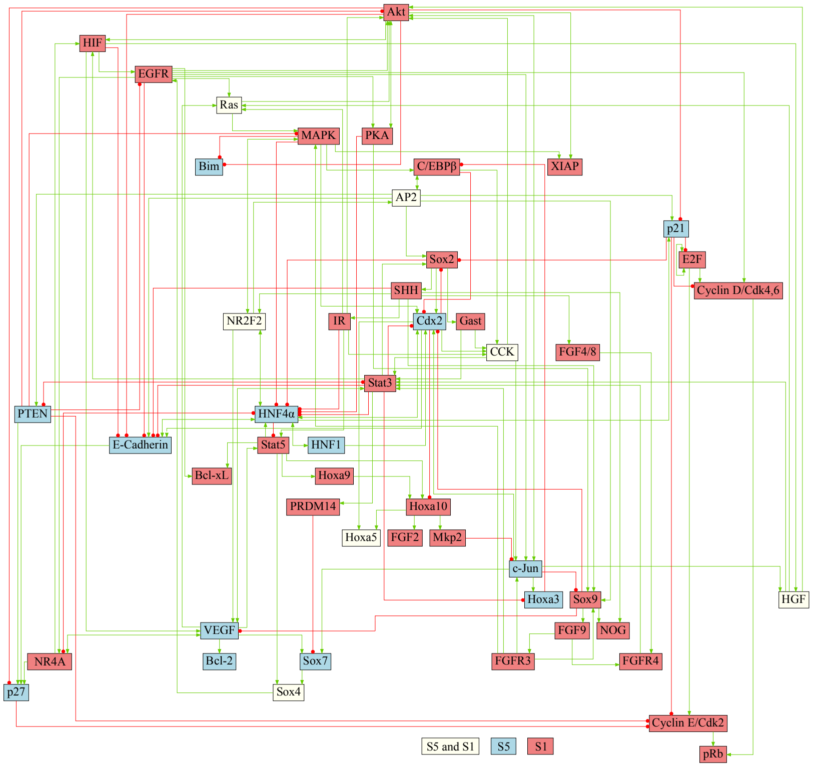

(a)Active nodes in S5 and S1

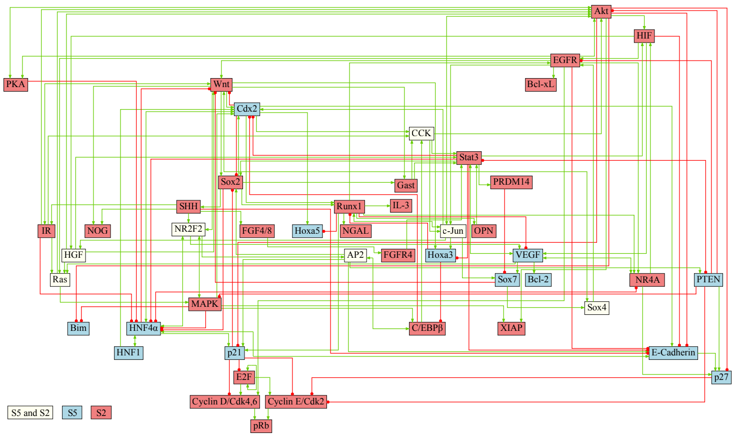

(b)Active nodes in S5 and S2

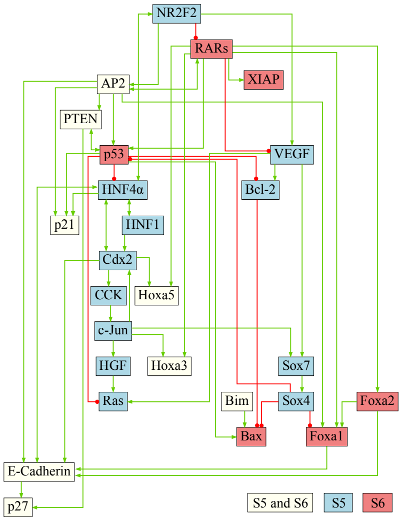

(c)Active nodes in S5 and S6

Fig. S1. Effective sub-networks between attractors. Effective sub-networks by selecting active nodes (> 0.5). Effective network for attractors (S5, S1), (S5, S2) and (S5, S6) (see Fig. 2) are overlaid in (a), (b), and (c). S1 and S2 are possible CRC like attractor. S5 is a normal intestine like attractor. S6 is mis-differentiated like attractor. The first graph shows that attractors S1 and S5 have few nodes in common. These two attractors form a kind of "switch" since the interactions between their specific active nodes are mostly inhibitory. They are mutually suppressing at network level. The third graph suggests that stress signaling through p53 is a potential source for mis-differentiation.

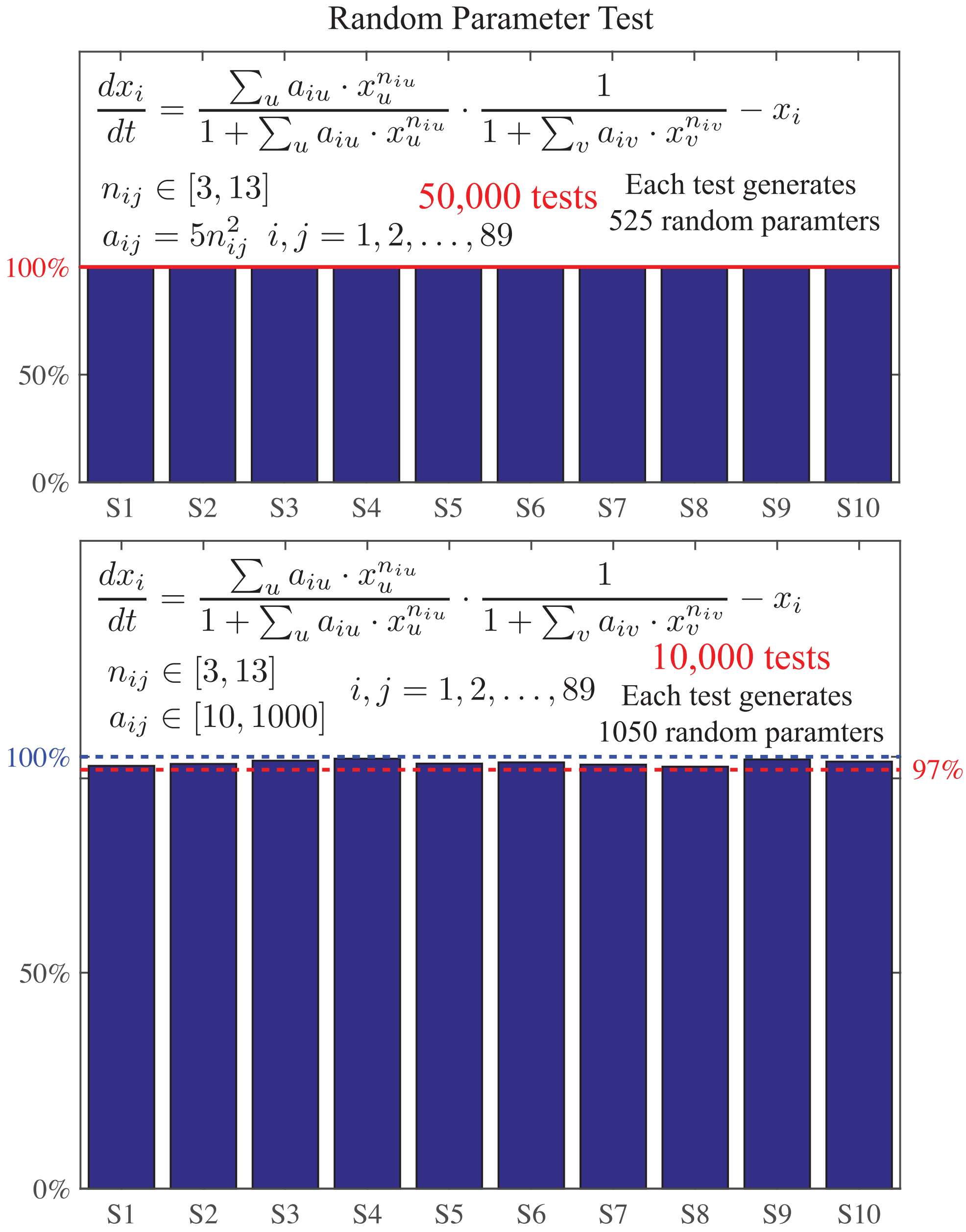

Fig. S2. Recurrence rate of attractors under random parameters. In the first test, we use the equations that enable different n_{ij} for each of the 525 interactions $i, j = 1, \ldots, 89$, where agent j to agent i has an interaction. With uniformly distributed real random numbers $n_{ij} \in [3, 13]$ and $a_{ij} = 5n_{ij}^2$, 50,000 tests show that all 10 attractors have 100% recurrence rate. In the second test, we also allow a_{ij} and n_{ij} are independent. Under the setting, we generate 10,000 sets (each set has $525 \times 2 = 1050$ random numbers) of random parameters with $a_{ij} \in [10, 1000]$, and $n_{ij} \in [3, 13]$, put into the equations, and calculate attractors using Euler method. The recurrence rate of each attractor calculated in Table S3 are shown here: the rates for all attractors are above 97%. Note that the $\sum_u$ means summation over all nodes activating node i and the $\sum_v$ means summation over all nodes inhibiting node i.

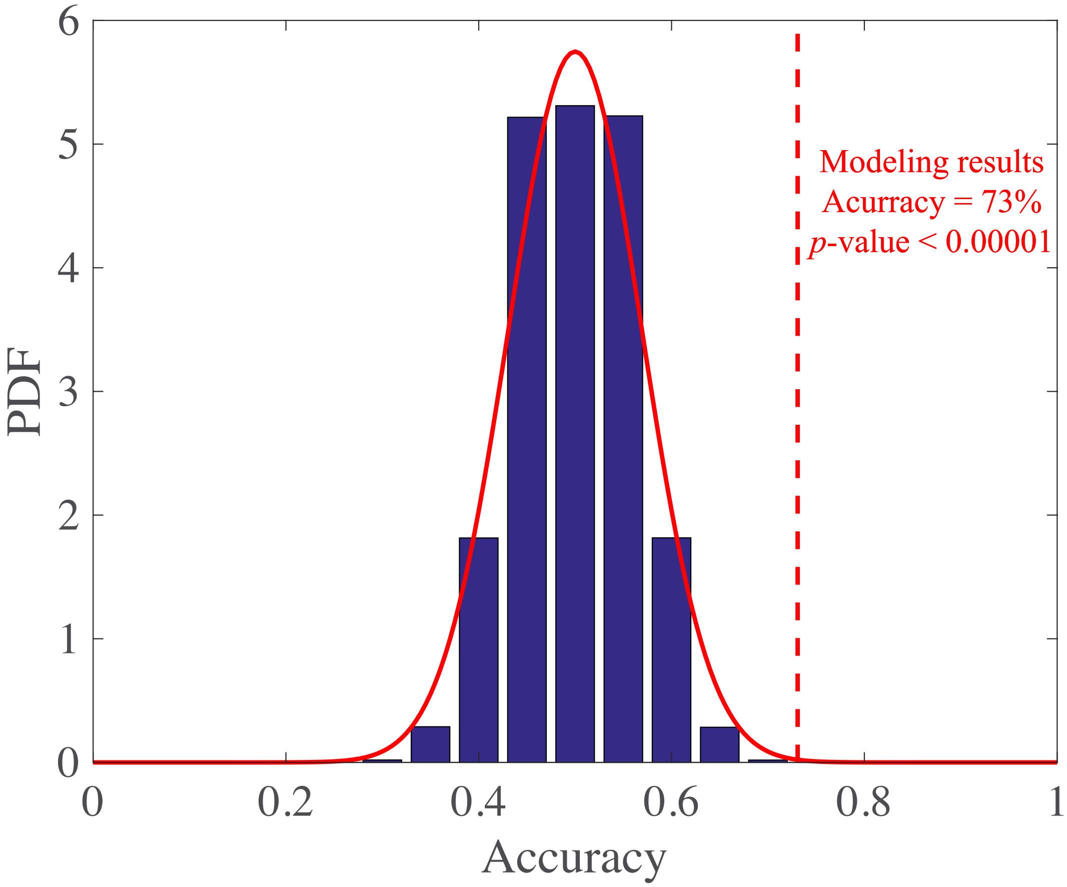

Fig. S3. Hypothesis test of modeling results *vs.* random guess. We generate one million random vectors consisting of 1 and -1. The distribution of the accuracy in comparing with the microarray data is shown here. We use a threshold value 5% for processing data (see Materials and Methods) with 78 genes determined as upregulated or downregulated in cancer. The modeling results have an accuracy rate 73% that corresponds to p-value < 0.00001.

Table S3. Molecular profile of computed attractors

	S1	S2	S3	S4	S5	S6	S7	S8	S9	S10
Cyclin D/Cdk4,6	0.93	0.93	0.88	0.89	0.00	0.00	0.93	0.93	0.89	0.88
Cyclin E/Cdk2	0.85	0.84	0.01	0.01	0.00	0.00	0.84	0.85	0.01	0.01
E2F	0.86	0.86	0.01	0.01	0.00	0.00	0.86	0.86	0.01	0.01
Myc	0.10	0.10	0.10	0.10	0.00	0.00	0.10	0.10	0.10	0.10
p21	0.09	0.09	0.09	0.09	0.90	0.93	0.09	0.09	0.09	0.09
p27	0.09	0.09	0.09	0.09	0.90	0.93	0.09	0.09	0.09	0.09
pRb	0.93	0.93	0.87	0.88	0.00	0.00	0.93	0.93	0.88	0.87
Ras	0.95	0.96	0.95	0.96	0.89	0.00	0.96	0.95	0.96	0.95
Bad	0.00	0.00	0.00	0.00	0.00	0.10	0.00	0.00	0.00	0.00
Bax	0.00	0.00	0.00	0.00	0.07	0.94	0.00	0.00	0.00	0.00
Bcl-2	0.01	0.02	0.01	0.02	0.82	0.00	0.00	0.00	0.00	0.00
Bcl-xL	0.93	0.90	0.93	0.90	0.05	0.00	0.13	0.13	0.13	0.13
Bim	0.05	0.05	0.05	0.05	0.95	0.99	0.05	0.05	0.05	0.05
Caspase 3	0.00	0.00	0.00	0.00	0.00	0.00	0.85	0.85	0.85	0.85
Cytochrome C	0.00	0.00	0.00	0.00	0.00	0.10	0.84	0.83	0.84	0.83
Fas	0.00	0.00	0.00	0.00	0.00	0.00	0.00	0.00	0.00	0.00
XIAP	0.95	0.95	0.95	0.95	0.09	0.86	0.07	0.07	0.07	0.07
C/EBPα	0.02	0.03	0.02	0.03	0.11	0.02	0.03	0.02	0.03	0.02
C/EBPβ	0.94	0.93	0.94	0.93	0.13	0.12	0.93	0.94	0.93	0.94
Cdx2	0.04	0.05	0.04	0.05	0.88	0.02	0.05	0.04	0.05	0.04
Foxa1	0.12	0.11	0.12	0.11	0.11	0.95	0.11	0.12	0.11	0.12
Foxa2	0.05	0.05	0.05	0.05	0.03	0.86	0.05	0.05	0.05	0.05
Gata1/2	0.10	0.09	0.10	0.09	0.07	0.00	0.09	0.10	0.09	0.10
Gata4/6	0.05	0.04	0.05	0.04	0.13	0.00	0.04	0.05	0.04	0.05
Hes1	0.00	0.01	0.00	0.01	0.02	0.02	0.01	0.00	0.01	0.00
Hey2	0.00	0.01	0.00	0.01	0.00	0.00	0.01	0.00	0.01	0.00
HNF1	0.00	0.01	0.00	0.01	0.86	0.01	0.01	0.00	0.01	0.00
HNF4α	0.02	0.02	0.02	0.02	0.87	0.09	0.02	0.02	0.02	0.02
Hoxa10	0.92	0.01	0.92	0.01	0.00	0.00	0.01	0.92	0.01	0.92
Hoxa3	0.00	0.10	0.00	0.10	0.84	0.87	0.10	0.00	0.10	0.00
Hoxa5	0.87	0.00	0.87	0.00	0.85	0.87	0.00	0.87	0.00	0.87
Hoxa9	0.86	0.02	0.86	0.02	0.03	0.00	0.02	0.86	0.02	0.86
PGC-1	0.09	0.05	0.09	0.05	0.00	0.00	0.05	0.09	0.05	0.09
PRDM14	0.90	0.90	0.90	0.90	0.03	0.00	0.90	0.90	0.90	0.90
Pu.1	0.00	0.11	0.00	0.11	0.00	0.00	0.11	0.00	0.11	0.00
Runx1	0.12	0.93	0.12	0.93	0.14	0.00	0.93	0.12	0.93	0.12
Runx2	0.03	0.05	0.03	0.05	0.00	0.00	0.05	0.03	0.05	0.03
Sox2	0.94	0.95	0.94	0.95	0.06	0.10	0.95	0.94	0.95	0.94
Sox4	0.86	0.90	0.86	0.90	0.87	0.02	0.90	0.86	0.90	0.86
Sox7	0.00	0.11	0.00	0.11	0.92	0.00	0.11	0.00	0.11	0.00
Sox9	0.94	0.10	0.94	0.10	0.12	0.12	0.10	0.94	0.10	0.94
PPARγ	0.04	0.05	0.04	0.05	0.00	0.00	0.05	0.04	0.05	0.04
GR	0.00	0.00	0.00	0.00	0.11	0.02	0.00	0.00	0.00	0.00
NR4A	0.89	0.94	0.89	0.94	0.11	0.00	0.94	0.89	0.94	0.89
RARs	0.05	0.05	0.05	0.05	0.11	0.87	0.05	0.05	0.05	0.05

	S1	S2	S3	S4	S5	S6	S7	S8	S9	S10
NR2F2	0.94	0.96	0.94	0.96	0.87	0.03	0.96	0.94	0.96	0.94
G-CSFR	0.00	0.01	0.00	0.01	0.03	0.00	0.01	0.00	0.01	0.00
IL-1	0.09	0.09	0.09	0.09	0.03	0.01	0.09	0.09	0.09	0.09
IL-10	0.00	0.00	0.00	0.00	0.00	0.00	0.00	0.00	0.00	0.00
IL-3	0.03	0.89	0.03	0.89	0.04	0.00	0.89	0.03	0.89	0.03
IL-6	0.10	0.10	0.10	0.10	0.11	0.12	0.10	0.10	0.10	0.10
iκB	0.05	0.05	0.05	0.05	0.05	0.02	0.05	0.05	0.05	0.05
NF-κB	0.06	0.05	0.06	0.05	0.11	0.00	0.05	0.06	0.05	0.06
Stat3	0.95	0.96	0.95	0.96	0.14	0.00	0.96	0.95	0.96	0.95
Stat5	0.85	0.10	0.85	0.10	0.11	0.00	0.10	0.85	0.10	0.85
TGF-β	0.04	0.05	0.04	0.05	0.12	0.12	0.05	0.04	0.05	0.04
TNF-α	0.01	0.01	0.01	0.01	0.01	0.00	0.01	0.01	0.01	0.01
BMP	0.05	0.05	0.05	0.05	0.00	0.00	0.05	0.05	0.05	0.05
CCK	0.96	0.96	0.96	0.96	0.87	0.02	0.96	0.96	0.96	0.96
EGFR	0.93	0.95	0.93	0.95	0.13	0.00	0.95	0.93	0.95	0.93
FGF2	0.89	0.00	0.89	0.00	0.00	0.00	0.00	0.89	0.00	0.89
FGF4/8	0.87	0.87	0.87	0.87	0.00	0.02	0.87	0.87	0.87	0.87
FGF7	0.01	0.01	0.01	0.01	0.00	0.00	0.01	0.01	0.01	0.01
FGF9	0.89	0.01	0.89	0.01	0.02	0.02	0.01	0.89	0.01	0.89
FGFR2	0.11	0.00	0.11	0.00	0.00	0.00	0.00	0.11	0.00	0.11
FGFR3	0.88	0.00	0.88	0.00	0.00	0.00	0.00	0.88	0.00	0.88
FGFR4	0.93	0.87	0.93	0.87	0.00	0.00	0.87	0.93	0.87	0.93
Gast	0.89	0.95	0.89	0.95	0.02	0.03	0.95	0.89	0.95	0.89
HGF	0.90	0.95	0.90	0.95	0.83	0.00	0.95	0.90	0.95	0.90
IR	0.87	0.93	0.87	0.93	0.02	0.02	0.93	0.87	0.93	0.87
VEGF	0.10	0.11	0.10	0.11	0.82	0.00	0.11	0.10	0.11	0.10
Akt	0.97	0.98	0.97	0.98	0.14	0.09	0.98	0.97	0.98	0.97
AP2	0.94	0.94	0.94	0.94	0.87	0.87	0.94	0.94	0.94	0.94
c-Jun	0.12	0.97	0.12	0.97	0.85	0.01	0.97	0.12	0.97	0.12
HIF	0.96	0.96	0.96	0.96	0.06	0.00	0.96	0.96	0.96	0.96
MAPK	0.95	0.94	0.95	0.94	0.14	0.00	0.94	0.95	0.94	0.95
Mkp2	0.89	0.00	0.89	0.00	0.00	0.00	0.00	0.89	0.00	0.89
PKA	0.94	0.95	0.94	0.95	0.06	0.00	0.95	0.94	0.95	0.94
PTEN	0.09	0.09	0.09	0.09	0.83	0.94	0.09	0.09	0.09	0.09
p53	0.05	0.05	0.05	0.05	0.12	0.95	0.05	0.05	0.05	0.05
E-Cadherin	0.02	0.02	0.02	0.02	0.87	0.91	0.02	0.02	0.02	0.02
HSPG2	0.00	0.00	0.00	0.00	0.02	0.02	0.00	0.00	0.00	0.00
Integrin	0.06	0.06	0.06	0.06	0.11	0.00	0.06	0.06	0.06	0.06
Notch	0.06	0.10	0.06	0.10	0.05	0.00	0.10	0.06	0.10	0.06
NGAL	0.02	0.89	0.02	0.89	0.02	0.00	0.89	0.02	0.89	0.02
NOG	0.92	0.94	0.92	0.94	0.02	0.03	0.94	0.92	0.94	0.92
OPN	0.02	0.89	0.02	0.89	0.04	0.02	0.89	0.02	0.89	0.02
SHH	0.88	0.89	0.88	0.89	0.03	0.12	0.89	0.88	0.89	0.88
Wnt	0.10	0.95	0.10	0.95	0.04	0.02	0.95	0.10	0.95	0.10

This table has the original data for Fig. 2. They are calculated using equations listed in Sect. II A 1 with parameter $n = 3$ and $a = 10$ by two methods: a) *Newtonian iteration method* for solving fixed point including attractors as well as saddle points and other unstable fixed points and b) *Euler method* for calculating trajectories of differential equations. They generate identical result. 9 of the 10 attractors are obtained while calculating with 10,000 random initial positions. The 10th attractor can be calculated using perturbation around unstable fixed points. We further run the program with 100,000 and 1,000,000 initial positions, acquiring identical 10 attractors.

Table S4. Recurrence of attractors using different forms of equations. Alternative equation (a). All attractors are obtained. Here E1 denotes the results from Table S3; E2 denotes the results from the alternative equation (a).

	S1		S2		S3		S4		S5		S6		S7		S8		S9		S10	
	E2	E1	E2	E1	E2	E1	E2	E1	E2	E1	E2	E1	E2	E1	E2	E1	E2	E1	E2	E1
Cyclin D/Cdk4,6	0.49	0.93	0.50	0.93	0.24	0.88	0.25	0.89	0.00	0.00	0.00	0.00	0.50	0.93	0.49	0.93	0.25	0.89	0.24	0.88
Cyclin E/Cdk2	0.49	0.85	0.49	0.84	0.00	0.01	0.00	0.01	0.00	0.00	0.00	0.00	0.49	0.84	0.49	0.85	0.00	0.01	0.00	0.01
E2F	0.49	0.86	0.49	0.86	0.00	0.01	0.00	0.01	0.00	0.00	0.00	0.00	0.49	0.86	0.49	0.86	0.00	0.01	0.00	0.01
Myc	0.00	0.10	0.00	0.10	0.00	0.10	0.00	0.10	0.00	0.00	0.00	0.00	0.00	0.10	0.00	0.10	0.00	0.10	0.00	0.10
p21	0.00	0.09	0.00	0.09	0.00	0.09	0.00	0.09	0.25	0.90	0.24	0.93	0.00	0.09	0.00	0.09	0.00	0.09	0.00	0.09
p27	0.00	0.09	0.00	0.09	0.00	0.09	0.00	0.09	0.40	0.90	0.40	0.93	0.00	0.09	0.00	0.09	0.00	0.09	0.00	0.09
pRb	0.98	0.93	0.98	0.93	0.44	0.87	0.44	0.88	0.00	0.00	0.00	0.00	0.98	0.93	0.98	0.93	0.44	0.88	0.44	0.87
Ras	0.47	0.95	0.50	0.96	0.47	0.95	0.50	0.96	0.20	0.89	0.00	0.00	0.50	0.96	0.47	0.95	0.50	0.96	0.47	0.95
Bad	0.00	0.00	0.00	0.00	0.00	0.00	0.00	0.00	0.00	0.00	0.03	0.10	0.00	0.00	0.00	0.00	0.00	0.00	0.00	0.00
Bax	0.00	0.00	0.00	0.00	0.00	0.00	0.00	0.00	0.02	0.07	0.99	0.94	0.00	0.00	0.00	0.00	0.00	0.00	0.00	0.00
Bcl-2	0.00	0.01	0.00	0.02	0.00	0.01	0.00	0.02	0.20	0.82	0.00	0.00	0.00	0.00	0.00	0.00	0.00	0.00	0.00	0.00
Bcl-xL	0.56	0.93	0.33	0.90	0.56	0.93	0.33	0.90	0.00	0.05	0.00	0.00	0.03	0.13	0.05	0.13	0.03	0.13	0.05	0.13
Bim	0.00	0.05	0.00	0.05	0.00	0.05	0.00	0.05	1.00	0.95	1.00	0.99	0.00	0.05	0.00	0.05	0.00	0.05	0.00	0.05
Caspase 3	0.00	0.00	0.00	0.00	0.00	0.00	0.00	0.00	0.00	0.00	0.00	0.00	0.23	0.85	0.22	0.85	0.23	0.85	0.22	0.85
Cytochrome C	0.00	0.00	0.00	0.00	0.00	0.00	0.00	0.00	0.00	0.00	0.02	0.10	0.28	0.84	0.26	0.83	0.28	0.84	0.26	0.83
Fas	0.00	0.00	0.00	0.00	0.00	0.00	0.00	0.00	0.00	0.00	0.00	0.00	0.00	0.00	0.00	0.00	0.00	0.00	0.00	0.00
XIAP	0.39	0.95	0.38	0.95	0.39	0.95	0.38	0.95	0.01	0.09	0.19	0.86	0.01	0.07	0.01	0.07	0.01	0.07	0.01	0.07
C/EBPα	0.00	0.02	0.00	0.03	0.00	0.02	0.00	0.03	0.02	0.11	0.00	0.02	0.00	0.03	0.00	0.02	0.00	0.03	0.00	0.02
C/EBPβ	0.66	0.94	0.64	0.93	0.66	0.94	0.64	0.93	0.04	0.13	0.01	0.12	0.64	0.93	0.66	0.94	0.64	0.93	0.66	0.94
Cdx2	0.00	0.04	0.00	0.05	0.00	0.04	0.00	0.05	0.29	0.88	0.00	0.02	0.00	0.05	0.00	0.04	0.00	0.05	0.00	0.04
Foxa1	0.05	0.12	0.02	0.11	0.05	0.12	0.02	0.11	0.02	0.11	0.96	0.95	0.02	0.11	0.05	0.12	0.02	0.11	0.05	0.12
Foxa2	0.00	0.05	0.00	0.05	0.00	0.05	0.00	0.05	0.01	0.03	0.33	0.86	0.00	0.05	0.00	0.05	0.00	0.05	0.00	0.05
Gata1/2	0.00	0.10	0.00	0.09	0.00	0.10	0.00	0.09	0.05	0.07	0.00	0.00	0.00	0.09	0.00	0.10	0.00	0.09	0.00	0.10
Gata4/6	0.00	0.05	0.00	0.04	0.00	0.05	0.00	0.04	0.10	0.13	0.00	0.00	0.00	0.04	0.00	0.05	0.00	0.04	0.00	0.05
Hes1	0.00	0.00	0.00	0.01	0.00	0.00	0.00	0.01	0.00	0.02	0.00	0.02	0.00	0.01	0.00	0.00	0.00	0.01	0.00	0.00
Hey2	0.00	0.00	0.00	0.01	0.00	0.00	0.00	0.01	0.00	0.00	0.00	0.00	0.00	0.01	0.00	0.00	0.00	0.01	0.00	0.00
HNF1	0.00	0.00	0.00	0.01	0.00	0.00	0.00	0.01	0.49	0.86	0.00	0.01	0.00	0.01	0.00	0.00	0.00	0.01	0.00	0.00
HNF4α	0.00	0.02	0.00	0.02	0.00	0.02	0.00	0.02	0.57	0.87	0.00	0.09	0.00	0.02	0.00	0.02	0.00	0.02	0.00	0.02
Hoxa10	0.53	0.92	0.00	0.01	0.53	0.92	0.00	0.01	0.00	0.00	0.00	0.00	0.00	0.01	0.53	0.92	0.00	0.01	0.53	0.92
Hoxa3	0.00	0.00	0.01	0.10	0.00	0.00	0.01	0.10	0.19	0.84	0.33	0.87	0.01	0.10	0.00	0.00	0.01	0.10	0.00	0.00
Hoxa5	0.32	0.87	0.00	0.00	0.32	0.87	0.00	0.00	0.30	0.85	0.33	0.87	0.00	0.00	0.32	0.87	0.00	0.00	0.32	0.87
Hoxa9	0.24	0.86	0.00	0.02	0.24	0.86	0.00	0.02	0.00	0.03	0.00	0.00	0.00	0.02	0.24	0.86	0.00	0.02	0.24	0.86
PGC-1	0.01	0.09	0.00	0.05	0.01	0.09	0.00	0.05	0.01	0.00	0.00	0.00	0.00	0.05	0.01	0.09	0.00	0.05	0.01	0.09
PRDM14	0.98	0.90	0.98	0.90	0.98	0.90	0.98	0.90	0.00	0.03	0.00	0.00	0.98	0.90	0.98	0.90	0.98	0.90	0.98	0.90
Pu.1	0.00	0.00	0.03	0.11	0.00	0.00	0.03	0.11	0.00	0.00	0.00	0.00	0.03	0.11	0.00	0.00	0.03	0.11	0.00	0.00
Runx1	0.03	0.12	0.33	0.93	0.03	0.12	0.33	0.93	0.03	0.14	0.00	0.00	0.33	0.93	0.03	0.12	0.33	0.93	0.03	0.12
Runx2	0.00	0.03	0.00	0.05	0.00	0.03	0.00	0.05	0.00	0.00	0.00	0.00	0.00	0.05	0.00	0.03	0.00	0.05	0.00	0.03
Sox2	0.49	0.94	0.74	0.95	0.49	0.94	0.74	0.95	0.01	0.06	0.02	0.10	0.74	0.95	0.49	0.94	0.74	0.95	0.49	0.94
Sox4	0.18	0.86	0.24	0.90	0.18	0.86	0.24	0.90	0.24	0.87	0.00	0.02	0.24	0.90	0.18	0.86	0.24	0.90	0.18	0.86
Sox7	0.00	0.00	0.00	0.11	0.00	0.00	0.00	0.11	0.38	0.92	0.00	0.00	0.00	0.11	0.00	0.00	0.00	0.11	0.00	0.00
Sox9	0.49	0.94	0.00	0.10	0.49	0.94	0.00	0.10	0.04	0.12	0.00	0.12	0.00	0.10	0.49	0.94	0.00	0.10	0.49	0.94
PPARγ	0.01	0.04	0.00	0.05	0.01	0.04	0.00	0.05	0.00	0.00	0.00	0.00	0.00	0.05	0.01	0.04	0.00	0.05	0.01	0.04
GR	0.00	0.00	0.00	0.00	0.00	0.00	0.00	0.00	0.04	0.11	0.00	0.02	0.00	0.00	0.00	0.00	0.00	0.00	0.00	0.00
NR4A	0.33	0.89	0.65	0.94	0.33	0.89	0.65	0.94	0.00	0.11	0.00	0.00	0.65	0.94	0.33	0.89	0.65	0.94	0.33	0.89
RARs	0.00	0.05	0.00	0.05	0.00	0.05	0.00	0.05	0.04	0.11	0.47	0.87	0.00	0.05	0.00	0.05	0.00	0.05	0.00	0.05
NR2F2	0.32	0.94	0.47	0.96	0.32	0.94	0.47	0.96	0.22	0.87	0.00	0.03	0.47	0.96	0.32	0.94	0.47	0.96	0.32	0.94
G-CSFR	0.00	0.00	0.00	0.01	0.00	0.00	0.00	0.01	0.00	0.03	0.00	0.00	0.00	0.01	0.00	0.00	0.00	0.01	0.00	0.00
IL-1	0.00	0.09	0.00	0.09	0.00	0.09	0.00	0.09	0.01	0.03	0.00	0.01	0.00	0.09	0.00	0.09	0.00	0.09	0.00	0.09
IL-10	0.00	0.00	0.00	0.00	0.00	0.00	0.00	0.00	0.00	0.00	0.00	0.00	0.00	0.00	0.00	0.00	0.00	0.00	0.00	0.00
IL-3	0.00	0.03	0.32	0.89	0.00	0.03	0.32	0.89	0.02	0.04	0.00	0.00	0.32	0.89	0.00	0.03	0.32	0.89	0.00	0.03
IL-6	0.00	0.10	0.00	0.10	0.00	0.10	0.00	0.10	0.00	0.11	0.00	0.12	0.00	0.10	0.00	0.10	0.00	0.10	0.00	0.10
iκB	0.00	0.05	0.00	0.05	0.00	0.05	0.00	0.05	0.01	0.05	0.00	0.02	0.00	0.05	0.00	0.05	0.00	0.05	0.00	0.05
NF-κB	0.00	0.06	0.00	0.05	0.00	0.06	0.00	0.05	0.01	0.11	0.00	0.00	0.00	0.05	0.00	0.06	0.00	0.05	0.00	0.06
Stat3	0.49	0.95	0.49	0.96	0.49	0.95	0.49	0.96	0.01	0.14	0.00	0.00	0.49	0.96	0.49	0.95	0.49	0.96	0.49	0.95
Stat5	0.17	0.85	0.01	0.10	0.17	0.85	0.01	0.10	0.00	0.11	0.00	0.00	0.01	0.10	0.17	0.85	0.01	0.10	0.17	0.85
TGF-β	0.00	0.04	0.00	0.05	0.00	0.04	0.00	0.05	0.01	0.12	0.00	0.12	0.00	0.05	0.00	0.04	0.00	0.05	0.00	0.04
TNF-α	0.00	0.01	0.00	0.01	0.00	0.01	0.00	0.01	0.00	0.01	0.00	0.00	0.00	0.01	0.00	0.01	0.00	0.01	0.00	0.01
BMP	0.00	0.05	0.00	0.05	0.00	0.05	0.00	0.05	0.00	0.00	0.00	0.00	0.00	0.05	0.00	0.05	0.00	0.05	0.00	0.05
CCK	0.47	0.96	0.49	0.96	0.47	0.96	0.49	0.96	0.21	0.87	0.00	0.02	0.49	0.96	0.47	0.96	0.49	0.96	0.47	0.96
EGFR	0.44	0.93	0.71	0.95	0.44	0.93	0.71	0.95	0.01	0.13	0.00	0.00	0.71	0.95	0.44	0.93	0.71	0.95	0.44	0.93
FGF2	0.49	0.89	0.00	0.00	0.49	0.89	0.00	0.00	0.00	0.00	0.00	0.00	0.00	0.00	0.49	0.89	0.00	0.00	0.49	0.89
FGF4/8	0.95	0.87	0.95	0.87	0.95	0.87	0.95	0.87	0.00	0.00	0.00	0.02	0.95	0.87	0.95	0.87	0.95	0.87	0.95	0.87
FGF7	0.00	0.01	0.00	0.01	0.00	0.01	0.00	0.01	0.00	0.00	0.00	0.00	0.00	0.01	0.00	0.01	0.00	0.01	0.00	0.01
FGF9	0.98	0.89	0.00	0.01	0.98	0.89	0.00	0.01	0.02	0.02	0.00	0.02	0.00	0.01	0.98	0.89	0.00	0.01	0.98	0.89
FGFR2	0.00	0.11	0.00	0.00	0.00	0.11	0.00	0.00	0.00	0.00	0.00	0.00	0.00	0.00	0.00	0.11	0.00	0.00	0.00	0.11
FGFR3	1.00	0.88	0.00	0.00	1.00	0.88	0.00	0.00	0.00	0.00	0.00	0.00	0.00	0.00	1.00	0.88	0.00	0.00	1.00	0.88
FGFR4	1.00	0.93	0.50	0.87	1.00	0.93	0.50	0.87	0.00	0.00	0.00	0.00	0.50	0.87	1.00	0.93	0.50	0.87	1.00	0.93
Gast	0.33	0.89	0.66	0.95	0.33	0.89	0.66	0.95	0.00	0.02	0.00	0.03	0.66	0.95	0.33	0.89	0.66	0.95	0.33	0.89
HGF	0.33	0.90	0.66	0.95	0.33	0.90	0.66	0.95	0.17	0.83	0.00	0.00	0.66	0.95	0.33	0.90	0.66	0.95	0.33	0.90
IR	0.24	0.87	0.48	0.93	0.24	0.87	0.48	0.93	0.00	0.02	0.00	0.02	0.48	0.93	0.24	0.87	0.48	0.93	0.24	0.87
VEGF	0.00	0.10	0.02	0.11	0.00	0.10	0.02	0.11	0.15	0.82	0.00	0.00	0.02	0.11	0.00	0.10	0.02	0.11	0.00	0.10
Akt	0.52	0.97	0.63	0.98	0.52	0.97	0.63	0.98	0.01	0.14	0.00	0.09	0.63	0.98	0.52	0.97	0.63	0.98	0.52	0.97
AP2	0.65	0.94	0.66	0.94	0.65	0.94	0.66	0.94	0.30	0.87	0.33	0.87	0.66	0.94	0.65	0.94	0.66	0.94	0.65	0.94
c-Jun	0.00	0.12	0.65	0.97	0.00	0.12	0.65	0.97	0.13	0.85	0.00	0.01	0.65	0.97	0.00	0.12	0.65	0.97	0.00	0.12
HIF	0.97	0.96	0.99	0.96	0.97	0.96	0.99	0.96	0.00	0.06	0.00	0.00	0.99	0.96	0.97	0.96	0.99	0.96	0.97	0.96
MAPK	0.42	0.95	0.28	0.94	0.42	0.95	0.28	0.94	0.01	0.14	0.00	0.00	0.28	0.94	0.42	0.95	0.28	0.94	0.42	0.95
Mkp2	0.99	0.89	0.00	0.00	0.99	0.89	0.00	0.00	0.00	0.00	0.00	0.00	0.00	0.00	0.99	0.89	0.00	0.00	0.99	0.89
PKA	0.65	0.94	0.66	0.95	0.65	0.94	0.66	0.95	0.00	0.06	0.00	0.00	0.66	0.95	0.65	0.94	0.66	0.95	0.65	0.94
PTEN	0.00	0.09	0.00	0.09	0.00	0.09	0.00	0.09	0.31	0.83	0.65	0.94	0.00	0.09	0.00	0.09	0.00	0.09	0.00	0.09
p53	0.00	0.05	0.00	0.05	0.00	0.05	0.00	0.05	0.03	0.12	0.58	0.95	0.00	0.05	0.00	0.05	0.00	0.05	0.00	0.05
E-Cadherin	0.00	0.02	0.00	0.02	0.00	0.02	0.00	0.02	0.57	0.87	0.58	0.91	0.00	0.02	0.00	0.02	0.00	0.02	0.00	0.02
HSPG2	0.00	0.00	0.00	0.00	0.00	0.00	0.00	0.00	0.00	0.02	0.00	0.02	0.00	0.00	0.00	0.00	0.00	0.00	0.00	0.00
Integrin	0.00	0.06	0.00	0.06	0.00	0.06	0.00	0.06	0.02	0.11	0.00	0.00	0.00	0.06	0.00	0.06	0.00	0.06	0.00	0.06
Notch	0.00	0.06	0.00	0.10	0.00	0.06	0.00	0.10	0.00	0.05	0.00	0.00	0.00	0.10	0.00	0.06	0.00	0.10	0.00	0.06
NGAL	0.01	0.02	0.47	0.89	0.01	0.02	0.47	0.89	0.01	0.02	0.00	0.00	0.47	0.89	0.01	0.02	0.47	0.89	0.01	0.02
NOG	0.65	0.92	0.64	0.94	0.65	0.92	0.64	0.94	0.01	0.02	0.00	0.03	0.64	0.94	0.65	0.92	0.64	0.94	0.65	0.92
OPN	0.00	0.02	0.32	0.89	0.00	0.02	0.32	0.89	0.01	0.04	0.00	0.02	0.32	0.89	0.00	0.02	0.32	0.89	0.00	0.02
SHH	0.35	0.88	0.33	0.89	0.35	0.88	0.33	0.89	0.00	0.03	0.01	0.12	0.33	0.89	0.35	0.88	0.33	0.89	0.35	0.88
Wnt	0.00	0.10	0.43	0.95	0.00	0.10	0.43	0.95	0.00	0.04	0.00	0.02	0.43	0.95	0.00	0.10	0.43	0.95	0.00	0.10

Table S5. Recurrence of attractors using different forms of equations. Alternative equation (b). 8 attractors are exactly obtained. The differentiated states S5 and S6 under this equation form are merged and become a limit cycle attractor. The average value along one period is listed here.

	S1	S2	S3	S4	S5, S6	S7	S8	S9	S10
Cyclin D/Cdk4,6	0.99	0.99	0.96	0.96	0.00	0.99	0.99	0.96	0.96
Cyclin E/Cdk2	0.91	0.91	0.00	0.00	0.00	0.91	0.91	0.00	0.00
E2F	0.95	0.95	0.00	0.00	0.00	0.95	0.95	0.00	0.00
Myc	0.05	0.05	0.05	0.05	0.00	0.05	0.05	0.05	0.05
p21	0.04	0.04	0.04	0.04	0.97	0.04	0.04	0.04	0.04
p27	0.04	0.04	0.04	0.04	0.98	0.04	0.04	0.04	0.04
pRb	0.99	0.99	0.95	0.95	0.00	0.99	0.99	0.95	0.95
Ras	1.00	1.00	1.00	1.00	0.98	1.00	1.00	1.00	1.00
Bad	0.00	0.00	0.00	0.00	0.00	0.00	0.00	0.00	0.00
Bax	0.00	0.00	0.00	0.00	0.01	0.00	0.00	0.00	0.00
Bcl-2	0.00	0.00	0.00	0.00	0.64	0.00	0.00	0.00	0.00
Bcl-xL	0.99	0.96	0.99	0.96	0.44	0.05	0.06	0.05	0.06
Bim	0.01	0.01	0.01	0.01	0.98	0.01	0.01	0.01	0.01
Caspase 3	0.00	0.00	0.00	0.00	0.00	0.94	0.94	0.94	0.94
Cytochrome C	0.00	0.00	0.00	0.00	0.00	0.93	0.93	0.93	0.93
Fas	0.00	0.00	0.00	0.00	0.00	0.00	0.00	0.00	0.00
XIAP	0.99	0.99	0.99	0.99	0.55	0.01	0.01	0.01	0.01
C/EBPα	0.00	0.00	0.00	0.00	0.01	0.00	0.00	0.00	0.00
C/EBPβ	0.99	0.99	0.99	0.99	0.05	0.99	0.99	0.99	0.99
Cdx2	0.00	0.01	0.00	0.01	0.94	0.01	0.00	0.01	0.00
Foxa1	0.05	0.05	0.05	0.05	0.05	0.05	0.05	0.05	0.05
Foxa2	0.01	0.01	0.01	0.01	0.37	0.01	0.01	0.01	0.01
Gata1/2	0.04	0.04	0.04	0.04	0.02	0.04	0.04	0.04	0.04
Gata4/6	0.01	0.00	0.01	0.00	0.05	0.00	0.01	0.00	0.01
Hes1	0.00	0.00	0.00	0.00	0.00	0.00	0.00	0.00	0.00
Hey2	0.00	0.00	0.00	0.00	0.00	0.00	0.00	0.00	0.00
HNF1	0.00	0.00	0.00	0.00	0.40	0.00	0.00	0.00	0.00
HNF4α	0.00	0.00	0.00	0.00	0.33	0.00	0.00	0.00	0.00
Hoxa10	0.99	0.01	0.99	0.01	0.03	0.01	0.99	0.01	0.99
Hoxa3	0.00	0.05	0.00	0.05	0.97	0.05	0.00	0.05	0.00
Hoxa5	0.95	0.00	0.95	0.00	0.97	0.00	0.95	0.00	0.95
Hoxa9	0.95	0.02	0.95	0.02	0.38	0.02	0.95	0.02	0.95
PGC-1	0.04	0.01	0.04	0.01	0.00	0.01	0.04	0.01	0.04
PRDM14	0.95	0.95	0.95	0.95	0.00	0.95	0.95	0.95	0.95
Pu.1	0.00	0.05	0.00	0.05	0.00	0.05	0.00	0.05	0.00
Runx1	0.05	0.98	0.05	0.98	0.03	0.98	0.05	0.98	0.05
Runx2	0.00	0.01	0.00	0.01	0.00	0.01	0.00	0.01	0.00
Sox2	0.99	1.00	0.99	1.00	0.01	1.00	0.99	1.00	0.99
Sox4	0.95	0.96	0.95	0.96	0.98	0.96	0.95	0.96	0.95
Sox7	0.00	0.05	0.00	0.05	0.98	0.05	0.00	0.05	0.00
Sox9	0.97	0.04	0.97	0.04	0.03	0.04	0.97	0.04	0.97
PPARγ	0.00	0.01	0.00	0.01	0.00	0.01	0.00	0.01	0.00
GR	0.00	0.00	0.00	0.00	0.03	0.00	0.00	0.00	0.00
NR4A	0.96	0.99	0.96	0.99	0.37	0.99	0.96	0.99	0.96
RARs	0.01	0.01	0.01	0.01	0.33	0.01	0.01	0.01	0.01
NR2F2	0.99	1.00	0.99	1.00	0.55	1.00	0.99	1.00	0.99
G-CSFR	0.00	0.00	0.00	0.00	0.00	0.00	0.00	0.00	0.00
IL-1	0.05	0.05	0.05	0.05	0.01	0.05	0.05	0.05	0.05
IL-10	0.00	0.00	0.00	0.00	0.00	0.00	0.00	0.00	0.00
IL-3	0.01	0.95	0.01	0.95	0.01	0.95	0.01	0.95	0.01
IL-6	0.05	0.05	0.05	0.05	0.23	0.05	0.05	0.05	0.05
iκB	0.01	0.01	0.01	0.01	0.50	0.01	0.01	0.01	0.01
NF-κB	0.01	0.01	0.01	0.01	0.02	0.01	0.01	0.01	0.01
Stat3	0.99	0.98	0.99	0.98	0.03	0.98	0.99	0.98	0.99
Stat5	0.96	0.05	0.96	0.05	0.32	0.05	0.96	0.05	0.96
TGF-β	0.00	0.01	0.00	0.01	0.03	0.01	0.00	0.01	0.00
TNF-α	0.00	0.00	0.00	0.00	0.00	0.00	0.00	0.00	0.00
BMP	0.01	0.01	0.01	0.01	0.01	0.01	0.01	0.01	0.01
CCK	1.00	1.00	1.00	1.00	0.96	1.00	1.00	1.00	1.00
EGFR	0.99	1.00	0.99	1.00	0.06	1.00	0.99	1.00	0.99
FGF2	0.95	0.00	0.95	0.00	0.00	0.00	0.95	0.00	0.95
FGF4/8	0.95	0.94	0.95	0.94	0.00	0.94	0.95	0.94	0.95
FGF7	0.00	0.00	0.00	0.00	0.00	0.00	0.00	0.00	0.00
FGF9	0.95	0.00	0.95	0.00	0.00	0.00	0.95	0.00	0.95
FGFR2	0.05	0.00	0.05	0.00	0.00	0.00	0.05	0.00	0.05
FGFR3	0.94	0.00	0.94	0.00	0.00	0.00	0.94	0.00	0.94
FGFR4	0.99	0.94	0.99	0.94	0.00	0.94	0.99	0.94	0.99
Gast	0.96	0.99	0.96	0.99	0.00	0.99	0.96	0.99	0.96
HGF	0.96	0.99	0.96	0.99	0.98	0.99	0.96	0.99	0.96
IR	0.95	0.99	0.95	0.99	0.00	0.99	0.95	0.99	0.95
VEGF	0.04	0.04	0.04	0.04	0.57	0.04	0.04	0.04	0.04
Akt	1.00	1.00	1.00	1.00	0.06	1.00	1.00	1.00	1.00
AP2	0.99	0.99	0.99	0.99	0.93	0.99	0.99	0.99	0.99
c-Jun	0.05	1.00	0.05	1.00	0.96	1.00	0.05	1.00	0.05
HIF	1.00	1.00	1.00	1.00	0.52	1.00	1.00	1.00	1.00
MAPK	1.00	0.99	1.00	0.99	0.05	0.99	1.00	0.99	1.00
Mkp2	0.95	0.00	0.95	0.00	0.00	0.00	0.95	0.00	0.95
PKA	0.99	0.99	0.99	0.99	0.05	0.99	0.99	0.99	0.99
PTEN	0.05	0.05	0.05	0.05	0.94	0.05	0.05	0.05	0.05
p53	0.01	0.01	0.01	0.01	0.04	0.01	0.01	0.01	0.01
E-Cadherin	0.00	0.00	0.00	0.00	0.24	0.00	0.00	0.00	0.00
HSPG2	0.00	0.00	0.00	0.00	0.00	0.00	0.00	0.00	0.00
Integrin	0.01	0.01	0.01	0.01	0.03	0.01	0.01	0.01	0.01
Notch	0.01	0.05	0.01	0.05	0.01	0.05	0.01	0.05	0.01
NGAL	0.00	0.95	0.00	0.95	0.00	0.95	0.00	0.95	0.00
NOG	0.99	0.99	0.99	0.99	0.00	0.99	0.99	0.99	0.99
OPN	0.00	0.95	0.00	0.95	0.00	0.95	0.00	0.95	0.00
SHH	0.95	0.96	0.95	0.96	0.01	0.96	0.95	0.96	0.95
Wnt	0.05	0.98	0.05	0.98	0.00	0.98	0.05	0.98	0.05

Table S6. Attractors: Boolean dynamics *vs.* differential equations. Inside each slot is comparison of corresponding attractors obtained separately from Boolean dynamics (left) and differential equations (right). All attractors from differential equations except S6 are obtained by Boolean dynamics. The comparison result demonstrates that our differential equations generate consistent results with Boolean network modeling.

	S1		S2		S3		S4		S5		S7		S8		S9		S10	
	E2	E1	E2	E1	E2	E1	E2	E1	E2	E1	E2	E1	E2	E1	E2	E1	E2	E1
Cyclin D/Cdk4,6	1	0.93	1	0.93	1	0.88	1	0.89	0	0.00	1	0.93	1	0.93	1	0.89	1	0.88
Cyclin E/Cdk2	1	0.85	1	0.84	0	0.01	0	0.01	0	0.00	1	0.84	1	0.85	0	0.01	0	0.01
E2F	1	0.86	1	0.86	0	0.01	0	0.01	0	0.00	1	0.86	1	0.86	0	0.01	0	0.01
Myc	0	0.10	0	0.10	0	0.10	0	0.10	0	0.00	0	0.10	0	0.10	0	0.10	0	0.10
p21	0	0.09	0	0.09	0	0.09	0	0.09	1	0.90	0	0.09	0	0.09	0	0.09	0	0.09
p27	0	0.09	0	0.09	0	0.09	0	0.09	1	0.90	0	0.09	0	0.09	0	0.09	0	0.09
pRb	1	0.93	1	0.93	1	0.87	1	0.88	0	0.00	1	0.93	1	0.93	1	0.88	1	0.87
Ras	1	0.95	1	0.96	1	0.95	1	0.96	1	0.89	1	0.96	1	0.95	1	0.96	1	0.95
Bad	0	0.00	0	0.00	0	0.00	0	0.00	0	0.00	0	0.00	0	0.00	0	0.00	0	0.00
Bax	0	0.00	0	0.00	0	0.00	0	0.00	0	0.07	0	0.00	0	0.00	0	0.00	0	0.00
Bcl-2	0	0.01	0	0.02	0	0.01	0	0.02	1	0.82	0	0.00	0	0.00	0	0.00	0	0.00
Bcl-xL	1	0.93	1	0.90	1	0.93	1	0.90	0	0.05	0	0.13	0	0.13	0	0.13	0	0.13
Bim	0	0.05	0	0.05	0	0.05	0	0.05	1	0.95	0	0.05	0	0.05	0	0.05	0	0.05
Caspase 3	0	0.00	0	0.00	0	0.00	0	0.00	0	0.00	1	0.85	1	0.85	1	0.85	1	0.85
Cytochrome C	0	0.00	0	0.00	0	0.00	0	0.00	0	0.00	1	0.84	1	0.83	1	0.84	1	0.83
Fas	0	0.00	0	0.00	0	0.00	0	0.00	0	0.00	0	0.00	0	0.00	0	0.00	0	0.00
XIAP	1	0.95	1	0.95	1	0.95	1	0.95	0	0.09	0	0.07	0	0.07	0	0.07	0	0.07
C/EBPα	0	0.02	0	0.03	0	0.02	0	0.03	0	0.11	0	0.03	0	0.02	0	0.03	0	0.02
C/EBPβ	1	0.94	1	0.93	1	0.94	1	0.93	0	0.13	1	0.93	1	0.94	1	0.93	1	0.94
Cdx2	0	0.04	0	0.05	0	0.04	0	0.05	1	0.88	0	0.05	0	0.04	0	0.05	0	0.04
Foxa1	0	0.12	0	0.11	0	0.12	0	0.11	0	0.11	0	0.11	0	0.12	0	0.11	0	0.12
Foxa2	0	0.05	0	0.05	0	0.05	0	0.05	0	0.03	0	0.05	0	0.05	0	0.05	0	0.05
Gata1/2	0	0.10	0	0.09	0	0.10	0	0.09	0	0.07	0	0.09	0	0.10	0	0.09	0	0.10
Gata4/6	0	0.05	0	0.04	0	0.05	0	0.04	0	0.13	0	0.04	0	0.05	0	0.04	0	0.05
Hes1	0	0.00	0	0.01	0	0.00	0	0.01	0	0.02	0	0.01	0	0.00	0	0.01	0	0.00
Hey2	0	0.00	0	0.01	0	0.00	0	0.01	0	0.00	0	0.01	0	0.00	0	0.01	0	0.00
HNF1	0	0.00	0	0.01	0	0.00	0	0.01	1	0.86	0	0.01	0	0.00	0	0.01	0	0.00
HNF4α	0	0.02	0	0.02	0	0.02	0	0.02	1	0.87	0	0.02	0	0.02	0	0.02	0	0.02
Hoxa10	1	0.92	0	0.01	1	0.92	0	0.01	0	0.00	0	0.01	1	0.92	0	0.01	1	0.92
Hoxa3	0	0.00	0	0.10	0	0.00	0	0.10	1	0.84	0	0.10	0	0.00	0	0.10	0	0.00
Hoxa5	1	0.87	0	0.00	1	0.87	0	0.00	1	0.85	0	0.00	1	0.87	0	0.00	1	0.87
Hoxa9	1	0.86	0	0.02	1	0.86	0	0.02	0	0.03	0	0.02	1	0.86	0	0.02	1	0.86
PGC-1	0	0.09	0	0.05	0	0.09	0	0.05	0	0.00	0	0.05	0	0.09	0	0.05	0	0.09
PRDM14	1	0.90	1	0.90	1	0.90	1	0.90	0	0.03	1	0.90	1	0.90	1	0.90	1	0.90
Pu.1	0	0.00	0	0.11	0	0.00	0	0.11	0	0.00	0	0.11	0	0.00	0	0.11	0	0.00
Runx1	0	0.12	1	0.93	0	0.12	1	0.93	0	0.14	1	0.93	0	0.12	1	0.93	0	0.12
Runx2	0	0.03	0	0.05	0	0.03	0	0.05	0	0.00	0	0.05	0	0.03	0	0.05	0	0.03
Sox2	1	0.94	1	0.95	1	0.94	1	0.95	0	0.06	1	0.95	1	0.94	1	0.95	1	0.94
Sox4	1	0.86	1	0.90	1	0.86	1	0.90	1	0.87	1	0.90	1	0.86	1	0.90	1	0.86
Sox7	0	0.00	0	0.11	0	0.00	0	0.11	1	0.92	0	0.11	0	0.00	0	0.11	0	0.00
Sox9	1	0.94	0	0.10	1	0.94	0	0.10	0	0.12	0	0.10	1	0.94	0	0.10	1	0.94
PPARγ	0	0.04	0	0.05	0	0.04	0	0.05	0	0.00	0	0.05	0	0.04	0	0.05	0	0.04
GR	0	0.00	0	0.00	0	0.00	0	0.00	0	0.11	0	0.00	0	0.00	0	0.00	0	0.00
NR4A	1	0.89	1	0.94	1	0.89	1	0.94	0	0.11	1	0.94	1	0.89	1	0.94	1	0.89
RARs	0	0.05	0	0.05	0	0.05	0	0.05	0	0.11	0	0.05	0	0.05	0	0.05	0	0.05
NR2F2	1	0.94	1	0.96	1	0.94	1	0.96	1	0.87	1	0.96	1	0.94	1	0.96	1	0.94
G-CSFR	0	0.00	0	0.01	0	0.00	0	0.01	0	0.03	0	0.01	0	0.00	0	0.01	0	0.00
IL-1	0	0.09	0	0.09	0	0.09	0	0.09	0	0.03	0	0.09	0	0.09	0	0.09	0	0.09
IL-10	0	0.00	0	0.00	0	0.00	0	0.00	0	0.00	0	0.00	0	0.00	0	0.00	0	0.00
IL-3	0	0.03	1	0.89	0	0.03	1	0.89	0	0.04	1	0.89	0	0.03	1	0.89	0	0.03
IL-6	0	0.10	0	0.10	0	0.10	0	0.10	0	0.11	0	0.10	0	0.10	0	0.10	0	0.10
iκB	0	0.05	0	0.05	0	0.05	0	0.05	0	0.05	0	0.05	0	0.05	0	0.05	0	0.05
NF-κB	0	0.06	0	0.05	0	0.06	0	0.05	0	0.11	0	0.05	0	0.06	0	0.05	0	0.06
Stat3	1	0.95	1	0.96	1	0.95	1	0.96	0	0.14	1	0.96	1	0.95	1	0.96	1	0.95
Stat5	1	0.85	0	0.10	1	0.85	0	0.10	0	0.11	0	0.10	1	0.85	0	0.10	1	0.85
TGF-β	0	0.04	0	0.05	0	0.04	0	0.05	0	0.12	0	0.05	0	0.04	0	0.05	0	0.04
TNF-α	0	0.01	0	0.01	0	0.01	0	0.01	0	0.01	0	0.01	0	0.01	0	0.01	0	0.01
BMP	0	0.05	0	0.05	0	0.05	0	0.05	0	0.00	0	0.05	0	0.05	0	0.05	0	0.05
CCK	1	0.96	1	0.96	1	0.96	1	0.96	1	0.87	1	0.96	1	0.96	1	0.96	1	0.96
EGFR	1	0.93	1	0.95	1	0.93	1	0.95	0	0.13	1	0.95	1	0.93	1	0.95	1	0.93
FGF2	1	0.89	0	0.00	1	0.89	0	0.00	0	0.00	0	0.00	1	0.89	0	0.00	1	0.89
FGF4/8	1	0.87	1	0.87	1	0.87	1	0.87	0	0.00	1	0.87	1	0.87	1	0.87	1	0.87
FGF7	0	0.01	0	0.01	0	0.01	0	0.01	0	0.00	0	0.01	0	0.01	0	0.01	0	0.01
FGF9	1	0.89	0	0.01	1	0.89	0	0.01	0	0.02	0	0.01	1	0.89	0	0.01	1	0.89
FGFR2	0	0.11	0	0.00	0	0.11	0	0.00	0	0.00	0	0.00	0	0.11	0	0.00	0	0.11
FGFR3	1	0.88	0	0.00	1	0.88	0	0.00	0	0.00	0	0.00	1	0.88	0	0.00	1	0.88
FGFR4	1	0.93	1	0.87	1	0.93	1	0.87	0	0.00	1	0.87	1	0.93	1	0.87	1	0.93
Gast	1	0.89	1	0.95	1	0.89	1	0.95	0	0.02	1	0.95	1	0.89	1	0.95	1	0.89
HGF	1	0.90	1	0.95	1	0.90	1	0.95	1	0.83	1	0.95	1	0.90	1	0.95	1	0.90
IR	1	0.87	1	0.93	1	0.87	1	0.93	0	0.02	1	0.93	1	0.87	1	0.93	1	0.87
VEGF	0	0.10	0	0.11	0	0.10	0	0.11	1	0.82	0	0.11	0	0.10	0	0.11	0	0.10
Akt	1	0.97	1	0.98	1	0.97	1	0.98	0	0.14	1	0.98	1	0.97	1	0.98	1	0.97
AP2	1	0.94	1	0.94	1	0.94	1	0.94	1	0.87	1	0.94	1	0.94	1	0.94	1	0.94
c-Jun	0	0.12	1	0.97	0	0.12	1	0.97	1	0.85	1	0.97	0	0.12	1	0.97	0	0.12
HIF	1	0.96	1	0.96	1	0.96	1	0.96	0	0.06	1	0.96	1	0.96	1	0.96	1	0.96
MAPK	1	0.95	1	0.94	1	0.95	1	0.94	0	0.14	1	0.94	1	0.95	1	0.94	1	0.95
Mkp2	1	0.89	0	0.00	1	0.89	0	0.00	0	0.00	0	0.00	1	0.89	0	0.00	1	0.89
PKA	1	0.94	1	0.95	1	0.94	1	0.95	0	0.06	1	0.95	1	0.94	1	0.95	1	0.94
PTEN	0	0.09	0	0.09	0	0.09	0	0.09	1	0.83	0	0.09	0	0.09	0	0.09	0	0.09
p53	0	0.05	0	0.05	0	0.05	0	0.05	0	0.12	0	0.05	0	0.05	0	0.05	0	0.05
E-Cadherin	0	0.02	0	0.02	0	0.02	0	0.02	1	0.87	0	0.02	0	0.02	0	0.02	0	0.02
HSPG2	0	0.00	0	0.00	0	0.00	0	0.00	0	0.02	0	0.00	0	0.00	0	0.00	0	0.00
Integrin	0	0.06	0	0.06	0	0.06	0	0.06	0	0.11	0	0.06	0	0.06	0	0.06	0	0.06
Notch	0	0.06	0	0.10	0	0.06	0	0.10	0	0.05	0	0.10	0	0.06	0	0.10	0	0.06
NGAL	0	0.02	1	0.89	0	0.02	1	0.89	0	0.02	1	0.89	0	0.02	1	0.89	0	0.02
NOG	1	0.92	1	0.94	1	0.92	1	0.94	0	0.02	1	0.94	1	0.92	1	0.94	1	0.92
OPN	0	0.02	1	0.89	0	0.02	1	0.89	0	0.04	1	0.89	0	0.02	1	0.89	0	0.02
SHH	1	0.88	1	0.89	1	0.88	1	0.89	0	0.03	1	0.89	1	0.88	1	0.89	1	0.88
Wnt	0	0.10	1	0.95	0	0.10	1	0.95	0	0.04	1	0.95	0	0.10	1	0.95	0	0.10

Table S7. Unstable fixed points from differential equations. We obtain 21 unstable fixed points where 14 of them are saddle points. Among them, 12 saddle points and 6 other unstable fixed points together with 8 attractors form a face-centered cubic type topological structure in the phase space. In addition, there is an unstable point at the body-center as well. These 21 unstable points are first obtained while calculating with 100,000 random initial positions using *Newtonian iteration method*. We further run the program with 1,000,000 and more initial positions, acquiring an identical result.

	U1	U2	U3	U4	U5	U6	U7	U8	U9	U10	U11	U12	U13	U14	U15	U16	U17	U18	U19	U20	U21
Cyclin D/Cdk4,6	0.90	0.87	0.89	0.92	0.93	0.89	0.02	0.88	0.93	0.00	0.90	0.87	0.92	0.89	0.88	0.90	0.87	0.92	0.89	0.88	0.88
Cyclin E/Cdk2	0.41	0.01	0.01	0.84	0.84	0.41	0.00	0.01	0.85	0.00	0.41	0.01	0.84	0.41	0.40	0.41	0.01	0.84	0.41	0.40	0.40
E2F	0.41	0.01	0.01	0.86	0.86	0.41	0.00	0.01	0.86	0.00	0.41	0.01	0.86	0.41	0.41	0.41	0.01	0.86	0.41	0.41	0.41
Myc	0.10	0.10	0.10	0.10	0.10	0.10	0.02	0.10	0.10	0.01	0.10	0.10	0.10	0.10	0.10	0.10	0.10	0.10	0.10	0.10	0.10
p21	0.09	0.09	0.09	0.09	0.09	0.09	0.74	0.09	0.09	0.91	0.09	0.09	0.09	0.09	0.09	0.09	0.09	0.09	0.09	0.09	0.09
p27	0.09	0.09	0.09	0.09	0.09	0.09	0.72	0.09	0.09	0.93	0.09	0.09	0.09	0.09	0.09	0.09	0.09	0.09	0.09	0.09	0.09
pRb	0.89	0.87	0.88	0.93	0.93	0.89	0.00	0.87	0.93	0.00	0.89	0.87	0.93	0.89	0.88	0.89	0.87	0.93	0.89	0.88	0.88
Ras	0.96	0.95	0.96	0.95	0.96	0.95	0.80	0.95	0.95	0.03	0.96	0.95	0.95	0.95	0.95	0.96	0.95	0.95	0.95	0.95	0.95
Bad	0.00	0.00	0.00	0.00	0.00	0.00	0.00	0.00	0.00	0.10	0.00	0.00	0.00	0.00	0.00	0.00	0.00	0.00	0.00	0.00	0.00
Bax	0.00	0.00	0.00	0.00	0.00	0.00	0.09	0.00	0.00	0.81	0.00	0.00	0.00	0.00	0.00	0.00	0.00	0.00	0.00	0.00	0.00
Bcl-2	0.02	0.01	0.00	0.01	0.00	0.01	0.63	0.00	0.00	0.00	0.00	0.00	0.00	0.00	0.01	0.00	0.00	0.00	0.00	0.00	0.00
Bcl-xL	0.90	0.89	0.28	0.89	0.28	0.93	0.27	0.28	0.28	0.00	0.13	0.13	0.13	0.13	0.89	0.28	0.28	0.28	0.28	0.13	0.28
Bim	0.05	0.05	0.05	0.05	0.05	0.05	0.82	0.05	0.05	0.99	0.05	0.05	0.05	0.05	0.05	0.05	0.05	0.05	0.05	0.05	0.05
Caspase 3	0.00	0.00	0.61	0.00	0.61	0.00	0.00	0.62	0.62	0.00	0.85	0.85	0.85	0.85	0.00	0.61	0.61	0.61	0.62	0.85	0.61
Cytochrome C	0.00	0.00	0.57	0.00	0.57	0.00	0.00	0.58	0.58	0.10	0.84	0.84	0.84	0.83	0.00	0.57	0.57	0.57	0.58	0.84	0.57
Fas	0.00	0.00	0.00	0.00	0.00	0.00	0.00	0.00	0.00	0.00	0.00	0.00	0.00	0.00	0.00	0.00	0.00	0.00	0.00	0.00	0.00
XIAP	0.95	0.95	0.19	0.95	0.19	0.95	0.29	0.18	0.18	0.85	0.07	0.07	0.07	0.07	0.95	0.19	0.19	0.19	0.18	0.07	0.19
C/EBPα	0.03	0.03	0.03	0.03	0.03	0.02	0.15	0.02	0.02	0.05	0.03	0.03	0.03	0.02	0.03	0.03	0.03	0.03	0.02	0.03	0.03
C/EBPβ	0.93	0.94	0.93	0.94	0.93	0.94	0.15	0.94	0.94	0.11	0.93	0.94	0.94	0.94	0.94	0.93	0.94	0.94	0.94	0.94	0.94
Cdx2	0.05	0.04	0.05	0.04	0.05	0.04	0.68	0.04	0.04	0.37	0.05	0.04	0.04	0.04	0.04	0.05	0.04	0.04	0.04	0.04	0.04
Foxa1	0.11	0.40	0.11	0.40	0.11	0.12	0.11	0.12	0.12	0.82	0.11	0.40	0.40	0.12	0.40	0.11	0.40	0.40	0.12	0.40	0.40
Foxa2	0.05	0.05	0.05	0.05	0.05	0.05	0.13	0.05	0.05	0.85	0.05	0.05	0.05	0.05	0.05	0.05	0.05	0.05	0.05	0.05	0.05
Gata1/2	0.09	0.20	0.09	0.20	0.09	0.10	0.10	0.10	0.10	0.24	0.09	0.20	0.20	0.10	0.20	0.09	0.20	0.20	0.10	0.20	0.20
Gata4/6	0.04	0.06	0.04	0.06	0.04	0.05	0.16	0.05	0.05	0.17	0.04	0.06	0.06	0.05	0.06	0.04	0.06	0.06	0.05	0.06	0.06
Hes1	0.01	0.01	0.01	0.01	0.01	0.00	0.13	0.00	0.00	0.02	0.01	0.01	0.01	0.00	0.01	0.01	0.01	0.01	0.00	0.01	0.01
Hey2	0.01	0.01	0.01	0.01	0.01	0.00	0.01	0.00	0.00	0.00	0.01	0.01	0.01	0.00	0.01	0.01	0.01	0.01	0.00	0.01	0.01
HNF1	0.01	0.01	0.01	0.01	0.01	0.00	0.57	0.00	0.00	0.03	0.01	0.01	0.01	0.00	0.01	0.01	0.01	0.01	0.00	0.01	0.01
HNF4α	0.02	0.02	0.02	0.02	0.02	0.02	0.52	0.02	0.02	0.14	0.02	0.02	0.02	0.02	0.02	0.02	0.02	0.02	0.02	0.02	0.02
Hoxa10	0.01	0.68	0.01	0.68	0.01	0.92	0.05	0.92	0.92	0.00	0.01	0.68	0.68	0.92	0.68	0.01	0.68	0.68	0.92	0.68	0.68
Hoxa3	0.10	0.01	0.10	0.01	0.10	0.00	0.74	0.00	0.00	0.87	0.10	0.01	0.01	0.00	0.01	0.10	0.01	0.01	0.00	0.01	0.01
Hoxa5	0.00	0.39	0.00	0.39	0.00	0.87	0.73	0.87	0.87	0.87	0.00	0.39	0.39	0.87	0.39	0.00	0.39	0.39	0.87	0.39	0.39
Hoxa9	0.02	0.49	0.02	0.49	0.02	0.86	0.17	0.86	0.86	0.00	0.02	0.49	0.49	0.86	0.49	0.02	0.49	0.49	0.86	0.49	0.49
PGC-1	0.05	0.09	0.05	0.09	0.05	0.09	0.01	0.09	0.09	0.00	0.05	0.09	0.09	0.09	0.09	0.05	0.09	0.09	0.09	0.09	0.09
PRDM14	0.90	0.87	0.90	0.87	0.90	0.90	0.09	0.90	0.90	0.00	0.90	0.87	0.87	0.90	0.87	0.90	0.87	0.87	0.90	0.87	0.87
Pu.1	0.11	0.21	0.11	0.21	0.11	0.00	0.01	0.00	0.00	0.00	0.11	0.21	0.21	0.00	0.21	0.11	0.21	0.21	0.00	0.21	0.21
Runx1	0.93	0.45	0.93	0.45	0.93	0.12	0.17	0.12	0.12	0.07	0.93	0.45	0.45	0.12	0.45	0.93	0.45	0.45	0.12	0.45	0.45
Runx2	0.05	0.03	0.05	0.03	0.05	0.03	0.01	0.03	0.03	0.00	0.05	0.03	0.03	0.03	0.03	0.05	0.03	0.03	0.03	0.03	0.03
Sox2	0.95	0.93	0.95	0.93	0.95	0.94	0.10	0.94	0.94	0.09	0.95	0.93	0.93	0.94	0.93	0.95	0.93	0.93	0.94	0.93	0.93
Sox4	0.90	0.50	0.90	0.50	0.90	0.86	0.85	0.86	0.86	0.25	0.90	0.50	0.50	0.86	0.50	0.90	0.50	0.50	0.86	0.50	0.50
Sox7	0.11	0.01	0.11	0.01	0.11	0.00	0.87	0.00	0.00	0.30	0.11	0.01	0.01	0.00	0.01	0.11	0.01	0.01	0.00	0.01	0.01
Sox9	0.10	0.90	0.10	0.90	0.10	0.94	0.15	0.94	0.94	0.12	0.10	0.90	0.90	0.94	0.90	0.10	0.90	0.90	0.94	0.90	0.90
PPARγ	0.05	0.05	0.05	0.05	0.05	0.04	0.02	0.04	0.04	0.00	0.05	0.05	0.05	0.04	0.05	0.05	0.05	0.05	0.04	0.05	0.05
GR	0.00	0.00	0.00	0.00	0.00	0.00	0.18	0.00	0.00	0.02	0.00	0.00	0.00	0.00	0.00	0.00	0.00	0.00	0.00	0.00	0.00
NR4A	0.94	0.89	0.94	0.89	0.94	0.89	0.29	0.89	0.89	0.00	0.94	0.89	0.89	0.89	0.89	0.94	0.89	0.89	0.89	0.89	0.89
RARs	0.05	0.05	0.05	0.05	0.05	0.05	0.22	0.05	0.05	0.85	0.05	0.05	0.05	0.05	0.05	0.05	0.05	0.05	0.05	0.05	0.05
NR2F2	0.96	0.94	0.96	0.94	0.96	0.94	0.62	0.94	0.94	0.12	0.96	0.94	0.94	0.94	0.94	0.96	0.94	0.94	0.94	0.94	0.94
G-CSFR	0.01	0.08	0.01	0.08	0.01	0.00	0.08	0.00	0.00	0.00	0.01	0.08	0.08	0.00	0.08	0.01	0.08	0.08	0.00	0.08	0.08
IL-1	0.09	0.09	0.09	0.09	0.09	0.09	0.10	0.09	0.09	0.02	0.09	0.09	0.09	0.09	0.09	0.09	0.09	0.09	0.09	0.09	0.09
IL-10	0.00	0.00	0.00	0.00	0.00	0.00	0.00	0.00	0.00	0.00	0.00	0.00	0.00	0.00	0.00	0.00	0.00	0.00	0.00	0.00	0.00
IL-3	0.89	0.50	0.89	0.50	0.89	0.03	0.08	0.03	0.03	0.13	0.89	0.50	0.50	0.03	0.50	0.89	0.50	0.50	0.03	0.50	0.50
IL-6	0.10	0.10	0.10	0.10	0.10	0.10	0.32	0.10	0.10	0.12	0.10	0.10	0.10	0.10	0.10	0.10	0.10	0.10	0.10	0.10	0.10
iκB	0.05	0.05	0.05	0.05	0.05	0.05	0.28	0.05	0.05	0.02	0.05	0.05	0.05	0.05	0.05	0.05	0.05	0.05	0.05	0.05	0.05
NF-κB	0.05	0.06	0.05	0.06	0.05	0.06	0.19	0.06	0.06	0.05	0.05	0.06	0.06	0.06	0.06	0.05	0.06	0.06	0.06	0.06	0.06
Stat3	0.96	0.89	0.96	0.89	0.96	0.95	0.22	0.95	0.95	0.03	0.96	0.89	0.89	0.95	0.89	0.96	0.89	0.89	0.95	0.89	0.89
Stat5	0.10	0.46	0.10	0.46	0.10	0.85	0.28	0.85	0.85	0.02	0.10	0.46	0.46	0.85	0.46	0.10	0.46	0.46	0.85	0.46	0.46
TGF-β	0.05	0.05	0.05	0.05	0.05	0.04	0.24	0.04	0.04	0.13	0.05	0.05	0.05	0.04	0.05	0.05	0.05	0.05	0.04	0.05	0.05
TNF-α	0.01	0.01	0.01	0.01	0.01	0.01	0.07	0.01	0.01	0.00	0.01	0.01	0.01	0.01	0.01	0.01	0.01	0.01	0.01	0.01	0.01
BMP	0.05	0.05	0.05	0.05	0.05	0.05	0.03	0.05	0.05	0.04	0.05	0.05	0.05	0.05	0.05	0.05	0.05	0.05	0.05	0.05	0.05
CCK	0.96	0.96	0.96	0.96	0.96	0.96	0.76	0.96	0.96	0.37	0.96	0.96	0.96	0.96	0.96	0.96	0.96	0.96	0.96	0.96	0.96
EGFR	0.95	0.90	0.95	0.90	0.95	0.93	0.21	0.93	0.93	0.02	0.95	0.90	0.90	0.93	0.90	0.95	0.90	0.90	0.93	0.90	0.90
FGF2	0.00	0.76	0.00	0.76	0.00	0.89	0.02	0.89	0.89	0.00	0.00	0.76	0.76	0.89	0.76	0.00	0.76	0.76	0.89	0.76	0.76
FGF4/8	0.87	0.87	0.87	0.87	0.87	0.87	0.02	0.87	0.87	0.02	0.87	0.87	0.87	0.87	0.87	0.87	0.87	0.87	0.87	0.87	0.87
FGF7	0.01	0.01	0.01	0.01	0.01	0.01	0.03	0.01	0.01	0.00	0.01	0.01	0.01	0.01	0.01	0.01	0.01	0.01	0.01	0.01	0.01
FGF9	0.01	0.88	0.01	0.88	0.01	0.89	0.03	0.89	0.89	0.02	0.01	0.88	0.88	0.89	0.88	0.01	0.88	0.88	0.89	0.88	0.88
FGFR2	0.00	0.11	0.00	0.11	0.00	0.11	0.00	0.11	0.11	0.00	0.00	0.11	0.11	0.11	0.11	0.00	0.11	0.11	0.11	0.11	0.11
FGFR3	0.00	0.87	0.00	0.87	0.00	0.88	0.00	0.88	0.88	0.00	0.00	0.87	0.87	0.88	0.87	0.00	0.87	0.87	0.88	0.87	0.87
FGFR4	0.87	0.93	0.87	0.93	0.87	0.93	0.00	0.93	0.93	0.00	0.87	0.93	0.93	0.93	0.93	0.87	0.93	0.93	0.93	0.93	0.93
Gast	0.95	0.89	0.95	0.89	0.95	0.89	0.13	0.89	0.89	0.08	0.95	0.89	0.89	0.89	0.89	0.95	0.89	0.89	0.89	0.89	0.89
HGF	0.95	0.90	0.95	0.90	0.95	0.90	0.69	0.90	0.90	0.29	0.95	0.90	0.90	0.90	0.90	0.95	0.90	0.90	0.90	0.90	0.90
IR	0.93	0.87	0.93	0.87	0.93	0.87	0.05	0.87	0.87	0.07	0.93	0.87	0.87	0.87	0.87	0.93	0.87	0.87	0.87	0.87	0.87
VEGF	0.11	0.10	0.11	0.10	0.11	0.10	0.64	0.10	0.10	0.01	0.11	0.10	0.10	0.10	0.10	0.11	0.10	0.10	0.10	0.10	0.10
Akt	0.98	0.97	0.98	0.97	0.98	0.97	0.23	0.97	0.97	0.10	0.98	0.97	0.97	0.97	0.97	0.98	0.97	0.97	0.97	0.97	0.97
AP2	0.94	0.94	0.94	0.94	0.94	0.94	0.72	0.94	0.94	0.86	0.94	0.94	0.94	0.94	0.94	0.94	0.94	0.94	0.94	0.94	0.94
c-Jun	0.97	0.18	0.97	0.18	0.97	0.12	0.76	0.12	0.12	0.35	0.97	0.18	0.18	0.12	0.18	0.97	0.18	0.18	0.12	0.18	0.18
HIF	0.96	0.95	0.96	0.95	0.96	0.96	0.32	0.96	0.96	0.00	0.96	0.95	0.95	0.96	0.95	0.96	0.95	0.95	0.96	0.95	0.95
MAPK	0.94	0.94	0.94	0.94	0.94	0.95	0.21	0.95	0.95	0.00	0.94	0.94	0.94	0.95	0.94	0.94	0.94	0.94	0.95	0.94	0.94
Mkp2	0.00	0.76	0.00	0.76	0.00	0.89	0.00	0.89	0.89	0.00	0.00	0.76	0.76	0.89	0.76	0.00	0.76	0.76	0.89	0.76	0.76
PKA	0.95	0.94	0.95	0.94	0.95	0.94	0.26	0.94	0.94	0.00	0.95	0.94	0.94	0.94	0.94	0.95	0.94	0.94	0.94	0.94	0.94
PTEN	0.09	0.11	0.09	0.11	0.09	0.09	0.67	0.09	0.09	0.92	0.09	0.11	0.11	0.09	0.11	0.09	0.11	0.11	0.09	0.11	0.11
p53	0.05	0.08	0.05	0.08	0.05	0.05	0.12	0.05	0.05	0.82	0.05	0.08	0.08	0.05	0.08	0.05	0.08	0.08	0.05	0.08	0.08
E-Cadherin	0.02	0.02	0.02	0.02	0.02	0.02	0.49	0.02	0.02	0.91	0.02	0.02	0.02	0.02	0.02	0.02	0.02	0.02	0.02	0.02	0.02
HSPG2	0.00	0.00	0.00	0.00	0.00	0.00	0.12	0.00	0.00	0.02	0.00	0.00	0.00	0.00	0.00	0.00	0.00	0.00	0.00	0.00	0.00
Integrin	0.06	0.06	0.06	0.06	0.06	0.06	0.12	0.06	0.06	0.00	0.06	0.06	0.06	0.06	0.06	0.06	0.06	0.06	0.06	0.06	0.06
Notch	0.10	0.10	0.10	0.10	0.10	0.06	0.10	0.06	0.06	0.02	0.10	0.10	0.10	0.06	0.10	0.10	0.10	0.10	0.06	0.10	0.10
NGAL	0.89	0.48	0.89	0.48	0.89	0.02	0.05	0.02	0.02	0.00	0.89	0.48	0.48	0.02	0.48	0.89	0.48	0.48	0.02	0.48	0.48
NOG	0.94	0.92	0.94	0.92	0.94	0.92	0.05	0.92	0.92	0.08	0.94	0.92	0.92	0.92	0.92	0.94	0.92	0.92	0.92	0.92	0.92
OPN	0.89	0.48	0.89	0.48	0.89	0.02	0.15	0.02	0.02	0.02	0.89	0.48	0.48	0.02	0.48	0.89	0.48	0.48	0.02	0.48	0.48
SHH	0.89	0.88	0.89	0.88	0.89	0.88	0.13	0.88	0.88	0.12	0.89	0.88	0.88	0.88	0.88	0.89	0.88	0.88	0.88	0.88	0.88
Wnt	0.95	0.12	0.95	0.12	0.95	0.10	0.07	0.10	0.10	0.17	0.95	0.12	0.12	0.10	0.12	0.95	0.12	0.12	0.10	0.12	0.12

	Saddle<br>U1	Saddle<br>U2	Saddle<br>U3	Saddle<br>U4	Saddle<br>U5	Saddle<br>U6	Saddle<br>U7	Saddle<br>U8	Saddle<br>U9	Saddle<br>U10	Saddle<br>U11	Saddle<br>U12	Saddle<br>U13	Saddle<br>U14	U15	U16	U17	U18	U19	U20	U21
Connected Stable Points & Times (Total 1000 perturbations)	S1 515<br>S2 485	S1 486<br>S3 514	S1 483<br>S7 517	S2 534<br>S4 466	S2 488<br>S8 512	S3 516<br>S4 484	S3 491<br>S5 509	S3 505<br>S10 495	S4 486<br>S9 514	S5 481<br>S6 519	S7 493<br>S8 507	S7 464<br>S10 536	S8 497<br>S9 503	S9 539<br>S10 461	S1 264<br>S2 258<br>S3 229<br>S4 249	S1 240<br>S2 247<br>S7 253<br>S8 260	S1 244<br>S3 248<br>S7 254<br>S10 254	S2 227<br>S4 264<br>S8 241<br>S9 268	S3 266<br>S4 228<br>S9 252<br>S10 254	S7 265<br>S8 261<br>S9 252<br>S10 222	S1 113<br>S2 99<br>S3 130<br>S4 143<br>S7 133<br>S8 132<br>S9 112<br>S10 138
